# Probing the Influence of Linker Length and Flexibility in the Design and Synthesis of New Trehalase Inhibitors

**DOI:** 10.3390/molecules23020436

**Published:** 2018-02-16

**Authors:** Giampiero D’Adamio, Matilde Forcella, Paola Fusi, Paolo Parenti, Camilla Matassini, Xhenti Ferhati, Costanza Vanni, Francesca Cardona

**Affiliations:** 1 Department of Chemistry ‘Ugo Schiff’, University of Florence, via della Lastruccia 3-13, I-50019 Sesto Fiorentino (FI), Italy; giampiero_dadamio@hotmail.it (G.D.); mage90@hotmail.it (X.F.); costanza.vanni@unifi.it (C.V.); 2 Department of Biotechnology and Biosciences, University of Milano-Bicocca, Piazza della Scienza 2, 20126 Milano, Italy; matilde.forcella@unimib.it (M.F.); paola.fusi@unimib.it (P.F.); 3 Department of Earth and Environmental Sciences, University of Milano-Bicocca, Piazza della Scienza 1, 20126 Milano, Italy; paolo.parenti@unimib.it; 4 Associated with CNR-INO, Via N. Carrara 1, I-50019 Sesto Fiorentino (FI), Italy; 5 Associated with Consorzio Interuniversitario Nazionale di ricerca in Metodologie e Processi Innovativi di Sintesi (CINMPIS), Università di Bari, 70125 Bari, Italy.

**Keywords:** iminosugars, trehalase inhibitors, pseudodisaccharides, pyrrolizidines, pyrrolidines, glycosylation reaction

## Abstract

This work aims to synthesize new trehalase inhibitors selective towards the insect trehalase versus the porcine trehalase, in view of their application as potentially non-toxic insecticides and fungicides. The synthesis of a new pseudodisaccharide mimetic **8**, by means of a stereoselective α-glucosylation of the key pyrrolizidine intermediate **13**, was accomplished. The activity of compound **8** as trehalase inhibitor towards *C. riparius* trehalase was evaluated and the results showed that **8** was active in the μM range and showed a good selectivity towards the insect trehalase. To reduce the overall number of synthetic steps, simpler and more flexible disaccharide mimetics **9**–**11** bearing a pyrrolidine nucleus instead of the pyrrolizidine core were synthesized. The biological data showed the key role of the linker chain’s length in inducing inhibitory properties, since only compounds **9** (α,β-mixture), bearing a two-carbon atom linker chain, maintained activity as trehalase inhibitors. A proper change in the glucosyl donor-protecting groups allowed the stereoselective synthesis of the β-glucoside **9**β, which was active in the low micromolar range (IC_50_ = 0.78 μM) and 12-fold more potent (and more selective) than **9**α towards the insect trehalase.

## 1. Introduction

**Figure 1:**
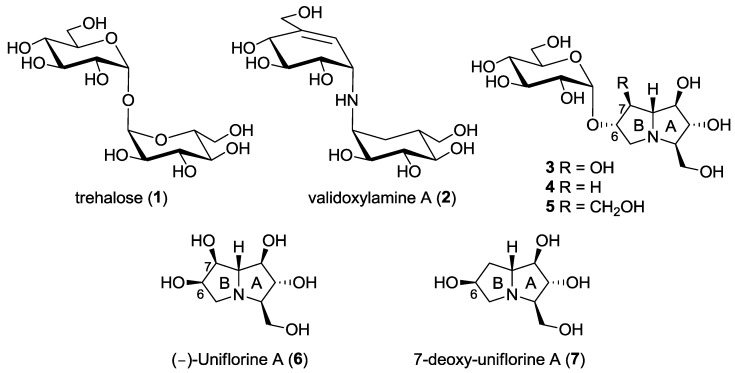
Trehalose (**1**), the natural substrate of the enzyme α-trehalase (EC 3.2.1.28), and some natural and non-natural trehalase inhibitors reported to date.

Trehalose (α-d-glucopyranosyl α-d-glucopyranoside, **1**, Figure 1) is a disaccharide with a multifunctional physiological role in various organisms (e.g., bacteria, fungi, invertebrates and higher plants) [1]. The role of trehalose in insects is of particular importance connected to its hydrolysis operated by the enzyme α-trehalase (EC 3.2.1.28). Indeed, α-trehalase is an inverting glycosidase [2] that promotes the conversion of trehalose into two molecules of glucose, which is vital for insect flight and essential for larvae resistance to stress factors. While trehalose is not present in mammalian cells, humans do possess the enzyme trehalase, only at intestinal level, probably to hydrolyze occasionally ingested trehalose. Indeed, intolerance to fungi has been correlated with the absence or deficit of trehalases in mammals [1]. For the important role of trehalose-derived glucose in larvae survival and development, trehalase inhibitors have been considered in recent years an interesting target for the identification of novel insecticides and fungicides. However, due to the presence of trehalase also in mammals, specificity towards insect trehalase is crucial for the development of drugs that are safe for plants and mammals, and possibly also for insects that are of benefit in nature [3].

Among the most powerful inhibitors of trehalases there are some natural pseudodisaccharides and analogs shown in Figure 1, such as validoxylamine (**2**), casuarine-6-*O*-d-glucoside **3** and its non-natural analogues **4** and **5**. The first investigated trehalase three-dimensional structure was the family 37 periplasmic enzyme from *E. coli* (Tre37A), which was solved in complex with **2** [4], with casuarine-6-*O*-d-glucoside **3** [5] and with non-natural analogue **5** [6]. Our findings showed that, with *E. coli* trehalase, casuarine-based inhibitors are placed within the primary catalytic site with the A ring of the pyrrolizidine nucleus that mimics the natural glucose configuration [5,6]. However, subtle changes at ring B (e.g., modification at C-7 as in compound **5**) were able to confer both potency and specificity in trehalase inhibition [6]. More interestingly, we later found that simpler pseudomonosaccharide inhibitors such as natural (-)-uniflorine A (**6**) and non-natural analogue 7-deoxy-uniflorine A (**7**) showed an excellent inhibitory profile, being completely selective towards the insect trehalase, although less potent in absolute value with respect to casuarine-6-*O*-d-glucoside derivatives **3**–**5** [7]. It is worth noting that compounds **6** and **7** bear the opposite configuration at C-6 in ring B with respect to the previously investigated casuarine-6-*O*-d-glucoside and analogues (compounds **3**–**5**).

**Figure 2:**
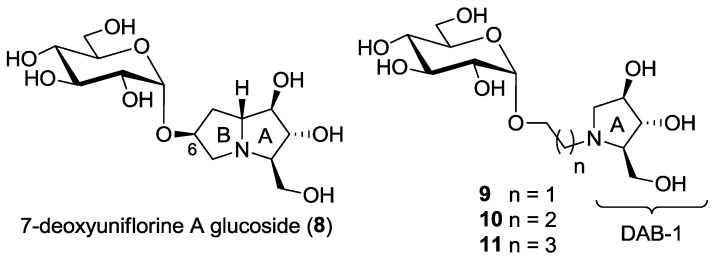
This work: synthesis of a new pseudodisaccharide mimetic **8** and of a series of more flexible disaccharide mimics **9**–**11** bearing the DAB-1 nucleus instead of the pyrrolizidine moiety.

Following our interest in the identification of new more potent and selective trehalase inhibitors, we envisaged that the synthesis of a pseudodisaccharide **8** (7-deoxyuniflorine A glucoside), analogue of disaccharide **4** but with an opposite configuration at C-6, would furnish a promising inhibitor (Figure 2). A straightforward approach to compound **8** is reported in this work.

In consideration of the high number of steps required for the synthesis of pseudodisaccharides bearing the pyrrolizidine nucleus such as compounds **3**–**5** and **8** itself, we also wanted to explore the insect trehalase activity of simpler pseudodisaccharide inhibitors **9**–**11**, that bear the glucose unit linked to the pyrrolidine DAB-1 instead of the pyrrolizidine nucleus. DAB-1 actually has the same structure of ring A of the pyrrolizidine nucleus (Figure 2), and we had previously verified that it is able to mimic glucose by imparting trehalase inhibition in pseudodisaccharide mimetics [8,9]. The aim of the present work was to link the DAB-1 moiety to the glucose moiety through a linker of variable length (2, 3 and 4 carbon atoms), in order to verify which was the more appropriate tether length (Figure 2) for trehalase inhibition.

**Scheme 1:**
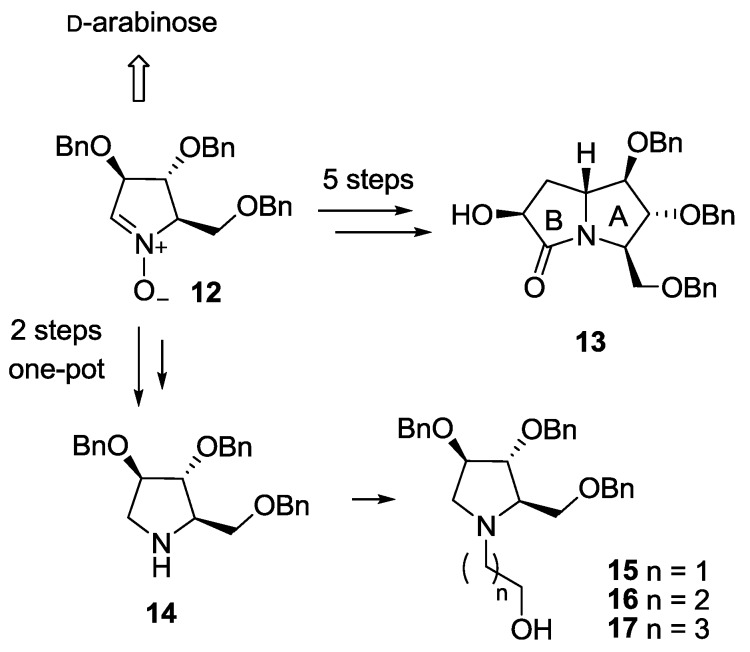
Synthetic steps to access pyrrolizidine lactam **13** and hydroxypyrrolidines **15**–**17** (through the key intermediate **14**) from nitrone **12**.

The synthesis of both pyrrolizidine-based (**8**) and pyrrolidine-based (**9**–**11**) pseudodisaccharides involved a common key intermediate: the enantiopure nitrone **12**, readily available from d-arabinose in 5 steps (Scheme 1) [10]. However, while on one hand 5 further steps are necessary to access the pyrrolizidine glucosyl acceptor (lactam **13**) [7], the hydroxypyrrolidine glucosyl acceptors **15**–**17** are in principle readily available from amine **14** [11,12]. Pyrrolidine **14**, recently synthesized by some of us in a one-pot 2-steps sequence from nitrone **12** (Scheme 1) [13], has the same stereochemical pattern of the hydroxyl groups of ring A in lactam **13**, which is responsible for the key interactions within the enzyme active site of the final compounds [5,6]. Therefore, compound **14** is in principle able to mimic the more complex pyrrolizidine skeleton of lactam **13**. We also reasoned that pyrrolidine **14** could be easily functionalized at the endocyclic nitrogen atom to afford the desired glucosyl acceptors **15**–**17**, allowing to probe different spatial lengths between the sugar and the iminosugar unit of the target pseudodisaccharides.

This work has therefore a dual aim: from one side to investigate the synthesis and biological activity of a new and differently configured pseudodisaccharide mimic (compound **8**), and from the other side to explore the possibility to obtain simpler and more flexible inhibitors (compounds **9**–**11**). The results in this direction are shown herein, and highlight the crucial role of the linker length in the design of more flexible inhibitors.

## 2. Results and Discussion

**Scheme 2:**
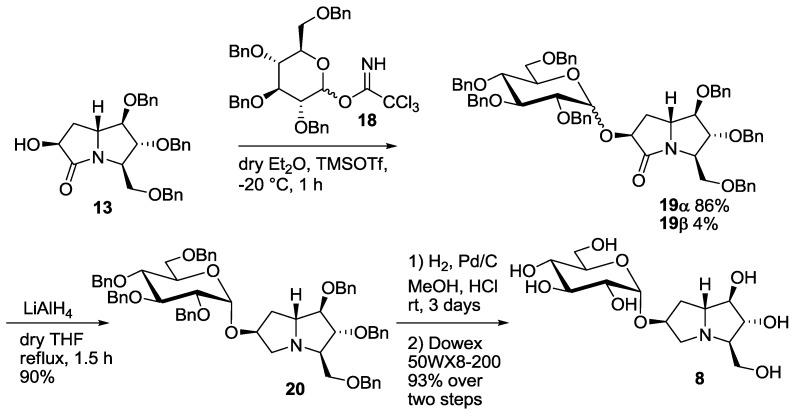
Synthesis of the pseudodisaccharide 7-deoxyuniflorine A glucoside (**8**).

The synthesis of the new disaccharide mimetic **8** started from lactam **13**, whose preparation was recently reported by some of us [7]. Compound **13** bears a free OH group at C-6, and was therefore employed as the acceptor in the glucosylation reaction with the benzyl protected glucopyranosyl trichloroacetimidate **18** (Scheme 2) [14]. The reaction, performed in diethyl ether at −20 °C, was highly selective in favor of the α-anomer, as reported before for the casuarine derivatives [5,6], and afforded the α glucoside **19**α as the major product (86% yield). Small amounts (4% yield) of the β-anomer **19**β were isolated and characterized. Reduction of the C=O double bond in **19**α with LiAlH_4_ in THF at reflux temperature afforded compound **20** in excellent yield (90%). Catalytic hydrogenation, followed by treatment with the ion exchange resin 50WX8-200 gave 7-deoxy-uniflorine A glucoside **8** in 93% yield over two steps (Scheme 2). The ^1^H-NMR spectrum of compound **8** showed a *dd* at 3.30 ppm for H-2 signal, with coupling constants of 9.8 and 3.5 Hz, respectively. This indicates an *axial-axial* relationship with H-3 and an *axial-equatorial* relationship with β H-1, and therefore confirms the α-configuration of the glucose moiety.

In order to reduce the overall number of synthetic steps necessary to access the glucosyl acceptor in the final glucosylation with trichloroacetimidate **18**, we also designed and prepared a series of pseudodisaccharide derivatives **9**–**11** (Scheme 1) containing a DAB-1 nucleus and a remaining d-glucose unit linked through a 2, 3 or 4-carbon atoms spacer.

**Scheme 3:**
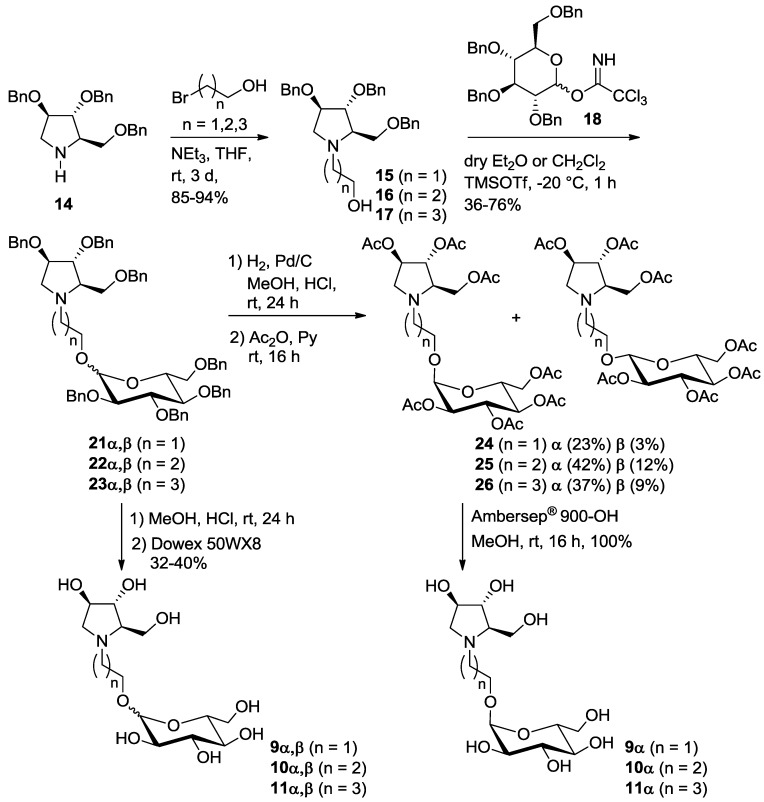
Synthesis of pseudodisaccharides **9–11α**, composed by glucose 1-α-linked to the pyrrolidine iminosugar DAB-1.

Pyrrolidine **14** was *N*-alkylated with an excess of 2-bromo-1-ethanol, 3-bromo-1-propanol and 4-bromo-1-butanol in THF, using Et_3_N as the base at room temperature, affording alcohols **15** (85% yield) [12], **16** (94% yield) and **17** (88% yield), with a free hydroxyl group at the end of the aliphatic chain, thus allowing selective glucosylation at this position (Scheme 3).

The reaction of alcohols **15**–**17** with trichloroacetimidate donor **18** [14] in dry diethyl ether in the presence of trimethylsilyl trifluoromethanesulfonate (TMSOTf) gave **21** (50% yield), **22** (36%) and **23** (66%) as α,β glucoside mixtures, impossible to be separated by flash column chromatography (Scheme 3). In all cases it was not possible to determine the α:β ratios by ^1^H-NMR analysis because the signals of the anomeric protons of the two anomers were covered by the CH_2_ benzyl groups signals. The glucosylation reactions were also performed in dry CH_2_Cl_2_ affording similar yields (**21**, 76%, **22**, 39% and **23**, 50%) of the α,β glucoside mixtures.

Catalytic hydrogenation of **21**–**23**α,β in acidic MeOH with Pd/C gave the corresponding mixture of deprotected α,β isomers **9**–**11**, as hydrochloride salts, that were passed onto an ion exchange resin (Dowex 50WX8-200) eluting successively with MeOH, H_2_O and 6% aqueous ammonia. The final elution with ammonia afforded pure glucosyl derivatives **9**–**11** as a mixture of α and β-anomers, in 32–40% yields (**9**α,β, α:β 2:1; **10α**,β, α:β 1:1; **11α**,β, α:β 1.2:1 as determined by ^1^H NMR analysis, see Supplementary Materials).

Due to the impossibility to separate the α and β isomers both with benzylated and free hydroxyl groups, we decided to directly react the crude obtained by catalytic hydrogenation of **9**–**11**α,β with an excess of acetic anhydride in pyridine affording the corresponding peracetylated compounds **24**–**26** as α,β mixtures. As expected, in all cases, the two isomers were separated by flash column chromatography, affording pure compounds **24**α, **25**α and **26**α in 23%, 42% and 37% yields over two steps and β-isomers (**24**β**, 25**β and **26**β in 3%, 12% and 9% yield) impure of some traces of the corresponding α-isomers (Scheme 3). The α-isomers **24**–**26**α were subsequently treated with strongly basic Ambersep 900 OH resin in MeOH, leading to pure polyhydroxylated α-pseudodisaccharides **9**–**11**α in quantitative yield.

Synthesized compounds **8**, **9**α, **10**α, **11**α and the α/β mixture of compounds **9**,**10** and **11** were tested for their inhibitory activity against insect (*C. riparius*) and porcine kidney trehalases and the results are shown in Table 1, together with the previously published data on compounds **4**, **6** and **7** [7].

As already mentioned in the introduction, compounds **6** and **7**, bearing the opposite configuration at C-6 with respect to the pyrrolizidine portion of compound **4**, showed a remarkable selectivity (higher than 5000) towards the insect trehalase with respect to the porcine enzyme. However, they were less active (one order of magnitude) than the pseudodisaccharide mimic **4** [7]. For this reason, we planned the synthesis of compound **8**, possessing both a pseudodisaccharide structure and the same configuration at the C-6 carbon atom of compounds **6** and **7**. The IC_50_ value, measured towards insect trehalase, appeared quite disappointing, since compound **8** was active only in the μM range. However, quite a good selectivity was still observed with respect to porcine trehalase (entry 4, Table 1). These results can be rationalized assuming that the active catalytic site of the *C. riparius* trehalase accommodates the pyrrolizidine portion of the compound, as it happens with recombinant Tre37A trehalase, [5,6]: in this case it appears evident that a pyrrolidizine with such configuration at C-6 (such as **8**) is not able to place the glucosyl moiety in a part of the enzyme cavity with favorable interactions.

**Figure 3:**
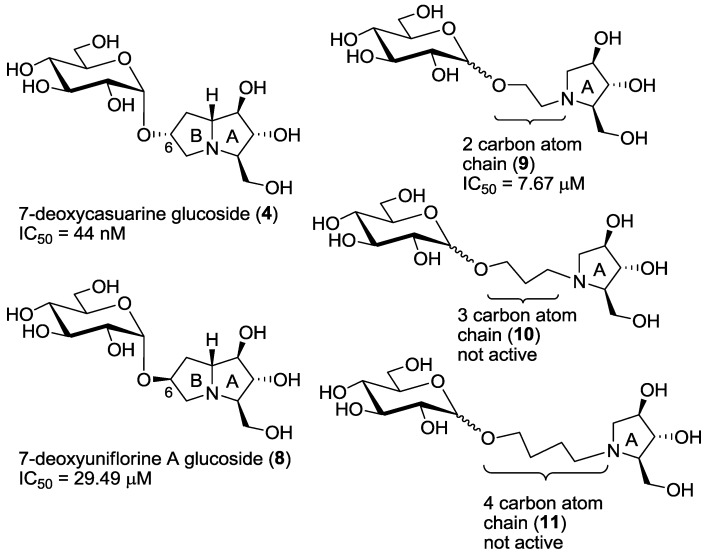
Compounds **4**, **8**, **9**, **10**, **11** and their IC_50_ values towards *C. riparius* trehalase.

Derivatives **9**–**11** were designed in order to simplify the overall synthesis of the inhibitors and the data, shown in Table 1, clearly demonstrate that only compounds **9** are able to maintain inhibitory properties towards *C. riparius* trehalase, while compounds with a longer linker chain (e.g., **10** and **11**) loose completely their inhibitory properties (entries 8–11). Collected data suggest that only the two-carbon chain linker of compounds **9** is able to mimic the pyrrolizidine moiety of compound **8** (see also Figure 3), while its higher flexibility probably allows a better placement of the inhibitor within the active cavity. This is a very good result, which demonstrates the crucial role played by the linker chain’s length joining the iminosugar and the glucosyl moiety. Considering that compounds **9** are more active than the pyrrolizidine-based pseudodisaccharide **8**, the advantage of using flexible pyrrolidine-based inhibitors was therefore demonstrated.

**Scheme 4:**
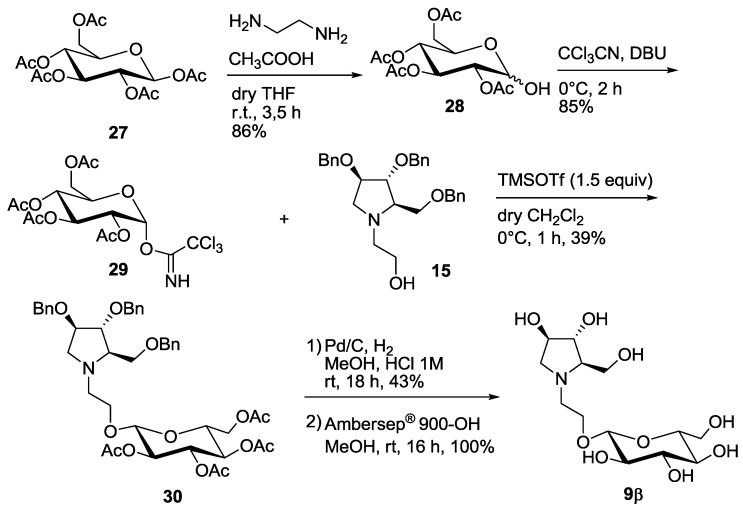
Synthesis of the pseudodisaccharide **9**β.

Interestingly, the **9**α,β mixture was more active than compound **9α** alone (entry 5 vs. entry 6, Table 1). Thus, we reasoned that the pure β-anomer might be even more active. In order to obtain a substantial amount of the β-isomer **9**β, we decided to change the protecting groups on the glycosyl donor by employing the *O*-acetyl protected 1-α-trichloroacetimidate **29** (Scheme 4), which would in principle favor the formation of β-pseudodisaccharide **30** in the following glycosylation reaction through the orthoester procedure (1,2-trans glycosylation) [15,16].

Selective deprotection of **27** with ethylendiamine in acetic acid following the procedure reported by Sun et al. [17] afforded in 86% yield compound **28**, that was treated with trichloroacetonitrile to obtain **29** in 85% yield. Glycosylation reaction with **15** at 0 °C in dry CH_2_Cl_2_ in the presence of TMSOTF (1.5 equiv) afforded 39% yield of the β-glucoside **30** as the major compound, although impure of a partially deacetylated isomer (See Supplementary Materials). The ^1^H-NMR spectrum of compound **30** showed a *dd* at 5.05 ppm for H-2′ signal (appearing as a pseudo *triplet*), with coupling constants of 9.6 and 9.8 Hz. This indicates an *axial-axial* relationship with both H-1′ and H-3′, and therefore confirms the β-configuration of the glucose moiety.

Deprotection of the benzyl groups by catalytic hydrogenation and of the acetyl groups by treatment with Ambersep 900-OH, allowed to isolate pure disaccharide **9**β in 43% yield over 2 steps (Scheme 4). To our delight and accordingly to our expectation, compound **9**β was 12-fold more active than its α-anomer towards *C. riparius* trehalase and was the most potent insect trehalase inhibitor of the pseudodisaccharide pyrrolidine series, with an IC_50_ in the low micromolar range (IC_50_ = 0.784 μM, entry 7, Table 1). Moreover, **9**β showed also a good 7-fold selectivity towards the insect trehalase vs. the porcine enzyme.

To evaluate the inhibitory pattern we performed kinetics measurements by varying the trehalose concentration, at two different concentrations of compounds **9**α and **9**β. Results showed a pure competitive inhibitory pattern. To determine the inhibition type, data were plotted according to the Lineweaver-Burk plot, replots were built and an inhibition constant (K_i_) was calculated equal to 2.56 ± 0.42 µM for **9**α and 0.40 ± 0.022 µM for **9**β (see supplementary materials).

## 3. Materials and Methods

### 3.1. General Experimental Procedures

All the starting reactants, solvents, and catalysts were commercially available unless otherwise stated. All reactions were carried out under magnetic stirring and monitored by TLC on 0.25 mm silica gel plates (Merck F254). Column chromatographies were carried out on Silica Gel 60 (32–63 μm) or on silica gel (230–400 mesh, Merck, Kenilworth, NJ, USA). Yields refer to spectroscopically and analytically pure compounds unless otherwise stated. ^1^H-NMR spectra were recorded on a Varian Mercury-400 or on a Varian INOVA 400 instruments (Agilent Technologies, Santa Clara, CA, USA) at 25 °C. ^13^C-NMR spectra were recorded on a Varian Gemini-200 spectrometer (Agilent Technologies, Santa Clara, CA, USA). Chemical shifts are reported relative to TMS (^1^H: *δ* = 0.00 ppm) and CDCl3 (^13^C: *δ* = 77.0 ppm). Integrals are in accordance with assignments, coupling constants are given in Hz. For detailed peak assignments 2D spectra were measured (COSY, HSQC, NOESY, and NOE as necessary). IR spectra were recorded with a BX FTIR Perkin-Elmer system spectrophotometer (Perkin-Elmer, Waltham, MA, USA). ESIMS spectra were recorded with a Thermo Scientific™ LCQ fleet ion trap mass spectrometer (Thermo Fisher Scientific, Waltham, MA, USA). Elemental analyses were performed with a Perkin-Elmer 2400 analyzer (Perkin-Elmer, Waltham, MA, USA). Optical rotation measurements were performed on a JASCO DIP-370 polarimeter (JASCO, Easton, MD, USA).

### 3.2. Synthesis and Purification of 7-Deoxyuniflorine A Glucoside *(**8**)*

#### 3.2.1. Synthesis of Compounds **18**

To a solution of 2,3,4,6-tetra-*O*-benzyl-d–glucopyranose (300 mg, 0.55 mmol) in 7 mL of dry CH_3_CN, trichloroacetonitrile (568 μL, 7.15 mmol) and K_2_CO_3_ (380 mg, 2.75 mmol) were added under nitrogen atmosphere and the mixture was stirred at room temperature for 4 h. When a TLC analysis (CH_2_Cl_2_/MeOH 30:1) showed the disappearance of the starting material (*R*_f_ = 0.06) and formation of a new product (*R*_f_ = 0.33), the mixture was filtered through Celite^®^ and the solvent was removed under reduced pressure affording a crude oil. The crude, obtained in a quantitative yield, was used directly for the next step without further purification.

#### 3.2.2. Synthesis of Compounds **19**

A solution of alcohol **13** [7] (100 mg, 0.21 mmol) and glucopyranosyl tricholoroacetimidate **18** (230 mg, 0.35 mmol) in 4.2 mL of dry Et_2_O was stirred for 10 min at room temperature under nitrogen atmosphere in the presence of 3 Å molecular sieves. After cooling to −20 °C and addition of trimethylsilyl trifluoromethanesulfonate (20 µL, 0.11 mmol), stirring was continued for 1 h; during this period the temperature was raised to room temperature. A TLC analysis (PE/EtOAc 1:1) showed the disappearance of the starting material (*R*_f_ = 0.22) and formation of two new products (*R*_f_ = 0.72 and 0.66). Then 2 mL of a saturated Na_2_CO_3_ solution were added, the mixture was extracted with Et_2_O (2 × 15 mL) and the organic layers were dried on Na_2_SO_4_. Filtration on cotton and evaporation under reduced pressure afforded the crude **19** that was purified by flash column chromatography on silica gel (PE/EtOAc from 3:1 to 1:1) to afford pure **19**β (*R*_f_ = 0.25 PE/EtOAc 3:1, 9 mg, 0.009 mmol, 4% yield) and **19**α (*R*_f_ = 0.18 PE/EtOAc 3:1, 179 mg, 0.18 mmol, 86% yield) as yellow oils.

*(1R,2R,3R,6S,7aR)-1,2-bis(benzyloxy)-3-[(benzyloxy)methyl]-5-oxohexahydro-1H-pyrrolizin-6-yl 2,3,4,6-tetra-O-benzyl-α-hexopyranoside.* **19**α: [α${]}_{D}^{18}$ = +18.3 (*c* = 0.06, CHCl_3_). ^1^H-NMR (400 MHz, CDCl_3_): *δ* = 7.38–7.11 (m, 35 H, H-Ar), 4.92–4.40 (m, 14 H, H-Bn), 4.83–4.80 (m, 1H, H-1), 4.33 (dd, *J* = 4.7, 4.1, 1H, H-1′), 4.25–4.23 (m, 1 H, H-6′), 4.08–4.02 (m, 3 H, H-7a′, H-5, H-3′), 3.95 (t, *J* = 9.4 Hz, 1H, H-3), 3.77 (dd, *J* = 10.8, 2.6 Hz, 1 H, Ha-8′), 3.72 (t, *J* = 9.7 Hz, 1 H, H-4), 3.68–3.65 (m, 1 H, Hb-8′), 3.63–3.56 (m, 4 H, H-2, H-2′, H-6), 2.23–2.18 (m, 1 H, Ha-7′), 1.95 (dt, *J* = 13.8, 6.7 Hz, 1 H, Hb-7′) ppm. ^13^C-NMR (50 MHz, CDCl_3_): 171.3 (s, C=O), 139.1–137.6 (s, 7 C, C-Ar), 128.6–127.6 (d, 35 C, C-Ar), 96.7 (d, C-1), 88.8 (d, C-2′), 87.2 (d, C-1′), 82.0 (d, C-3), 80.2 (d, C-2), 77.8 (d, C-6′), 77.2 (d, C-4), 76.5–72.4 (t, 7 C, C-Bn), 70.5 (d, C-3′), 69.2 (t, C-6), 68.3 (t, C-8), 62.0 (d, C-7a′), 58.9 (d, C-5), 33.3 (t, C-7′) ppm. MS (ESI): *m*/*z* (%) = 1018.75 (100) [M + Na]^+^. IR (CDCl_3_): 3088, 3066, 3032, 2915, 2867, 1700, 1602, 1496, 1453, 1363, 1098, 1071, 1028 cm^−1^. Elemental analysis (%) for C_63_H_65_NO_10_ (996.19): calcd. C 75.96, H 6.58, N 1.41; found C 75.92, H 6.55, N 1.43.

*(1R,2R,3R,6S,7aR)-1,2-bis(benzyloxy)-3-[(benzyloxy)methyl]-5-oxohexahydro-1H-pyrrolizin-6-yl 2,3,4,6-tetra-O-benzyl-β-hexopyranoside.* **19**β: [α${]}_{D}^{19}$ = −40.0 (*c* = 0.04, CHCl_3_). ^1^H-NMR (400 MHz, CDCl_3_): *δ* = 7.36–7.12 (m, 35 H, H-Ar), 4.91–4.49 (m, 14 H, H-Bn), 4.82–4.79 (m, 1 HH-1), 4.46–4.44 (m, 1 H, H-6′), 4.32 (dd, *J* = 4.7, 3.8, 1H, H-1′), 4.10–4.04 (m, 2 H, H-7a′ and H-5), 3.70–3.59 (m, 5 H, H-2′, H-3, H-4 and H-8), 3.50–3.39 (m, 4 H, H-3′, H-2 and H-6), 2.46–2.41 (m, 1 H, Ha-7′), 2,04 (dt, *J* = 14.7, 7.0 Hz, 1 H, Hb-7′) ppm. ^13^C-NMR (100 MHz, CDCl_3_): 171.5 (s, C=O), 138.6–137.5 (s, 7 C, C-Ar), 128.5–127.5 (d, 35 C, C-Ar), 102.2 (d, C-1), 88.8 (d, C-2′), 87.3 (d, C-1′), 84.5 (d, C-4), 81.8 (d, C-2), 78.6 (d, C-6′), 77.3 (d, C-3), 75.7–72.2 (t, 7 C, C-Bn), 74.3 (d, C-3′), 69.0 (t, C-6), 68.7 (t, C-8), 62.4 (d, C-7a′), 58.5 (d, C-5), 34.8 (t, C-7′) ppm. MS (ESI): *m*/*z* (%) = 1018.58 (100) [M + Na]^+^. IR (CDCl_3_): 3089, 3066, 3032, 2905, 2868, 1697, 1602, 1496, 1454, 1362, 1095, 1069, 1028 cm^−1^. Elemental analysis (%) for C_63_H_65_NO_10_ (996.19): calcd. C 75.96, H 6.58, N 1.41; found C 75.94, H 6.57, N 1.41.

#### 3.2.3. Synthesis of Compound **20**

A solution of **19**α (118 mg, 0.12 mmol) in 1.2 mL of dry THF was stirred under nitrogen atmosphere at 0 °C and LiAlH_4_ (1 M solution in THF, 0.56 mL, 0.56 mmol) was added drop wise. The mixture was then heated at reflux temperature for 1.5 h, until a TLC analysis (PE/EtOAc 2:1) showed the disappearance of the starting material (*R*_f_ = 0.57) and the formation of a new product (*R*_f_ = 0.75). The reaction was then quenched at 0 °C with a saturated aqueous solution of Na_2_SO_4_ (5 mL). After extraction with EtOAc (3 × 15 mL), the organic layers were dried on Na_2_SO_4_ and concentrated at reduced pressure affording the crude that was purified by flash column chromatography on silica gel (PE/EtOAc 4:1) to afford **20** pure (*R*_f_ = 0.25, 108 mg, 0.11 mmol, 90% yield) as a waxy white solid.

*(1R,2R,3R,6S,7aR)-1,2-bis(benzyloxy)-3-[(benzyloxy)methyl]hexahydro-1H-pyrrolizin-6-yl 2,3,4,6-tetra-O-benzyl-α-hexopyranoside* **20**: [α${]}_{D}^{22}$ = +50.6 (*c* = 0.17, CHCl_3_). ^1^H-NMR (400 MHz, CDCl_3_): *δ* = 7.36–7.12 (m, 35 H, H-Ar), 4.98–4.39 (m, 14 H, H-Bn), 4.84–4.80 (m, 1H, H-1), 4.36–4.30 (m, 1H, H-6), 4.03 (t, *J* = 5.3 Hz, 1 H, H-2′), 3.96–3.90 (m, 1 H, H-1′), 3.84–3.63 (m, 5 H, H-4, H-5, H-3, H-7a′, Ha-6), 3.59–3.54 (m, 3 H, 2-H, Hb-6, Ha-8), 3.49 (dd, *J* = 9.4, 5.9 Hz, 1 H, Hb-8), 3.16 (dd, *J* = 11.7, 3.5 Hz, 1 H, Ha- 5′), 3.01 (dd, *J* = 12.3, 5.3 Hz, 1 H, Hb-5′), 2.94 (q, *J* = 5.9 Hz, 1 H, H-3′), 2.09–2.05 (m, 1 H, Ha-7′), 1.82 (ddd, *J* = 13.5, 7.6, 5.9 Hz, 1 H, Hb-7′) ppm. ^13^C-NMR (50 MHz, CDCl_3_): 139.1–138.1 (s, 7 C, C-Ar), 128.4–127.0 (d, 35 C, C-Ar), 96.5 (d, C-1), 88.5 (d, C-3), 86.1 (d, C-2′), 82.0 (d, C-1′), 80.3 (d, C-2), 78.4 (t, C-6′), 77.9 (d, C-4), 75.7–71.8 (t, 8 C, C-Bn and C-8), 70.6 (d, C-5), 69.4 (d, C-3′), 68.6 (t, C-6), 66.4 (d, C-7a′), 60.6 (t, C-5′), 37.0 (t, C-7′) ppm. MS (ESI): *m*/*z* (%) = 982.50 (100) [M + H]^+^. IR (CDCl_3_): 3089, 3067, 3032, 2915, 2867, 1605, 1496, 1454, 1363, 1144, 1070, 1028 cm^−1^. Elemental analysis (%) for C_63_H_67_NO_9_ (982.21): calcd. C 77.04, H 6.88, N 1.43; found C 77.01, H 6.87, N 1.41.

#### 3.2.4. Synthesis of Compound **8**

To a solution of compound **20** (153 mg, 0.16 mmol) in 15 mL of methanol, 10% Pd/C (77 mg, 50 % weight) and two drops of 37% HCl were added under nitrogen atmosphere, then the mixture was stirred under hydrogen atmosphere at room temp for three days. At completion of the reaction, the mixture was filtered through Celite^®^ and the solvent was removed under reduced pressure affording a crude oil. Free amine **8** (53 mg, 0.15 mmol, 93% yield) was obtained by purification with ion exchange resin Dowex 50WX8-200, eluting successively with MeOH, H_2_O and 6% NH_4_OH.

*(1R,2R,3R,6S,7aR)-1,2-dihydroxy-3-(hydroxymethyl)hexahydro-1H-pyrrolizin-6-yl-α-hexopyranoside* **8**: [α${]}_{D}^{22}$ = +102.3 (*c* = 0.39, H_2_O). ^1^H-NMR (400 MHz, H_2_O): *δ* = 4.78 (d, *J* = 3.9 Hz, 1H, H-1), 4.36–4.30 (m, 1H, H-6′), 3.68–3.63 (m, 2 H, H-1′, H-2′), 3.63–3.44 (m, 4 H, H-4, H-5, H-6), 3.57 (dd, *J* = 11.7, 3.9 Hz, 1H, Ha-8), 3.41 (dd, *J* = 11.7, 6.9 Hz, 1 H, Hb-8), 3.30 (dd, *J* = 9.8, 3.5 Hz, 1 H, H-2), 3.18–3.12 (m, 1 H, H-7a′), 3.16 (t, *J* = 9.7 Hz, 1 H, H-3), 3.01–2.98 (m, 1 H, Ha-5′), 2.76 (dd, *J* = 11.7, 3.9 Hz, 1 H, Hb-5′), 2.63–2.56 (m, 1 H, H-3′), 2.13–2.07 (m, 1 H, Ha-7′), 1.71 (ddd, *J* = 13.2, 8.3, 4.9, 1 H, Hb-7′) ppm. ^13^C-NMR (100 MHz, H_2_O): 96.0 (d, C-1), 80.3 (d, C-1′), 78.0 (d, C-6′), 77.6 (d, C-2′), 72.9 (d, C-4), 71.9 (d, C-5), 71.1 (d, C-2), 70.1 (d, C-3′), 69.5 (d, C-3), 64.9 (d, C-7a′), 62.5 (t, C-8), 60.6 (t, C-6), 60.5 (t, C-5′), 34.2 (t, C-7′) ppm. MS (ESI): *m*/*z* (%) = 374.25 (100) [M + Na]^+^. Elemental analysis (%) for C_14_H_25_NO_9_ (351.15): calcd. C 47.86, H 7.17, N 3.99; found C 47.83, H 7.16, N 3.98.

### 3.3. Synthesis and Purification of Pyrrolidine-based Pseudodisacharides ***9**α*, ***10**α* and ***11**α*

#### 3.3.1. Synthesis of Compound **15**

To a solution of **14** [13] (82 mg, 0.20 mmol) in 3 mL of THF, NEt_3_ (141 μL, 1.00 mmol) and 2-bromo-1-etanol (85 μL, 1.22 mmol) were added. The reaction mixture was stirred at room temperature for 3 days until a TLC analysis (CH_2_Cl_2_:MeOH 30:1) showed the disappearance of the starting material (R_f_ = 0.43) and the formation of a new product (R_f_ = 0.81). After evaporation under reduced pressure, the crude was purified by FCC (AcOEt:PE 1:1) affording pure **15** (R_f_ = 0.30, 75 mg, 0.17 mmol, 85% yield) as a yellow oil.

*2-{(2R,3R,4R)-3,4-bis(benzyloxy)-2-[(benzyloxy)methyl]-1-[(2-hydroxy)etyl]-1H-pyrrolidine* **15** [12]: [α${]}_{D}^{25}$ = −20.3 (*c* = 0.92 in CHCl_3_); ^1^H-NMR (400 MHz, CDCl_3_): *δ* = 7.35–7.26 (m, 15H, H-Ar), 4.55–4.42 (m, 6H, H-Bn), 3.99–3.97 (m, 1H, H-4), 3.89 (dd, *J* = 3.6, 1.2 Hz, 1H, H-3), 3.64–3.48 (m, 4H, H-7, H-8), 3.25 (d, *J* = 10.4 Hz, 1H, Ha-5), 3.06 (ddd, *J* = 12.9, 9.1, 4.7 Hz, 1H, Ha-6), 2.88 (dd, *J* = 9.6, 5.6 Hz, 1H, H-2), 2.67 (dd, *J* = 10.4, 5.2 Hz, 1H, Hb-5), 2.58 (dt, *J* = 12.6, 3.8 Hz, 1H, Hb-6).

#### 3.3.2. Synthesis of Compound **16**

To a solution of **14** [13] (132 mg, 0.33 mmol) in 6 mL of THF, NEt_3_ (230 μL, 1.65 mmol) and 3-bromo-1-propanol (179 μL, 1.98 mmol) were added. The reaction mixture was stirred at room temperature for 3 days until a TLC analysis (CH_2_Cl_2_:MeOH 10:1) showed the disappearance of the starting material (R_f_ = 0.51) and the formation of a new product (R_f_ = 0.85). After evaporation under reduced pressure, the crude was purified by FCC (AcOEt:PE 1:1) affording pure **16** (R_f_ = 0.30, 143 mg, 0.31 mmol, 94% yield) as a yellow oil.

*(2R,3R,4R)-3,4-bis(benzyloxy)-2-[(benzyloxy)methyl]-1-[(3-hydroxy)propyl]-1H-pyrrolidine ***16**: [α${]}_{D}^{24}$ = −39.1 (*c* = 0.47 in CHCl_3_); ^1^H-NMR (400 MHz, CDCl_3_): *δ* = 7.34–7.24 (m, 15H, H-Ar), 4.56–4.48 (m, 6H, H-Bn), 3.93 (d, *J* = 4.8 Hz, 1H, H-4), 3.82–3.77 (m, 3H, H-9 and H-3), 3.63 (dd, *J* = 9.7, 5.8 Hz, 1H, Ha-6), 3.55 (dd, *J* = 9.8, 6.4 Hz, 1H, Hb-6), 3.45 (d, *J* = 10.8 Hz, 1H, Ha-5), 3.14 (td, *J* = 11.7, 3.9 Hz, 1H, Ha-7), 2.73 (q, *J* = 5.2 Hz, 1H, H-2), 2.64 (dt, *J* = 12.2, 3.9 Hz, 1H, Hb-7), 2.50 (dd, *J* = 10.8, 4.7 Hz, 1H, Hb-5), 1.94–1.82 (m, 1H, Ha-8), 1.56–1.49 (m, 1H, Hb-8); ^13^C-NMR (50 MHz, CDCl_3_): *δ* = 138.3, 138.2, 138.1 (s, 3C, C-Ar), 128.3–127.5 (d, 15C, C-Ar), 85.3 (d, C-3), 81.4 (d, C-4), 73.3, 71.4, 71.2, 70.9 (t, 4C, C-Bn and C-6), 69.7 (d, C-2), 64.0 (t, C-9), 56.7 (t, C-5), 55.2 (t, C-7), 29.3 (t, C-8); IR (CDCl_3_): ν = 3312, 3066, 3032, 2923, 2861, 1496, 1453, 1366, 1333, 1282, 1208, 1100, 1071, 1028 cm^−1^; MS (ESI): *m*/*z* calcd (%) for C_29_H_35_NO_4_ + Na^+^ 484.26 [M + Na]^+^; found: 484.33; elemental analysis calcd (%) for C_29_H_35_NO_4_ (461.59): C 75.46, H 7.64, N 3.03; found: 75.43, H 7.62, N 3.02.

#### 3.3.3. Synthesis of Compound **17**

To a solution of **14** [13] (100 mg, 0.25 mmol) in 1 mL of THF, NEt_3_ (174 μL, 1.25 mmol) and 4-bromo-1-butanol (158 μL, 1.5 mmol) were added. The reaction mixture was stirred at room temperature for 3 days until a TLC analysis (CH_2_Cl_2_:MeOH 10:1) showed the disappearance of the starting material (*R*_f_ = 0.51) and the formation of a new product (*R*_f_ = 0.65). After evaporation under reduced pressure, the crude was purified by FCC (AcOEt:PE 2:1) affording pure **17** (*R*_f_ = 0.35, 105 mg, 0.22 mmol, 88% yield) as a yellow oil.

*(2R,3R,4R)-3,4-bis(benzyloxy)-2-[(benzyloxy)methyl]-1-[(4-hydroxy)butyl]-1H-pyrrolidine* **17***:* [α${]}_{D}^{25}$ = −26.2 (*c* = 0.60 in CHCl_3_); ^1^H-NMR (400 MHz, CDCl_3_): *δ* = 7.34–7.24 (m, 15H, H-Ar), 4.56–4.39 (m, 6H, H-Bn), 3.94 (d, *J* = 4.8 Hz, 1H, H-4), 3.86 (d, *J* = 3.9 Hz, 1H, H-3), 3.67–3.63 (m, 2H, Ha-10 and Ha-6), 3.57–3.51 (m, 2H, Hb-10 and Hb-6), 3.30 (d, *J* = 10.8 Hz, 1H, Ha-5), 2.94–2.87 (m, 1H, Ha-7), 2.81–2.77 (m, 1H, H-2), 2.54 (dd, *J* = 10.7, 4.8 Hz, 1H, Hb-5), 2.46 (dt, *J* = 11.7, 4.4 Hz, 1H, Hb-7), 1.76–1.66 (m, 2H, Ha-8 and Ha-9), 1.61–1.51 (m, 2H, Hb-8 and Hb-9); ^13^C-NMR (50 MHz, CDCl_3_): *δ* = 138.4, 138.3, 138.2 (s, 3C, C-Ar), 128.2–127.5 (d, 15C, C-Ar), 85.4 (d, C-3), 81.6 (d, C-4), 73.2 (t, C-Bn), 71.4, 71.1, 71.0 (t, 3C, C-Bn and C-6), 69.7 (d, C-2), 62.6 (t, C-10), 56.9 (t, C-5), 56.0 (t, C-7), 31.9, 26.0 (t, 2C, C-8 and C-9); IR (CDCl_3_): ν = 3392, 3066, 3032, 2925, 2864, 1496, 1453, 1365, 1206, 1100 cm^−1^; MS (ESI): *m*/*z* calcd (%) for C_30_H_37_NO_4_ + H^+^ 476.27 [M + H]^+^; found: 476.60; elemental analysis calcd (%) for C_30_H_37_NO_4_ (475.62): C 75.76, H 7.84, N 2.94; found: C 75.74, H 7.82, N 2.93.

#### 3.3.4. Synthesis of Compounds **21­23**α/β

*General procedure for the glycosylation reaction:* A solution of glucopyranosyl tricholoroacetimidate **18** (1.65 equiv.) and alcohol (1 equiv.) in dry diethyl ether or dry CH_2_Cl_2_, was stirred for 10 min at room temperature under nitrogen atmosphere in the presence of 3 Å molecular sieves. After cooling to −20 °C trimethylsilyl trifluoromethanesulfonate (1.5 equiv.) was added and the mixture was stirred for 1 h; letting the temperature to rise. The mixture was washed with a saturated Na_2_CO_3_ solution, dried over Na_2_SO_4_, filtered and concentrated to dryness. The crude was purified by flash column chromatography on silica gel to afford the pure glycosylated compound as α,β mixture.

**21**α,β. Obtained as a brown oil in 50% (Et_2_O), 76% (CH_2_Cl_2_), 23% (CH_3_CN) yields on 0.22–0.34 mmol of alcohol **15**. *R*_f_ = 0.40 (EP:AcOEt 4:1); ^1^H-NMR (400 MHz, CDCl_3_): *δ* = 7.34–7.05 (m, 70H, H-Ar), 4.98–4.26 (m, 30H, H-Bn and OC*H*O), 3.97 (t, *J* = 9.3 Hz, 2H), 3.89–3.84 (m, 4H), 3.78–3.42 (m, 18H), 3.23–3.12 (m, 4H), 2.82–2.78 (m, 2H), 2.71–2.63 (m, 4H).

**22**α,β. Obtained as a brown oil in 36% (Et_2_O), 39% (CH_2_Cl_2_) yields on 0.27-0.31 mmol of alcohol **16**. *R*_f_ = 0.42 (EP:AcOEt 4:1); ^1^H-NMR (400 MHz, CDCl_3_): *δ* = 7.39–7.15 (m, 70H, H-Ar), 5.02–4.41 (m, 30H, H-Bn and OC*H*O), 4.03–3.99 (m, 2H), 3.95–3.91 (m, 4H), 3.81–3.44 (m, 18H), 3.24–3.21 (m, 2H), 3.05–2.93 (m, 2H), 2.79–2.73 (m, 2H), 2.63–2.57 (m, 2H), 2.51–2.45 (m, 2H), 1.98–1.84 (m, 4H).

**23**α,β. Obtained as a brown oil in 66% (Et_2_O), 50% (CH_2_Cl_2_) yields on 0.21–0.33 mmol of alcohol **17**. *R*_f_ = 0.45 (EP:AcOEt 4:1); ^1^H-NMR (400 MHz, CDCl_3_): *δ* = 7.30–7.05 (m, 70H, H-Ar), 4.92–4.30 (m, 30H, H-Bn and OC*H*O), 3.91 (t, *J* = 9.3 Hz, 2H), 3.83–3.79 (m, 4H), 3.70–3.31 (m, 18H), 3.13–3.11 (m, 2H), 2.81–2.74 (m, 2H), 2.65–2.62 (m, 2H), 2.45 (dd, *J* = 10.3, 4.9 Hz, 2H), 2.30–2.24 (m, 2H), 1.56–1.43 (m, 8H).

#### 3.3.5. Synthesis of Peracetylated Compounds **24**–**26**

*General procedure for the synthesis of peracetylated derivatives:* To a solution of the α,β mixture of benzylated derivatives **21**–**23** (1 equiv.) in 10–20 mL of methanol, 10% Pd/C (50% *w*/*w*) and 37% HCl were added under nitrogen atmosphere, then the mixture was stirred under hydrogen atmosphere at room temperature for 24 h. After that an ^1^H-NMR analysis showed the disappearance of the starting material, the mixture was filtered through Celite^®^ and the solvent was removed under reduced pressure. The crude yellow oil was dissolved in pyridine (2–6 mL) and acetic anhydride (1–2 mL) was added. The solution was stirred at room temperature overnight. Then, after concentration under reduced pressure, the α- and β- isomers were separated by flash column chromatography on silica gel affording pure peracetylated **24**–**26**α (23–42% yields over two steps) and **24**–**26β** (3–12% yields over two steps).

*Compound* **24**α. Obtained as a yellow oil in 23% yield starting from 0.26 mmol of **21**α,β. *R*_f_ = 0.32 (Et_2_O:CH_2_Cl_2_ 2:1); [α${]}_{D}^{22}$ = +61.3 (*c* = 0.39 in CHCl_3_); ^1^H-NMR (400 MHz, CDCl_3_): *δ* = 5.42 (t, *J* = 9.8 Hz, 1H, H-3′), 5.09 (d, *J* = 3.9 Hz, 1H, H-1′), 5.06–5.01 (m, 3H, H-3, H-4 and H-4′), 4.85 (dd, *J* = 10.3, 3.9 Hz, 1H, H-2′), 4.24 (dd, *J* = 12.2, 4.4 Hz, 1H, Ha-6′), 4.20 (dd, *J* = 11.2, 5.9 Hz, 1H, Ha-6), 4.10 (dd, *J* = 11.2, 4.9 Hz, 1H, Hb-6), 4.07 (dd, *J* = 12.2, 1.9 Hz, 1H, Hb-6′), 3.97 (ddd, *J* = 10.3, 4.4, 2.0 Hz, 1H, H-5′), 3.74 (dt, *J* = 11.3, 5.6 Hz, 1H, Ha-8), 3.57 (dt, *J* = 11.3, 5.9 Hz, 1H, Hb-8), 3.18 (d, *J* = 11.2 Hz, 1H, Ha-5), 3.03 (dt, *J* = 12.7, 6.4 Hz, 1H, Ha-7), 2.89 (dd, *J* = 11.2, 5.3 Hz, 1H, Hb-5), 2.82 (q, *J* = 4.9 Hz, 1H, H-2), 2.68 (dt, *J* = 12.7, 5.9 Hz, 1H, Hb-7), 2.07 (s, 3H, OAc), 2.06–2.05 (m, 12H, OAc), 2.00 (s, 3H, OAc), 1.99 (s, 3H, OAc); ^13^C-NMR (50 MHz, CDCl_3_): *δ* = 170.6, 170.4, 170.1, 170.0, 169.9, 169.5, 169.4 (s, 7C, C=O), 95.7 (d, C-1′), 78.7 (d, C-4), 76.3 (d, C-3), 70.8 (d, C-2′), 70.1 (d, C-3′), 68.7 (d, C-4′), 67.8 (d, C-2), 67.5 (d, C-5′), 66.6 (t, C-8), 63.2 (t, C-6), 62.0 (t, C-6′), 58.0 (t, C-5), 53.2 (t, C-7), 20.8, 20.7, 20.6, 20.5 (q, 7C, CH_3_); IR (CDCl_3_): ν = 2958, 2827, 2259, 1743, 1454, 1369, 1230, 1036 cm^−1^; MS (ESI): *m*/*z* calcd (%) for C_27_H_39_NO_16_ + Na^+^ 656.23 [M + Na]^+^; found: 656.33; elemental analysis calcd (%) for C_27_H_39_NO_16_ (633.60): C 51.18, H 6.20, N 2.21; found: C 51.15, H 6.18, N 2.20. *Compound* **24**β**.** Obtained as a yellow oil in 3% yield starting from 0.26 mmol of **21**α,β. *R*_f_ = 0.23 (Et_2_O:CH_2_Cl_2_ 2:1); [α${]}_{D}^{22}$ = −4.3 (*c* = 0.47 in CHCl_3_); ^1^H-NMR (400 MHz, CDCl_3_): *δ* = 5.19 (t, *J* = 9.3 Hz, 1H, H-3′), 5.10-5.02 (m, 3H, H-3, H-4, H-4′), 4.98 (dd, *J* = 9.2, 8.3 Hz, 1H, H-2′), 4.52 (d, *J* = 8.3 Hz, 1H, H-1′), 4.25 (dd, *J* = 12.2, 4.4 Hz, 1H, Ha-6′), 4.22 (dd, *J* = 11.2, 5.8 Hz, 1H, Ha-6), 4.14 (dd, *J* = 12.2, 2.4 Hz, 1H, Hb-6′), 4.09 (dd, *J* = 11.2, 4.4 Hz, 1H, Hb-6), 3.97 (dt, *J* = 9.8, 4.9 Hz, 1H, Ha-8), 3.68 (ddd, *J* = 9.7, 4.9, 2.4 Hz, 1H, H-5′), 3.61 (ddd, *J* = 10.2, 7.3, 4.4 Hz, 1H, Hb-8), 3.12 (d, *J* = 11.7 Hz, 1H, Ha-5), 3.00 (dt, *J* = 13.2, 4.9 Hz, 1H, Ha-7), 2.84 (dd, *J* = 11.7, 5.4 Hz, 1H, Hb-5), 2.80–2.76 (m, 1H, H-2), 2.66 (ddd, *J* = 13.2, 7.8, 4.4 Hz, 1H, Hb-7), 2.08 (s, 3H, OAc), 2.07 (m, 6H, OAc), 2.06 (s, 3H, OAc), 2.03 (s, 3H, OAc), 2.01 (s, 3H, OAc), 1.99 (s, 3H, OAc); ^13^C-NMR (100 MHz, CDCl_3_): *δ* = 170.8, 170.6, 170.2, 170.1, 169.5, 169.4, 169.2 (s, 7C, C=O), 100.5 (d, C-1′), 78.5 (d, C-4), 76.5 (d, C-3), 72.8 (d, C-3′), 71.9 (d, C-5′), 71.2 (d, C-2′), 68.8 (t, C-8), 68.4 (d, C-4′), 68.0 (d, C-2), 62.9 (t, C-6), 61.9 (t, C-6′), 58.3 (t, C-5), 53.3 (t, C-7), 21.0, 20.9, 20.8, 20.7, 20.6 (q, 7C, CH_3_); IR (CDCl_3_): ν = 2958, 2827, 2259, 1747, 1454, 1369, 1225, 1036 cm^−1^; MS (ESI): *m*/*z* calcd (%) for C_27_H_39_NO_16_ + Na^+^ 656.23 [M + Na]^+^; found: 656.42; elemental analysis calcd (%) for C_27_H_39_NO_16_ (633.60): C 51.18, H 6.20, N 2.21; found: C 51.15, H 6.18, N 2.19.

*Compound* **25**α. Obtained as a yellow oil in 42% yield starting from 0.12 mmol of **22**α,β. *R*_f_ = 0.34 (Et_2_O:CH_2_Cl_2_ 3:1); [α${]}_{D}^{23}$ = +48.6 (*c* = 0.42 in CHCl_3_); ^1^H-NMR (400 MHz, CDCl_3_): *δ* = 5.44 (t, *J* = 9.8 Hz, 1H, H-3′), 5.06–5.01 (m, 4H, H-3, H-4, H-1′ and H-4′), 4.86 (dd, *J* = 10.2, 3.4 Hz, 1H, H-2′), 4.23 (dd, *J* = 12.2, 4.4 Hz, 1H, Ha-6′), 4.15 (dd, *J* = 11.2, 6.3 Hz, 1H, Ha-6), 4.10 (dd, *J* = 11.2, 4.9 Hz, 1H, Hb-6), 4.07 (dd, *J* = 12.2, 2.4 Hz, 1H, Hb-6′), 3.97 (ddd, *J* = 10.3, 4.4, 2.4 Hz, 1H, H-5′), 3.71 (dt, *J* = 10.3, 6.4 Hz, 1H, Ha-9), 3.44 (dt, *J* = 10.3, 6.6 Hz, 1H, Hb-9), 3.12 (d, *J* = 11.2 Hz, 1H, Ha-5), 2.87 (dt, *J* = 12.2, 7.9 Hz, 1H, Ha-7), 2.76 (dd, *J* = 11.2, 4.9 Hz, 1H, Hb-5), 2.75–2.71 (m, 1H, H-2), 2.45 (dt, *J* = 12.2, 6.4 Hz, 1H, Hb-7), 2.08 (m, 6H, OAc), 2.06 (m, 6H, OAc), 2.04 (s, 3H, OAc), 2.01 (s, 3H, OAc), 1.99 (s, 3H, OAc), 1.77 (quint, *J* = 6.9 Hz, 2H, H-8); ^13^C-NMR (100 MHz, CDCl_3_): *δ* = 170.6, 170.4, 170.3, 170.2, 170.1, 169.9, 169.4 (s, 7C, C=O), 95.8 (d, C-1′), 78.9 (d, C-4), 76.3 (d, C-3), 70.9 (d, C-2′), 70.3 (d, C-3′), 68.8 (d, C-4′), 67.8 (d, C-2), 67.3 (d, C-5′), 66.3 (t, C-9), 63.2 (t, C-6), 62.0 (t, C-6′), 57.4 (t, C-5), 51.2 (t, C-7), 28.0 (t, C-8), 20.8, 20.7, 20.6, 20.5 (q, 7C, CH_3_); IR (CDCl_3_): ν = 2952, 2872, 2261, 1743, 1455, 1374, 1234, 1039 cm^−1^; MS (ESI): **m*/*z** calcd (%) for C_28_H_41_NO_16_ + Na^+^ 670.24 [M + Na]^+^; found: 669.72; elemental analysis calcd (%) for C_28_H_41_NO_16_ (647.62): C 51.93, H 6.38, N 2.16; found: C 51.92, H 6.29, N 2.15. *Compound* **25**β**.** Obtained as a yellow oil in 12% yield starting from 0.12 mmol of **22**α**,**β. *R*_f_ = 0.26 (Et_2_O:CH_2_Cl_2_ 3:1); [α${]}_{D}^{21}$ = −24.2 (*c* = 0.12 in CHCl_3_); ^1^H-NMR (400 MHz, CDCl_3_): *δ* = 5.19 (t, *J* = 9.7 Hz, 1H, H-3′), 5.07 (t, *J* = 9.7 Hz, 1H, H-4′), 5.07–5.03 (m, 2H, H-3 and H-4), 4.96 (dd, *J* = 9.7, 7.8 Hz, 1H, H-2′), 4.48 (d, *J* = 7.8 Hz, 1H, H-1′), 4.25 (dd, *J* = 12.2, 4.9 Hz, 1H, Ha-6′), 4.15–4.12 (m, 2H, H-6), 4.13 (dd, *J* = 12.2, 2.4 Hz, 1H, Hb-6′), 3.87 (dt, *J* = 9.4, 6.4 Hz, 1H, Ha-9), 3.68 (ddd, *J* = 10.2, 4.4, 2.4 Hz, 1H, H-5′), 3.53 (dt, *J* = 9.7, 6.4 Hz, 1H, Hb-9), 3.11 (d, *J* = 10.7 Hz, 1H, Ha-5), 2.83 (dt, *J* = 12.2, 7.8 Hz, 1H, Ha-7), 2.76–2.72 (m, 1H, H-2), 2.75 (dd, *J* = 11.2, 4.8 Hz, 1H, Hb-5), 2.43 (dt, *J* = 12.2, 6.4 Hz, 1H, Hb-7), 2.08 (m, 6H, OAc), 2.07 (s, 3H, OAc), 2.06 (s, 3H, OAc), 2.04 (s, 3H, OAc), 2.01 (s, 3H, OAc), 1.99 (s, 3H, OAc), 1.74 (quint, *J* = 6.8 Hz, 2H, H-8); ^13^C-NMR (100 MHz, CDCl_3_): *δ* = 170.8, 170.6, 170.2, 170.0, 169.5, 169.3, 169.1 (s, 7C, C=O), 100.7 (d, C-1′), 78.8 (d, C-4), 76.3 (d, C-3), 72.8 (d, C-3′), 71.8 (d, C-5′), 71.3 (d, C-2′), 68.5 (d, C-4′), 67.8 (t, C-9), 67.7 (d, C-2), 63.3 (t, C-6), 62.0 (t, C-6′), 57.4 (t, C-5), 51.3 (t, C-7), 28.1 (t, C-8), 21.0, 20.9, 20.8, 20.6, 20.5 (q, 7C, CH_3_); IR (CDCl_3_): ν = 2954, 2882, 2261, 1743, 1376, 1234, 1039 cm^−1^; MS (ESI): *m*/*z* calcd (%) for C_28_H_41_NO_16_ + Na^+^ 670.24 [M + Na]^+^; found: 670.36; elemental analysis calcd (%) for C_28_H_41_NO_16_ (647.62): C 51.93, H 6.38, N 2.16; found: C 51.90, H 6.32, N 2.14.

*Compound* **26**α. Obtained as a yellow oil in 37% yield starting from 0.095 mmol of **23**α,β. *R*_f_ = 0.25 (Et_2_O:CH_2_Cl_2_ 4:1); [α${]}_{D}^{23}$ = +44.6 (*c* = 0.74 in CHCl_3_); ^1^H-NMR (400 MHz, CDCl_3_): *δ* = 5.46 (t, *J* = 9.8 Hz, 1H, H-3′), 5.07–5.02 (m, 4H, H-3, H-4, H-1′ and H-4′), 4.84 (dd, *J* = 10.2, 3.9 Hz, 1H, H-2′), 4.25 (dd, *J* = 12.7, 4.4 Hz, 1H, Ha-6′), 4.19 (dd, *J* = 11.7, 6.4 Hz, 1H, Ha-6), 4.12 (dd, *J* = 11.7, 4.8 Hz, 1H, Hb-6), 4.08 (dd, *J* = 12.7, 2.4 Hz, 1H, Hb-6′), 3.99 (ddd, *J* = 10.2, 4.4, 2.4 Hz, 1H, H-5′), 3.68 (dt, *J* = 9.7, 6.3 Hz, 1H, Ha-10), 3.42 (dt, *J* = 9.7, 6.8 Hz, 1H, Hb-10), 3.14 (d, *J* = 11.2 Hz, 1H, Ha-5), 2.80 (dt, *J* = 11.7, 7.8 Hz, 1H, Ha-7), 2.73 (dd, *J* = 10.8, 4.9 Hz, 1H, Hb-5), 2.72–2.70 (m, 1H, H-2), 2.40–2.34 (m, 1H, Hb-7), 2.08 (m, 6H, OAc), 2.07 (s, 3H, OAc), 2.06 (s, 3H, OAc), 2.05 (s, 3H, OAc), 2.02 (s, 3H, OAc), 2.00 (s, 3H, OAc), 1.69–1.48 (m, 4H, H-8 and H-9); ^13^C-NMR (50 MHz, CDCl_3_): *δ* = 170.7, 170.6, 170.5, 170.2, 170.1, 170.0, 169.5 (s, 7C, C=O), 95.8 (d, C-1′), 78.9 (d, C-4), 76.3 (d, C-3), 71.0 (d, C-2′), 70.3 (d, C-3′), 68.8 (d, C-4′), 68.4 (t, C-10), 67.8 (d, C-2), 67.3 (d, C-5′), 63.2 (t, C-6), 62.0 (t, C-6′), 57.3 (t, C-5), 54.4 (t, C-7), 27.1 (t, C-9), 24.7 (t, C-8), 20.9, 20.7, 20.5 (q, 7C, CH_3_); IR (CDCl_3_): ν = 2953, 2872, 2260, 1742, 1455, 1373, 1233, 1038 cm^−1^; MS (ESI): *m*/*z* calcd (%) for C_29_H_43_NO_16_ + Na^+^ 684.26 [M + Na]^+^; found: 683.63; elemental analysis calcd (%) for C_29_H_43_NO_16_ (661.65): C 52.64, H 6.55, N 2.12; found: C 52.61, H 6.59, N 2.17. 

*Compound* **26**β. Obtained as a yellow oil in 9% yield starting from 0.095 mmol of **23**α,β. *R*_f_ = 0.20 (Et_2_O:CH_2_Cl_2_ 4:1); [α${]}_{D}^{23}$ = −35.2 (*c* = 0.17 in CHCl_3_); ^1^H-NMR (400 MHz, CDCl_3_): *δ* = 5.19 (t, *J* = 9.8 Hz, 1H, H-3′), 5.07 (t, *J* = 9.8 Hz, 1H, H-4′), 5.07–5.03 (m, 2H, H-3 and H-4), 4.97 (dd, *J* = 9.8, 7.8 Hz, 1H, H-2′), 4.48 (d, *J* = 7.8 Hz, 1H, H-1′), 4.25 (dd, *J* = 12.2, 4.4 Hz, 1H, Ha-6′), 4.19 (dd, *J* = 11.2, 6.3 Hz, 1H, Ha-6), 4.12 (dd, *J* = 12.2, 2.4 Hz, 1H, Hb-6′), 4.10 (dd, *J* = 11.2, 4.9 Hz, 1H, Hb-6), 3.87 (dt, *J* = 9.8, 5.9 Hz, 1H, Ha-10), 3.68 (ddd, *J* = 9.8, 4.4, 2.4 Hz, 1H, H-5′), 3.47 (dt, *J* = 9.8, 6.8 Hz, 1H, Hb-10), 3.11 (d, *J* = 11.2 Hz, 1H, Ha-5), 2.77 (dt, *J* = 12.2, 7.8 Hz, 1H, Ha-7), 2.70 (dd, *J* = 11.2, 5.2 Hz, 1H, Hb-5), 2.70–2.67 (m, 1H, H-2), 2.33 (ddd, *J* = 12.2, 8.8, 4.4 Hz, 1H, Hb-7), 2.07 (m, 6H, OAc), 2.06 (s, 3H, OAc), 2.03 (s, 3H, OAc), 2.02 (s, 3H, OAc), 2.01 (s, 3H, OAc), 1.99 (s, 3H, OAc), 1.63–1.42 (m, 4H, H-8 and H-9); ^13^C-NMR (50 MHz, CDCl_3_): *δ* = 170.7, 170.5, 170.1, 170.0, 169.5, 169.3, 169.1 (s, 7C, C=O), 100.7 (d, C-1′), 78.9 (d, C-4), 76.3 (d, C-3), 72.9 (d, C-3′), 71.8 (d, C-5′), 71.4 (d, C-2′), 69.6 (t, C-10), 68.6 (d, C-4′), 67.8 (d, C-2), 63.1 (t, C-6), 62.0 (t, C-6′), 57.2 (t, C-5), 54.2 (t, C-7), 27.1 (t, C-9), 24.3 (t, C-8), 20.9, 20.8, 20.7, 20.6, 20.5, 20.4 (q, 7C, CH_3_); IR (CDCl_3_): ν = 2957, 2886, 2816, 2261, 1743, 1454, 1373, 1233, 1039 cm^−1^; MS (ESI): *m*/*z* calcd (%) for C_29_H_43_NO_16_ + Na^+^ 684.26 [M + Na]^+^; found: 683.90; elemental analysis calcd (%) for C_29_H_43_NO_16_ (661.65): C 52.64, H 6.55, N 2.12; found: C 52.60, H 6.52, N 2.11.

#### 3.3.6. Synthesis of Compounds **9**–**11**

General procedure for the synthesis of the α,β mixture of polyhydroxylated pseudodisaccharides: To a 0.01 M solution of the α,β mixture of benzylated derivatives **21**–**23**α,β in methanol, Pd/C (50%, *w*/*w*) and two drops of 37% HCl were added under nitrogen atmosphere, then the mixture was stirred under hydrogen atmosphere at room temperature for 1–3 days, until a NMR control showed the disappearance of the starting material. The mixture was then filtered through Celite^®^ and the solvent was removed under reduced pressure affording a crude yellow oil. The α,β mixture of free amines was obtained by passing the corresponding hydrochloride salts mixture through a Dowex 50WX8 ion-exchange resin. Elution with 6% NH_4_OH afforded the free bases **9**α,β, **10**α,β and **11**α,β with 32–40% yields.

Compounds **9**α,β: The general procedure applied to 144 mg (0.146 mmol) of **21**α,β, stirring the mixture for 3 days, afforded 17 mg (0.05 mmol) of a 2:1 **9**α,β mixture in 35% yield.

Compounds **10**α,β: The general procedure applied to 100 mg (0.102 mmol) of **22**α,**β**, stirring the mixture for one day, afforded 14 mg (0.04 mmol) of a 1:1 **10**α,β mixture in 40% yield.

Compounds **11**α,β: The general procedure applied to 192 mg (0.192 mmol) of **23**α,β, stirring the mixture for 3 days, afforded 21 mg (0.06 mmol) of a 1.2:1 **11**α,β mixture in 32% yield.

*General procedure for the synthesis of polyhydroxylated α-pseudodisaccharides:* A suspension of α-peracetylated derivatives **24**α–**26**α (1 equiv.) and ion exchange resin Ambersep 900 OH (600 mg) in 5–10 mL of methanol was slowly stirred at room temperature for 16 h. After filtration through Celite^®^, the solvent was removed under reduced pressure affording pure α-pseudodisaccharides **9α**–**11α** in quantitative yields.

*Compound* **9α**. Obtained as a yellow oil in quantitative yield starting from 0.063 mmol of **24**α. [α${]}_{D}^{27}$ = +48.4 (*c* = 1.11 in MeOH); ^1^H-NMR (400 MHz, D_2_O): *δ* = 4.72 (d, *J* = 3.9 Hz, 1H, H-1′), 3.93 (ddd, *J* = 5.4, 2.9, 2.4 Hz, 1H, H-4), 3.73 (dd, *J* = 4.9, 2.9 Hz, 1H, H-3), 3.67 (dd, *J* = 12.2, 2.4 Hz, 1H, Ha-6′), 3.67–3.64 (m, 1H, Ha-8), 3.56 (dd, *J* = 12.2, 5.4 Hz, 1H, Hb-6′), 3.55–3.50 (m, 3H, H-3′ and H-6), 3.47 (ddd, *J* = 9.7, 5.4, 2.4 Hz, 1H, H-5′), 3.39 (ddd, *J* = 12.2, 8.3, 4.4 Hz, 1H, Hb-8), 3.34 (dd, *J* = 9.7, 3.9 Hz, 1H, H-2′), 3.21 (t, *J* = 9.7 Hz, 1H, H-4′), 2.97 (ddd, *J* = 13.2, 7.8, 4.4 Hz, 1H, Ha-7), 2.86 (dd, *J* = 11.3, 2.0 Hz, 1H, Ha-5), 2.61 (dd, *J* = 11.3, 5.9 Hz, 1H, Hb-5), 2.46 (dt, *J* = 13.2, 4.4 Hz, 1H, Hb-7), 2.38 (q, *J* = 5.4 Hz, 1H, H-2); ^13^C-NMR (100 MHz, D_2_O): *δ* = 98.4 (d, C-1′), 78.9 (d, C-3), 75.4 (d, C-4), 73.1 (d, C-3′), 72.0 (d, C-5′), 71.9 (d, C-2′), 71.4 (d, C-2), 69.5 (d, C-4′), 65.6 (t, C-8), 61.1 (t, C-6), 60.5 (t, C-6′), 58.6 (t, C-5), 54.1 (t, C-7); MS (ESI): *m*/*z* calcd (%) for C_13_H_25_NO_9_ + Na^+^ 362.15 [M + Na]^+^; found: 361.52; elemental analysis calcd (%) for C_13_H_25_NO_9_ (339.34): C 46.01, H 7.43, N 4.13; found: C 45.98, H 7.48, N 4.16.

*Compound* **10**α. Obtained as a yellow oil in quantitative yield starting from 0.046 mmol of **25**α. [α${]}_{D}^{26}$ = +34.1 (*c* = 0.81 in MeOH); ^1^H-NMR (400 MHz, D_2_O): *δ* = 4.71 (d, *J* = 3.4 Hz, 1H, H-1′), 3.92 (ddd, *J* = 5.4, 2.9, 2.0 Hz, 1H, H-4), 3.73 (dd, *J* = 5.4, 2.9 Hz, 1H, H-3), 3.66 (dd, *J* = 12.2, 2.4 Hz, 1H, Ha-6′), 3.56 (dd, *J* = 12.2, 5.4 Hz, 1H, Hb-6′), 3.54–3.50 (m, 4H, H-3′, H-6 and Ha-9), 3.46 (ddd, *J* = 9.7, 5.4, 2.4 Hz, 1H, H-5′), 3.40–3.36 (m, 1H, Hb-9), 3.35 (dd, *J* = 9.7, 3.5 Hz, 1H, H-2′), 3.21 (t, *J* = 9.7 Hz, 1H, H-4′), 2.82 (dd, *J* = 11.2, 1.9 Hz, 1H, Ha-5), 2.82–2.76 (m, 1H, Ha-7), 2.55 (dd, *J* = 11.2, 5.9 Hz, 1H, Hb-5), 2.35 (q, *J* = 5.4 Hz, 1H, H-2), 2.26 (ddd, *J* = 11.7, 9.8, 5.9 Hz, 1H, Hb-7), 1.72–1.60 (m, 2H, H-8); ^13^C-NMR (100 MHz, D_2_O): *δ* = 98.0 (d, C-1′), 79.0 (d, C-3), 75.3 (d, C-4), 73.0 (d, C-3′), 71.8 (d, C-5′), 71.3 (d, 2C, C-2′ and C-2), 69.5 (d, C-4′), 66.2 (t, C-9), 61.2 (t, C-6), 60.5 (t, C-6′), 58.2 (t, C-5), 52.2 (t, C-7), 26.9 (t, C-8); MS (ESI): *m*/*z* calcd (%) for C_14_H_27_NO_9_^+^ 353.17 [M]^+^; found: 352.96; elemental analysis calcd (%) for C_14_H_27_NO_9_ (353.37): C 47.59, H 7.70, N 3.96; found: C 47.55, H 7.68, N 3.93.

*Compound* **11**α. Obtained as a yellow oil in quantitative yield starting from 0.023 mmol of **26**α. [α${]}_{D}^{26}$ = +33.1 (*c* = 0.81 in MeOH); ^1^H-NMR (400 MHz, D_2_O): *δ* = 4.72 (d, *J* = 3.9 Hz, 1H, H-1′), 3.92 (ddd, *J* = 5.4, 2.4, 2.0 Hz, 1H, H-4), 3.73 (dd, *J* = 4.9, 3.0 Hz, 1H, H-3), 3.67 (dd, *J* = 12.2, 2.5 Hz, 1H, Ha-6′), 3.58–3.46 (m, 6H, H-3′, Hb-6′, H-5′, H-6 and Ha-10), 3.35 (dd, *J* = 9.7, 3.9 Hz, 1H, H-2′), 3.35–3.32 (m, 1H, Hb-10), 3.21 (t, *J* = 9.7 Hz, 1H, H-4′), 2.82 (dd, *J* = 11.2, 1.5 Hz, 1H, Ha-5), 2.66 (ddd, *J* = 11.7, 10.7, 5.4 Hz, 1H, Ha-7), 2.55 (dd, *J* = 11.2, 5.3 Hz, 1H, Hb-5), 2.34 (q, *J* = 5.4 Hz, 1H, H-2), 2.21 (dt, *J* = 11.7, 4.4 Hz, 1H, Hb-7), 1.49–1.41 (m, 2H, H-8), 1.40–1.30 (m, 2H, H-9); ^13^C-NMR (100 MHz, D_2_O): *δ* = 98.0 (d, C-1′), 79.1 (d, C-3), 75.4 (d, C-4), 73.1 (d, C-3′), 71.8 (d, C-5′), 71.7 (d, C-2), 71.3 (d, C-2′), 69.6 (d, C-4′), 67.8 (t, C-10), 61.3 (t, C-6), 60.5 (t, C-6′), 58.2 (t, C-5), 52.4 (t, C-7), 26.7 (t, C-8), 23.6 (t, C-9); MS (ESI): *m*/*z* calcd (%) for C_15_H_29_NO_9_ + Na^+^ 390.18 [M + Na]^+^; found: 390.42; elemental analysis calcd (%) for C_15_H_29_NO_9_ (367.39): C 49.04, H 7.96, N 3.81; found: C 49.03, H 7.98, N 3.83.

### 3.4. Synthesis of Compound ***9**β*

#### 3.4.1. Synthesis of 2,3,4,6-Tetra-*O*-acetyl-α/β-d-glucopyranose **28** [18]

To a solution of ethylenediamine (93 mg, 1.54 mmol) in 10 mL of dry THF, acetic acid (102 μL, 1.79 mmol) was slowly added dropwise over 10 min and the reaction mixture was stirred under nitrogen atmosphere at room temperature for 1 h. Then **27** (500 mg, 1.28 mmol) was added and the mixture was stirred for 1 h, until TLC analysis (PE/EtOAc 3:2) showed the disappearance of the starting material (*R*_f_ = 0.48) and the formation of a new compound (*R*_f_ = 0.24). After washing with HCl 0.1 M (2 × 2 mL) and NaHCO_3_ (2 × 2 mL), the combined organic layers were dried on Na_2_SO_4_, concentrated at reduced pressure and the crude mixture was purified by FCC (PE/AcOEt 1:1) affording pure **28** (α/β 1:9 *R*_f_ = 0.30, PE/EtOAc 1:1, 383 mg, 1.100 mmol, 86% yield).

^1^H-NMR (200 MHz, CDCl_3_): *δ* = 5.62 (d, *J*= 9.8 Hz 1H, H-1α), 5.55 (t, *J*= 3.8 Hz, 1H, H-3α), 5.36–5.27 (m, 1H, H-3β), 5.14 (t, *J* = 9.6 Hz, 1Hα + 1Hβ, H-4), 4.96 (dd, *J* = 9.8, 3.4 Hz, 1Hα + 1 Hβ, H-2), 4.80 (t, *J*= 8.1 Hz, 1H, H-1β), 4.37–4.12 (m, 5H, H-6α, H-6 β, H-5α), 3.85–3.77 (m, 1H, H-5β), 2.16 (s, 3Hα + 3 Hβ, OAc), 2.15 (s, 3Hα + 3 Hβ, OAc), 2.09 (s, 3Hα + 3 Hβ, OAc), 2.08 (s, 3Hα + 3 Hβ, OAc) ppm.

#### 3.4.2. Synthesis of 2,3,4,6-Tetra-*O*-acetyl-α-d-glucopyranosyltrichloroacetimidate **29** [19]

A solution of **28** (383 mg, 1.10 mmol) in 8 mL of dry CH_2_Cl_2_ was cooled to 0 °C and DBU (32 μL, 0.22 mmol) and trichloroacetonitrile (612 μL, 7.70 mmol) were added. The reaction mixture was stirred under nitrogen atmosphere at room temperature for 2.5 h, when a TLC analysis (Hex/EtOAc 1:1) showed the disappearance of the starting material (*R*_f_ = 0.26) and the formation of a new compound (*R*_f_ = 0.62). A saturated NH_4_Cl solution was added and the mixture was transferred to a separating funnel, washing with CH_2_Cl_2_. The organic layer was washed with water (3 × 5 mL) and dried over Na_2_SO_4_. After concentration under reduced pressure, the crude was purified by flash column chromatography on silica gel (Hex/EtOAc from 3:1 to 2:1) to afford pure **29** (*R*_f_ = 0.25 Hex/EtOAc 2:1, 461 mg, 0.939 mmol, 85% yield).

^1^H-NMR (200 MHz, CDCl_3_): *δ* = 8.69 (s, 1H, NH), 6.56 (d, *J* = 3.7 Hz, 1H, H-1), 5.57 (t, *J* = 9.8 Hz, 1H, H-4), 5.23–5.10 (m, 2H, H-2, H-3), 4.32–4.06 (m, 3H, H-5, H-6 ), 2.08 (s, 3H, OAc), 2.05 (s, 3H, OAc), 2.04 (s, 3H, OAc), 2.02 (s, 3H, OAc) ppm.

#### 3.4.3. Synthesis of Compound **30**

A solution of glucopyranosyl tricholoroacetimidate **29** (2 equiv.) and alcohol **15** (1 equiv.) in dry CH_2_Cl_2_, was stirred for 10 min at room temperature under nitrogen atmosphere in the presence of 3 Å molecular sieves. After cooling to 0 °C trimethylsilyl trifluoromethanesulfonate (1.5 equiv.) was added and the mixture was stirred for 1.5 h, letting the temperature to rise. To the reaction mixture 1.8 mL of triethylamine was added and the mixture was transferred to a separating funnel, washing with CH_2_Cl_2._ The organic layer was washed with HCl 1M (3 × 3 mL), NaOH 1M (1 × 3 mL) and brine (2 × 3 mL). The combined organic layers were dried over Na_2_SO_4_ and the solvent was removed under reduced pressure. The resulting crude oil was dissolved in pyridine (3 mL), and acetic anhydride (2 mL) and DMAP (30 μL) were added. The solution was stirred at room temperature overnight, and after concentration under reduced pressure, the crude was purified by flash column chromatography on silica gel (Hex/EtOAc from 1:1 to 1:2) to afford the β compound **30** (*R*_f_ = 0.516 Hex/EtOAc 1:1, 34 mg, 0.044 mmol, 39% yield) as colorless oil, contaminated with small amounts of partially deacetylated glucoside (see supplementary information).

*Compound* **30**: [α${]}_{D}^{21}$ = −15.2 (*c* = 0.80 in CHCl_3_); ^1^H-NMR (400 MHz, CDCl_3_): *δ* =7.32–7.24 (m, 15H, H-Ar), 5.16 (t, *J* = 9.5 Hz, 1H, H-4′), 5.05 (dd, *J* = 9.8, 9.6 Hz, 1H, H-2′), 4.97 (t, *J* = 9.6 Hz, 1H, H-3′), 4.54–4.43 (m, 7H, H-Bn, H-1′), 4.23 (dd, *J* = 12.2, 4.7 Hz, 1H, Ha-6′), 4.09 (dd, *J* = 12.2, 2.2 Hz, 1H, Hb-6′), 4.00–3.95 (m, 1H, Ha-8), 3.88 (d, *J* = 5.0 Hz, 1H, H-4), 3.81 (d, *J* = 3.8 Hz, 1H, H-3), 3.68–3.58 (m, 2H, Hb-8, H-5′), 3.56–3.43 (m, 2H, H-6), 3.16 (d, *J* = 10.7 Hz, 1H, Ha-5), 3.10–3.04 (m, 1H, Ha-7), 2.77 (q, *J* = 5.7 Hz, 1H, H-2), 2.69–2.61 (m, 2H, Hb-5, Hb-7), 2.06 (s, 3H, OAc), 2.01 (s, 3H, OAc), 1.99 (s, 3H, OAc), 1.93 (s, 3H, OAc); ^13^C-NMR (100 MHz, CDCl_3_ ): *δ* = 170.6, 170.2, 169.4, 169.3 (s, 4C, C=O), 138.3, 138.2, 138.1 (t, 3C, C-Ar), 128.3, 127.8, 127.7, 127.6, 127.5 (d, 15C, C-Ar), 100.6 (d, C-1′), 84.7 (d, C-4), 81.6 (d, C-3), 77.4 (C-2′), 77.1 (d, C-3′), 73.2 (d, C-4′), 72.8 (d, C-2), 71.2, 71.1, 71.0 (s, 3C, C-Ar), 69.4 (d, C-5′), 68.9 (t, C-8), 68.4 (t, C-6), 61.9 (t, C-6′), 58.4 (t, C-5), 54.0 (t, C-7), 20.7, 20.6, 20.6, 20.6 (q, 4C, CH_3_); IR (CDCl_3_): ν = 3031, 2945, 2866, 2360, 2331, 1755, 1497, 1454, 1375, 1231, 1171,1039 cm^−1^. MS (ESI): *m*/*z* calcd (%) for C_42_H_51_NO_13_ + Na^+^ 800.33 [M + Na]^+^; found: 800.63.

#### 3.4.4. Synthesis of Compound **9**β

To a solution of **30** (43 mg, 0.055 mmol) in 10 mL of methanol, Pd/C (50%, *w*/*w*) and 0.5 mL of HCl 1 M were added under nitrogen atmosphere, then the mixture was stirred under hydrogen atmosphere at room temperature overnight, until an ^1^H-NMR control showed the disappearance of the starting material. The mixture was then filtered through Celite^®^ and the solvent was removed under reduced pressure. The crude was purified by flash column chromatography on silica gel (CH_2_Cl_2_/MeOH 10:1, *R*_f_ = 0.585) to afford 12 mg, which were dissolved in 10 mL of methanol, ion exchange resin Ambersep 900-OH was added and the suspension was slowly stirred at room temperature for 16 h. The mixture was filtered through cotton and the solvent was removed under reduced pressure, affording pure **9**β (8 mg, 0.024 mmol, 43% yield) as colorless oil.

*Compound* **9**β: [α${]}_{D}^{19}$ = −28.5 (*c* = 0.67 in H_2_O); ^1^H-NMR (400 MHz, D_2_O): *δ* = 4.34 (d, *J* = 8.0 Hz, 1H, H-1′), 4.02–3.98 (m, 1H, H-4), 3.92 (ddd, *J* = 11.0, 6.4, 4.4 Hz, 1H, Ha-8), 3.82–3.81 (m, 1H, H-3), 3.77 (d, *J* = 2 Hz, 1H, Ha-6′), 3.68–3.56 (m, 4H, Hb-8, H-6, Hb-6′), 3.38–3.21 (m, 3H, H-3′, H-4′, H-5′), 3.15 (t, *J* = 8.4 Hz, 1H, H-2′), 3.05–2.97 (m, 2H, Ha-7, Ha-5), 2.74 (dd, *J* = 11.4, 5.7 Hz, 1H, Hb-5), 2.61–2.53 (m, 2H, Hb-7, H-2); ^13^C-NMR (100 MHz, D_2_O): *δ* = 102.32 (d, C-1′), 79.11 (d, C-3), 76.06 (d, C-3′), 75.79 (d, C-5′), 75.62 (d, C-4), 73.20 (d, C-2′), 72.01 (d, C-2), 69.78 (d, C-4′), 67.88 (t, C-8), 61.19 (t, C-6), 60.88 (t, C-6′), 58.94 (t, C-5), 54.04 (t, C-7); MS (ESI): *m*/*z* calcd (%) for C_13_H_25_NO_9_ + Na^+^ 362.15 [M + Na]^+^; found: 362.47; elemental analysis calcd (%) for C_13_H_25_NO_9_ (339.34): C 46.01, H 7.43, N 4.13; found: C 46.67, H 7.45, N 3.20.

### 3.5. Biological Evaluation of Compounds ***8***, ***9**α*, ***9**β*, ***10**α*, ***11**α* and the α/β Mixture of ***9***,***10*** and ***11***

Compounds were tested for their inhibitory activity against insect trehalase of midge larvae of *C. riparius*, a good model for biochemical studies [20], and porcine kidney trehalase (purchased from Sigma-Aldrich, St. Louis, MO, USA) as the mammalian counterpart.

Trehalase activity was measured through a coupled assay with glucose-6-phosphate dehydrogenase and hexokinase according to Wegener at al. [21] To examine the potential of each compound as a trehalase inhibitor, dose-response curves were established to determine the IC_50_ values. Experiments were performed at a fixed substrate concentration close to the *K*_m_ value (0.5 mM for *C. riparius* and 2.5 mM for porcine trehalase), in the presence of increasing inhibitor concentrations.Initial rates as a function of inhibitor concentration were fitted to the following equation:

(1) $$\frac{v_{i}}{v}=\frac{1}{1+{\left(\frac{\left[I\right]}{{\mathrm{IC}}_{50}}\right)}^{n}}$$

where *v_i_* and *v* are the initial rate in the presence and in the absence of inhibitor, respectively, [*I*] is the inhibitor concentration, IC_50_ is the inhibitor concentration producing half-maximal inhibition, and *n* is the Hill coefficient.

Kinetic experiments were *performed* using *C. riparius* trehalase, measuring enzymatic activity at different trehalose concentrations from 0.05 to 20 mM in the presence of fixed inhibitor concentrations (5–10 µM for the compound **9**α and 0.5–1 µM for the compound **9**β). Kinetic parameters were calculated using a multiparameter, iterative, non-linear regression program based on the Marquardt-Levenberg algorithm (Sigma Plot, Jandel, CA, USA). Data are given ± S.D. of three independent experiments.

All enzyme assays were performed in triplicates at 30 °C by using sample volumes varying from 5 to 20 μL in 1 mL test and using a Cary3 UV/Vis Spectrophotometer. Enzyme activities were analyzed by Cary Win UV application software for Windows XP.

## 4. Conclusions

In conclusion, the synthesis of new pseudodisaccharide mimics **8**, **9**, **10** and **11**, bearing a glucosyl moiety and a pyrrolizidine or pyrrolidine portion, was undertaken and their biological activity as insect trehalase inhibitors was evaluated. Inversion of configuration at C-6 in compound **8** (with respect to previously synthesized compound **4**) resulted in a decrease of potency and selectivity towards the insect trehalase, showing the key role played by the stereochemical configuration at C-6 of the pyrrolizidine nucleus.

Moreover, a simple synthetic strategy was developed to obtain new pseudodisaccharide inhibitors with a pyrrolidine core, demonstrating the pivotal function played by the distance between the glucosyl and the iminosugar pyrrolidine moiety in these flexible inhibitors. Indeed, among a series of new compounds **9**–**11**, only compounds **9** (with a two-carbon atom linker) maintained their inhibitory activity, while compounds **10** and **11** were completely inactive. In particular, the stereoselective synthesis of compound **9**β allowed the identification of a new and selective insect trehalase inhibitor with IC_50_ in the low micromolar range (IC_50_ = 0.78 μM).

## Figures and Tables

**Table 1:** Biological activity (IC_50_) towards C. riparius and porcine trehalases.

Entry	Compound	*C. riparius* Trehalase	Porcine Trehalase	Selectivity ^2^
entry 1	**4**	44 ± 1 nM ^1^	479 ± 45 nM ^1^	10
entry 2	**6**	177 ± 18 nM ^1^	>1 mM ^1^	>5649
entry 3	**7**	175 ± 12 nM ^1^	>1 mM ^1^	>5714
entry 4	**8**	29.49 ± 7.26 μM	190.60± 34.15 μM	6
entry 5	**9**α,β	2.30 ± 0.13 μM	7.67 ± 3.91 μM	3
entry 6	**9**α	9.36 ± 1.49 μM	27.64 ± 5.35 μM	3
entry 7	**9**β	0.784 ± 0.059 μM	5.84 ± 0.26 μM	7
entry 8	**10**α,β	>1000 μM	n.d. ^3^	-
entry 9	**10**α	>1000 μM	n.d. ^3^	-
entry 10	**11**α,β	>1000 μM	n.d. ^3^	-
entry 11	**11**α	>1000 μM	n.d. ^3^	-

^1^ Value taken from Ref. [7]. ^2^ Selectivity is the ratio between IC_50_ value against porcine trehalase and the IC_50_ value against *C. riparius* trehalase. ^3^ n.d. = not determined.

## Supplementary Materials

The following are available online at www.mdpi.com/link, ^1^H and ^13^C-NMR spectra of new compounds and Figures S1–S4. Inhibition kinetics of insect trehalase in the presence of compounds **9**α and **9**β and dose-response curves of compounds **8**, **9**α,β, **9α** and **9**β for insect and porcine trehalase.
