# Supplementary material for: Probing the Influence of Linker Length and Flexibility in the Design and Synthesis of New Trehalase Inhibitors
Source: Molecules. 2018 Feb 16;23(2):436. doi: 10.3390/molecules23020436 (PMC6017881; doi:10.3390/molecules23020436)
Supplement: Supplementary file 1 [file molecules-23-00436-s001.pdf]

Probing the Influence of Linker Length and Flexibility in the Design and Synthesis of New Trehalase Inhibitors

Giampiero D'Adamio[1], Matilde Forcella[2], Paola Fusi[2], Paolo Parenti[3], Camilla Matassini[1,4]*, Xhenti Ferhati[1], Costanza Vanni[1] and Francesca Cardona [1,4,5]*

1) Department of Chemistry 'Ugo Schiff', University of Florence, via della Lastruccia 3-13, Sesto Fiorentino (FI), Italy.;
2) Department of Biotechnology and Biosciences , University of Milano-Bicocca, Piazza della Scienza 2, 20126 Milano, Italy;
3) Department of Earth and Environmental Sciences, University of Milano-Bicocca, Piazza della Scienza 1, 20126 Milano, Italy.
4) Associated with CNR-INO, Via N. Carrara 1, Sesto Fiorentino (FI), Italy
5) Associated with Consorzio Interuniversitario Nazionale di ricerca in Metodologie e Processi Innovativi di Sintesi (CINMPIS)

Supplementary Materials

Table of contents

^{1}H-NMR and ^{13}C-NMR spectra of compound **19α** S3

^{1}H-NMR and ^{13}C-NMR spectra of compound **19β** S4

^{1}H-NMR and ^{13}C-NMR spectra of compound **20** S5

^{1}H-NMR and ^{13}C-NMR spectra of compound **8** S6

^{1}H-NMR and ^{13}C-NMR spectra of compound **16** S7

^{1}H-NMR and ^{13}C-NMR spectra of compound **17** S8

^{1}H-NMR spectrum of the **21α,β** mixture S9

^{1}H-NMR spectrum of the **22α,β** mixture S10

^{1}H-NMR spectrum of the **23α,β** mixture S11

^{1}H-NMR and ^{13}C-NMR spectra of compound **24α** S12

^{1}H-NMR and ^{13}C-NMR spectra of compound **24β** S13

^{1}H-NMR and ^{13}C-NMR spectra of compound **25α** S14

^{1}H-NMR and ^{13}C-NMR spectra of compound **25β** S15

^{1}H-NMR and ^{13}C-NMR spectra of compound **26α** S16

^{1}H-NMR and ^{13}C-NMR spectra of compound **26β** S17

^{1}H-NMR and ^{13}C-NMR spectra of compound **30** S18

^{1}H-NMR spectrum of the **9α,β** mixture S20

^{1}H-NMR spectrum of the **10α,β** mixture S21

^{1}H-NMR spectrum of the **11α,β** mixture S22

^{1}H-NMR and ^{13}C-NMR spectra of compound **9α** S23

^{1}H-NMR and ^{13}C-NMR spectra of compound **9β** S24

^{1}H-NMR and ^{13}C-NMR spectra of compound **10α** S25

^{1}H-NMR and ^{13}C-NMR spectra of compound **11α** S26

Figure S1. Dose-response curves of compounds 8 (A), 9α,β (B), 9α (C), 9β (D) for insect trehalase S27

Figure S2. Dose-response curves of compounds 8 (A), 9α,β (B), 9α (C), 9β (D) for porcine trehalase S28

Figure S3. Inhibition kinetics of insect trehalase in the presence of compound **9α** S29

Figure S4. Inhibition kinetics of insect trehalase in the presence of compound **9β** S30

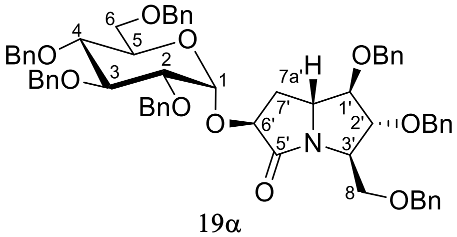

19α

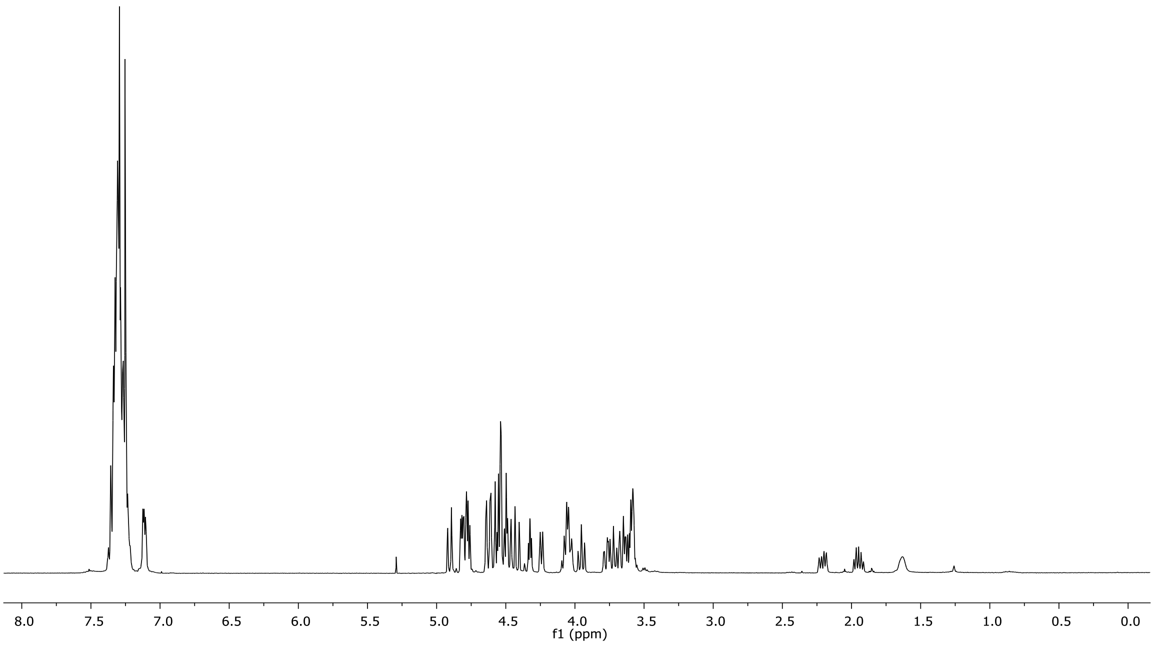

^{1}H-NMR spectrum of compound 19α (400 MHz, $CDCl_3$)

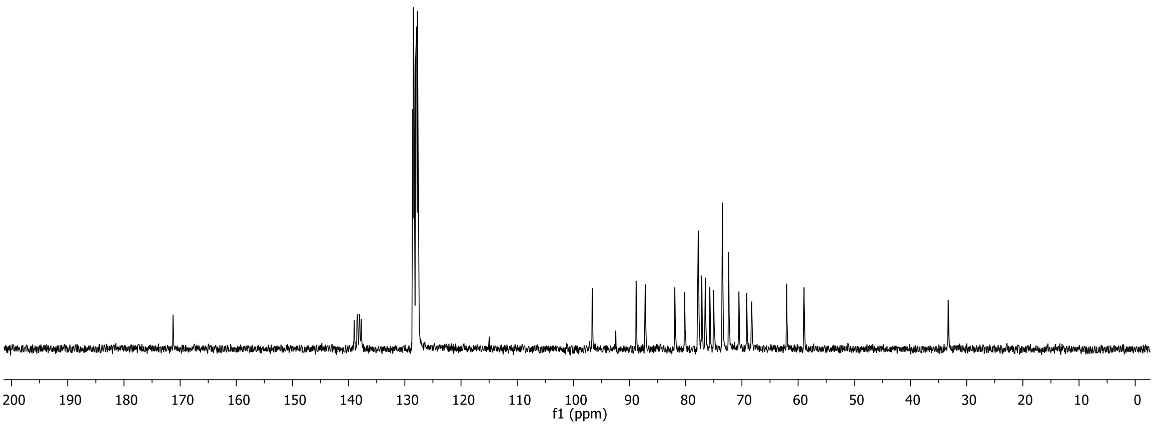

^{13}C-NMR spectrum of compound 19α (50 MHz, $CDCl_3$)

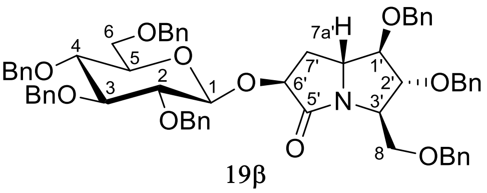

19β

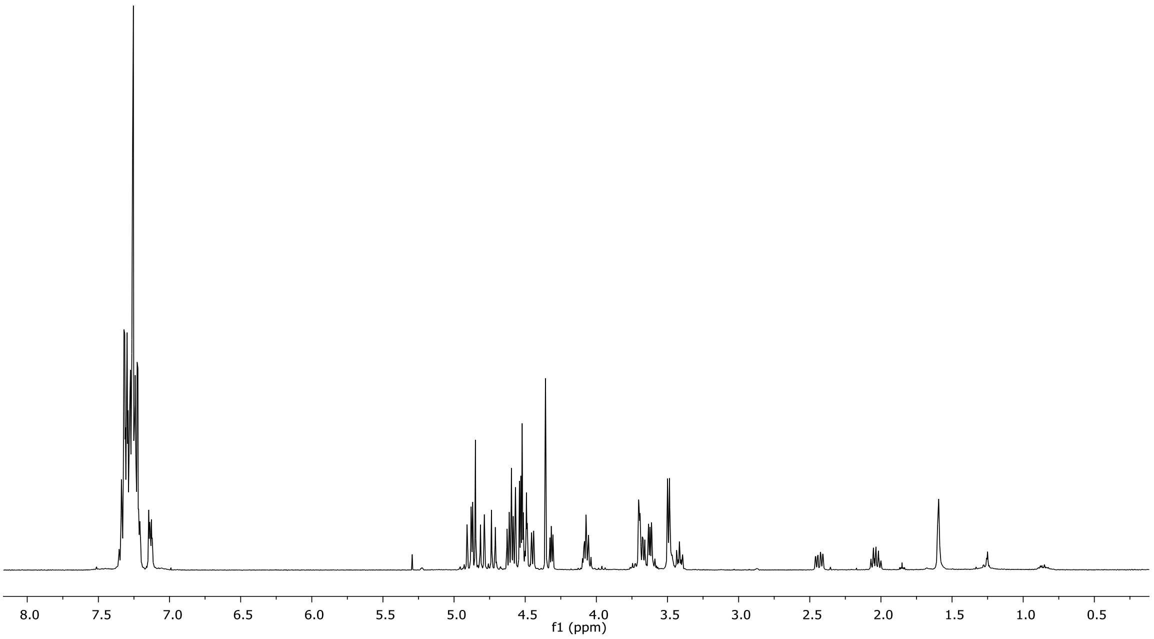

^{1}H-NMR spectrum of compound 19β (400 MHz, CDCl$_3$)

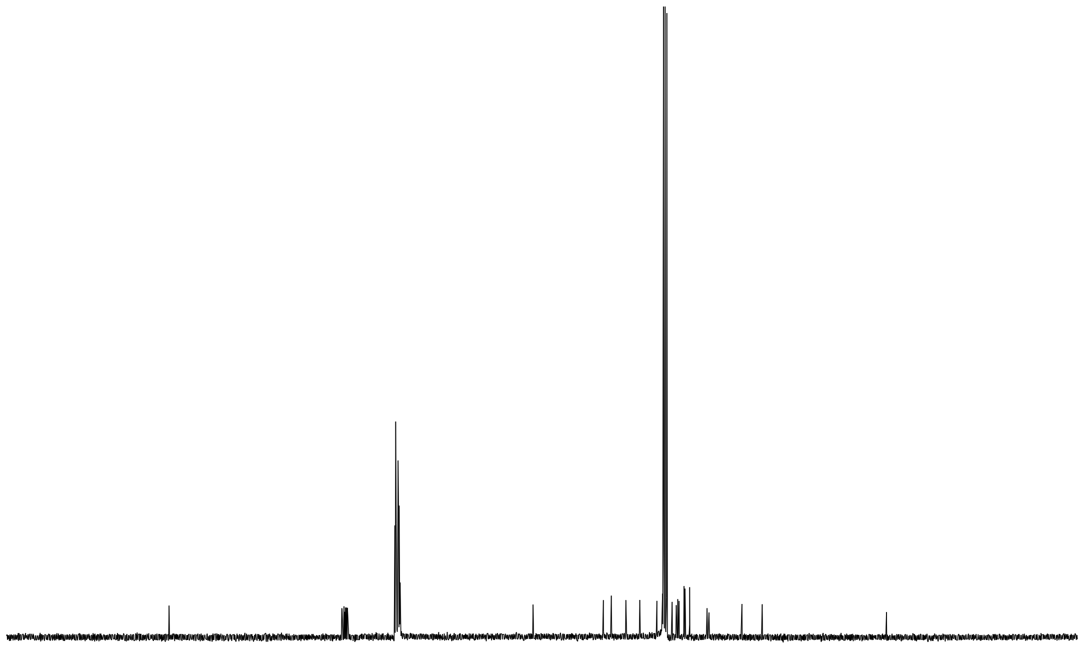

^{13}C-NMR spectrum of compound 19β (100 MHz, CDCl$_3$)

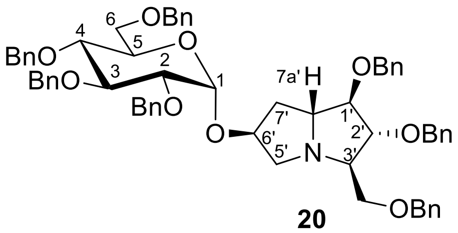

20

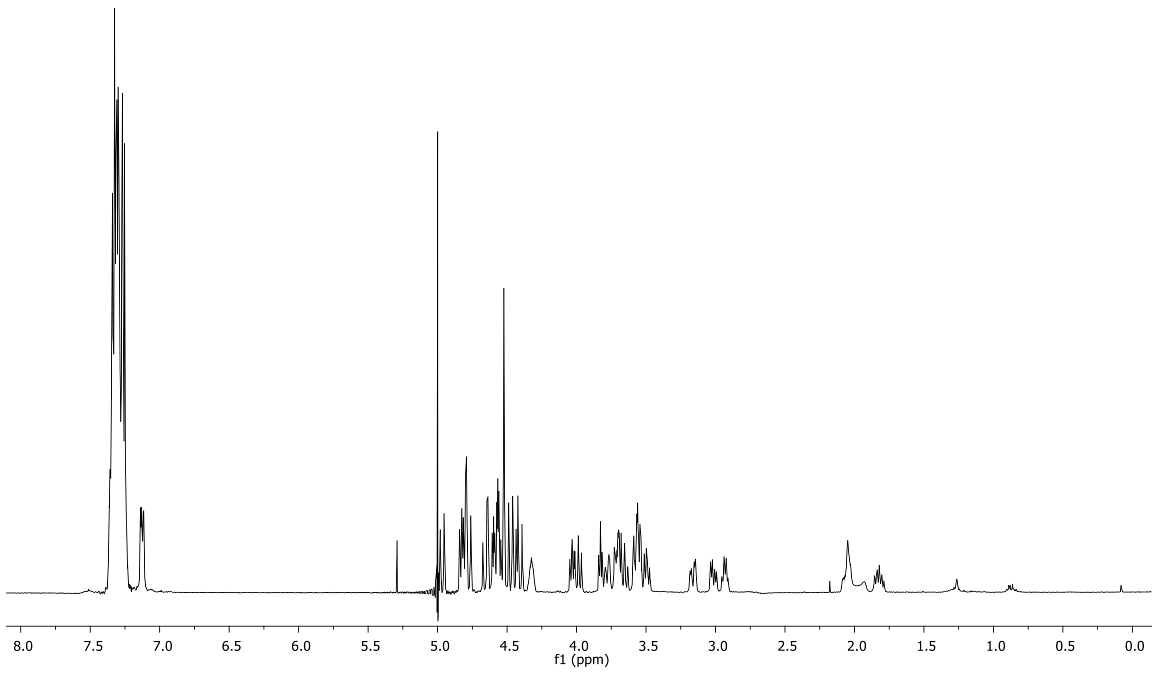

^{1}H-NMR spectrum of compound 20 (400 MHz, $CDCl_3$)

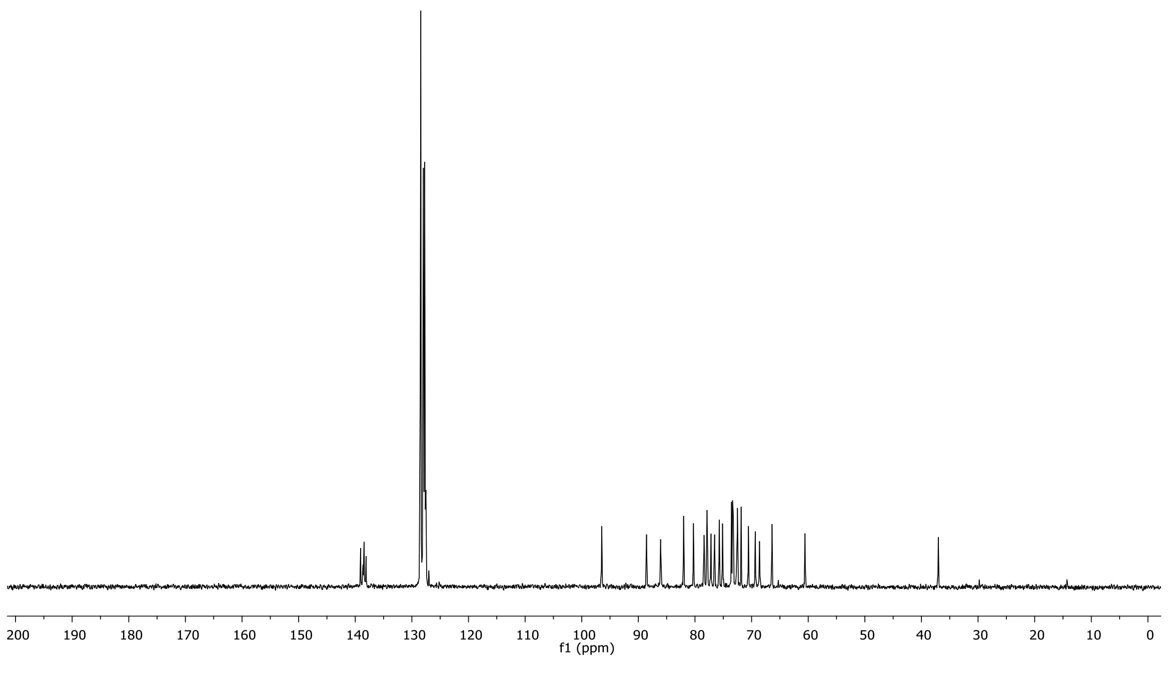

^{13}C-NMR spectrum of compound 20 (50 MHz, $CDCl_3$)

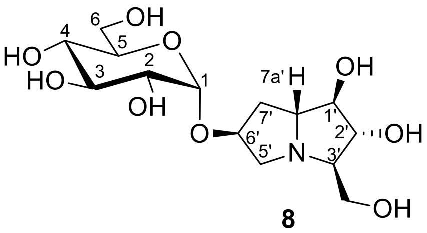

8

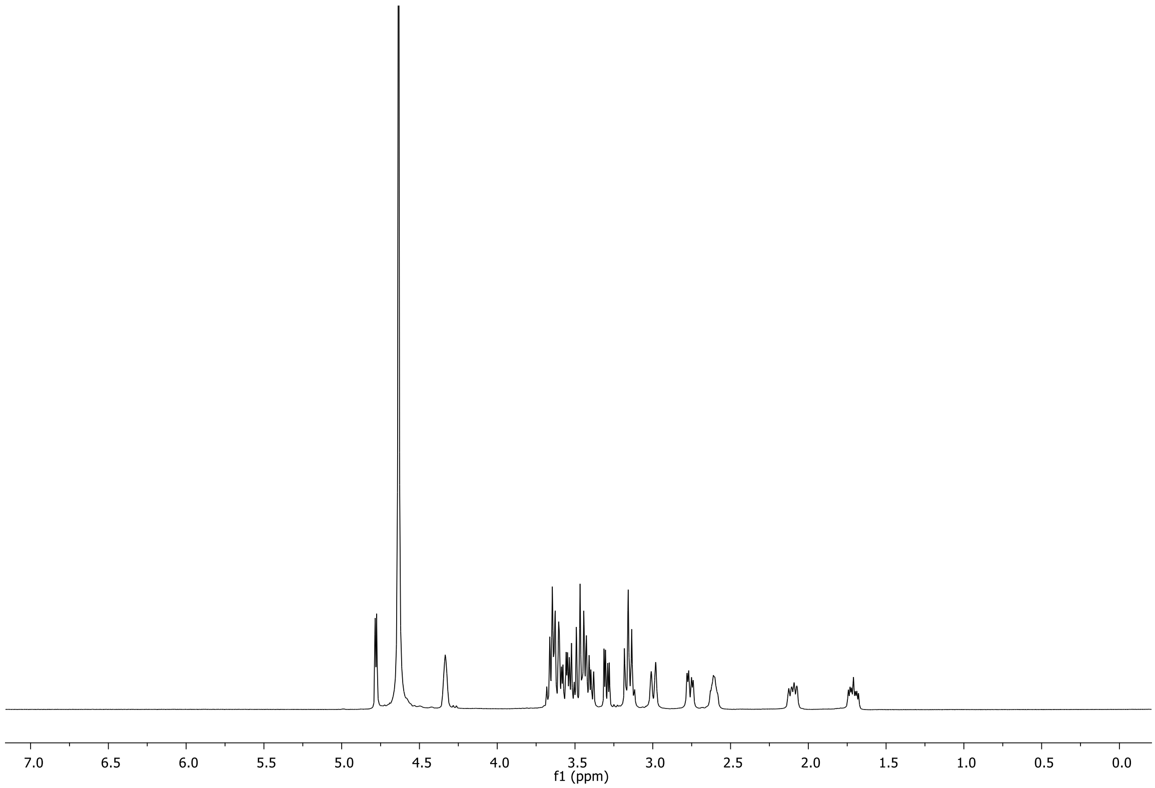

^{1}H-NMR spectrum of compound 8 (400 MHz, D_2O)

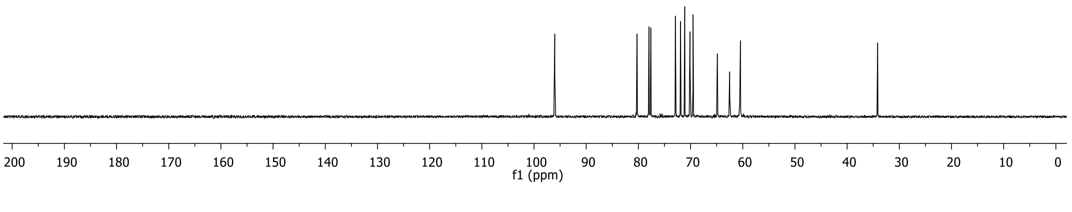

^{1}H-NMR spectrum of compound 8 (100 MHz, D_2O)

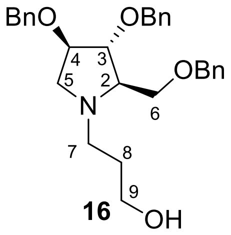

16

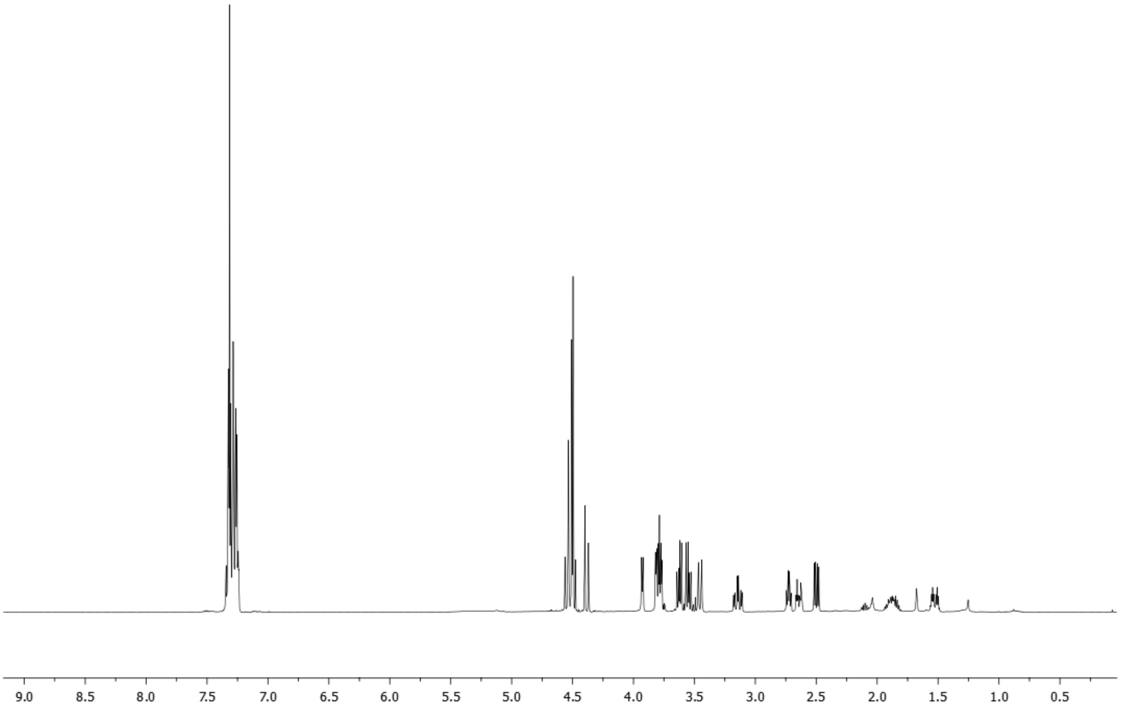

^{1}H-NMR spectrum of compound 16 (400 MHz, $CDCl_3$)

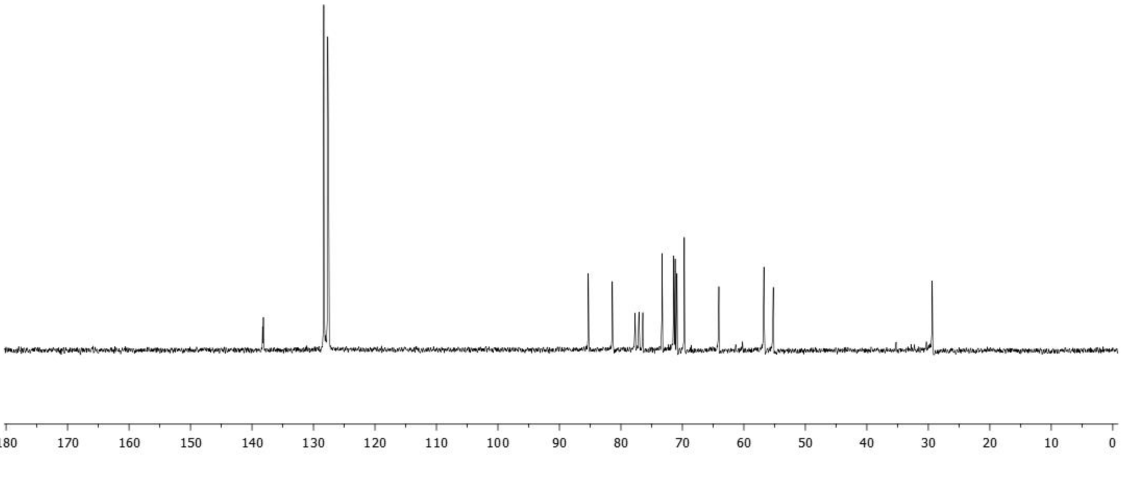

^{13}C-NMR spectrum of compound 16 (50 MHz, $CDCl_3$)

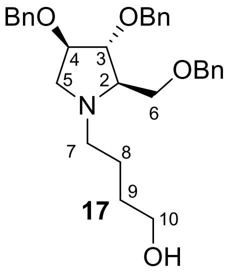

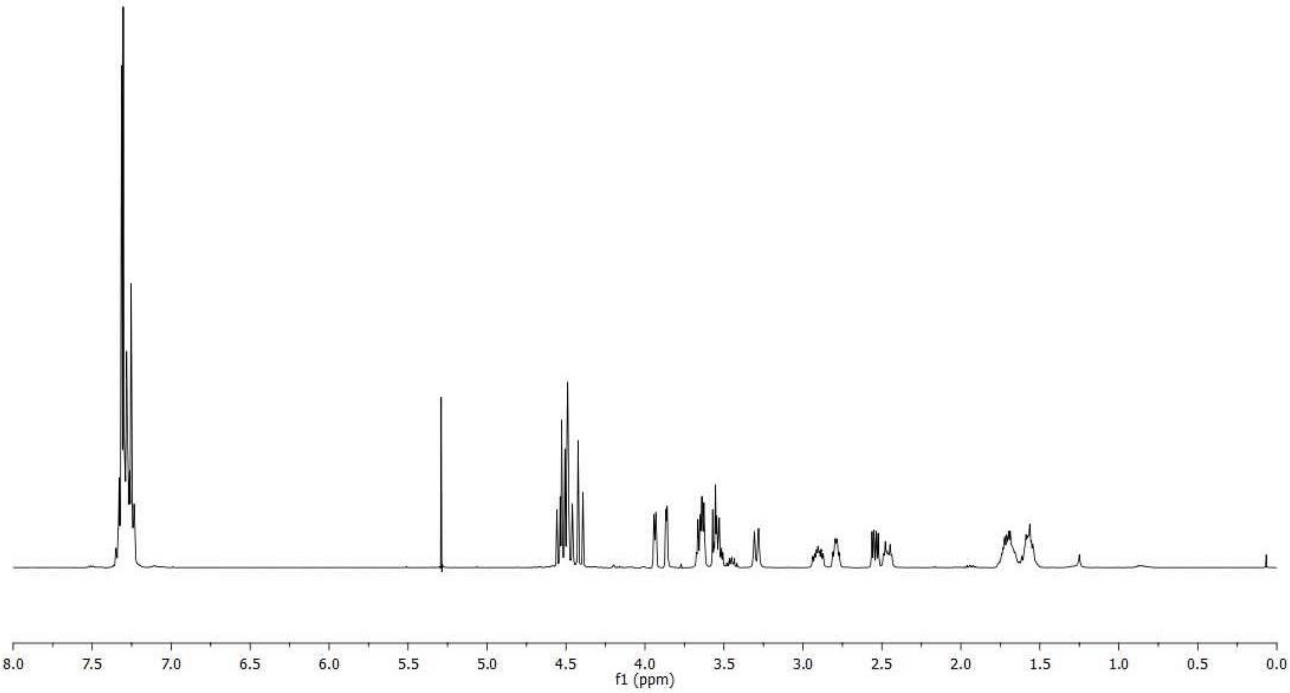

^{1}H-NMR spectrum of compound 17 (400 MHz, $CDCl_3$)

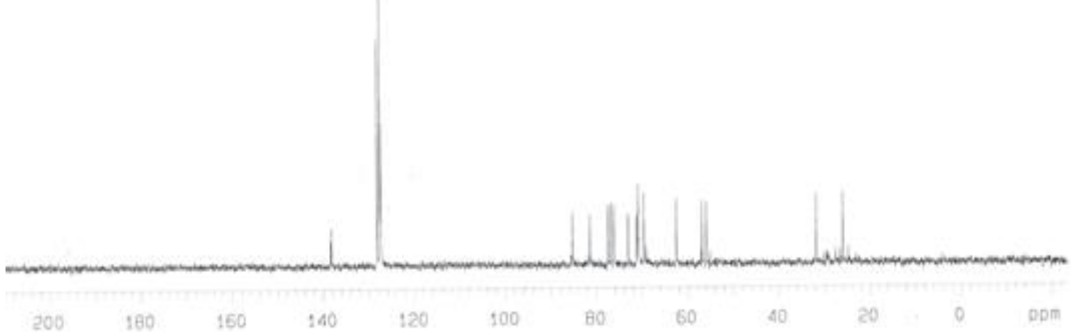

^{13}C-NMR spectrum of compound 17 (50 MHz, $CDCl_3$)

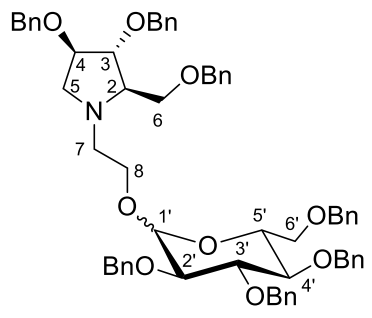

21α,β

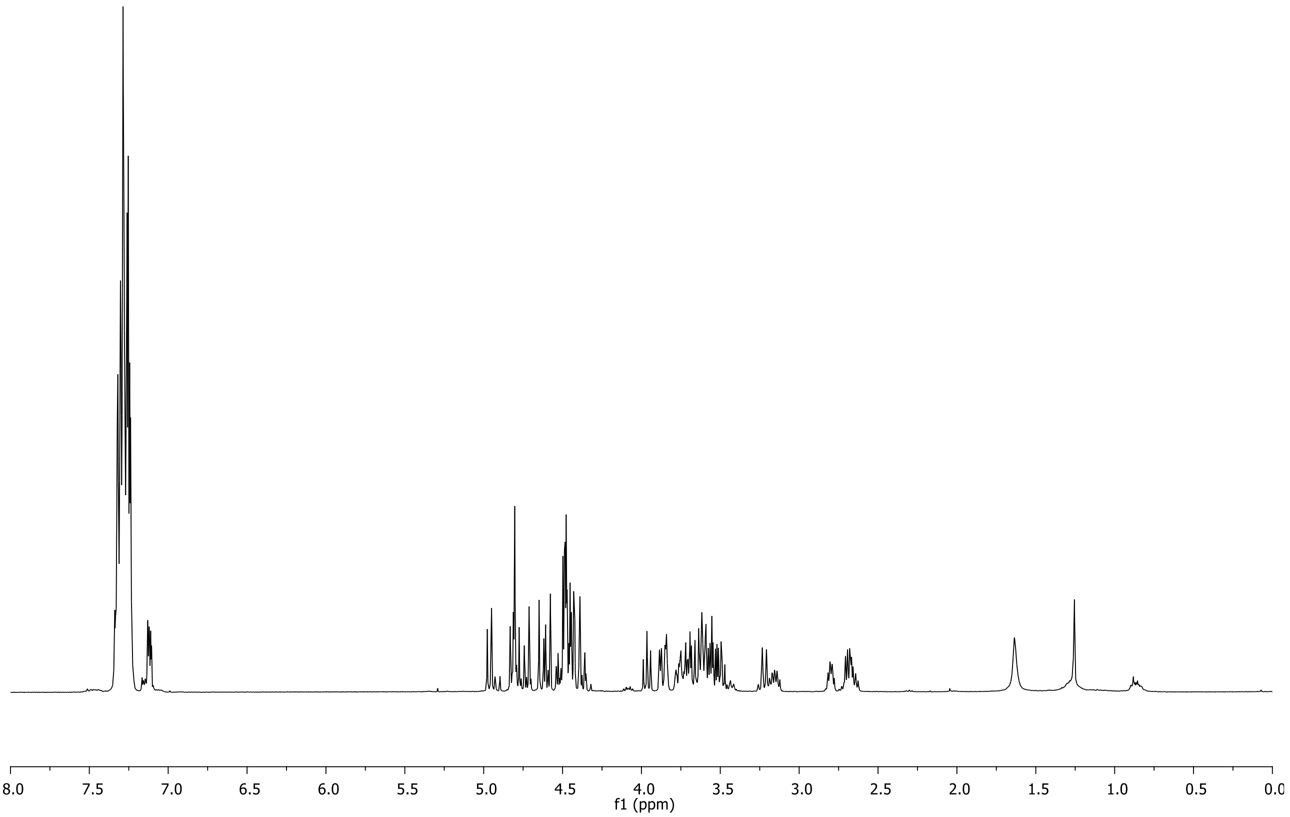

^{1}H-NMR spectrum of the purified mixture of anomers 21α and 21β (400 MHz, CDCl$_3$)

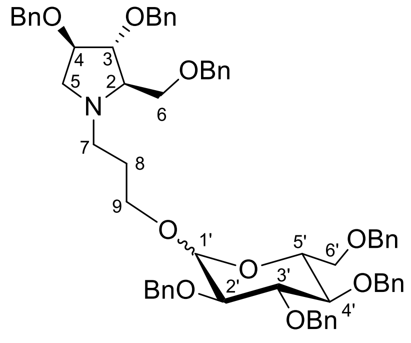

22α,β

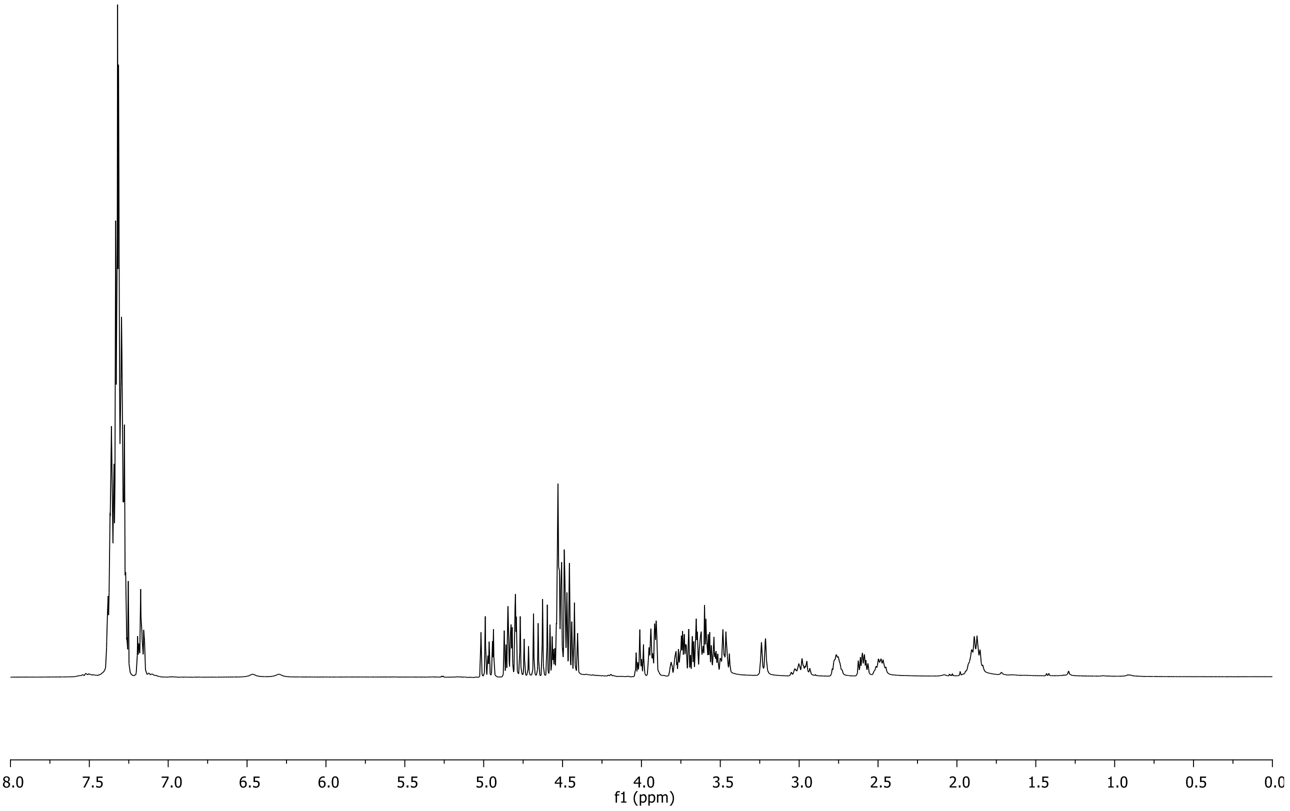

^{1}H-NMR spectrum of the purified mixture of anomers 22α and 22β (400 MHz, $CDCl_3$)

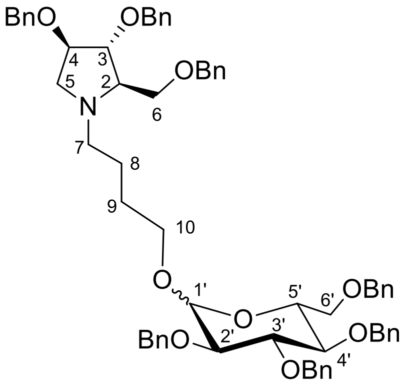

23α,β

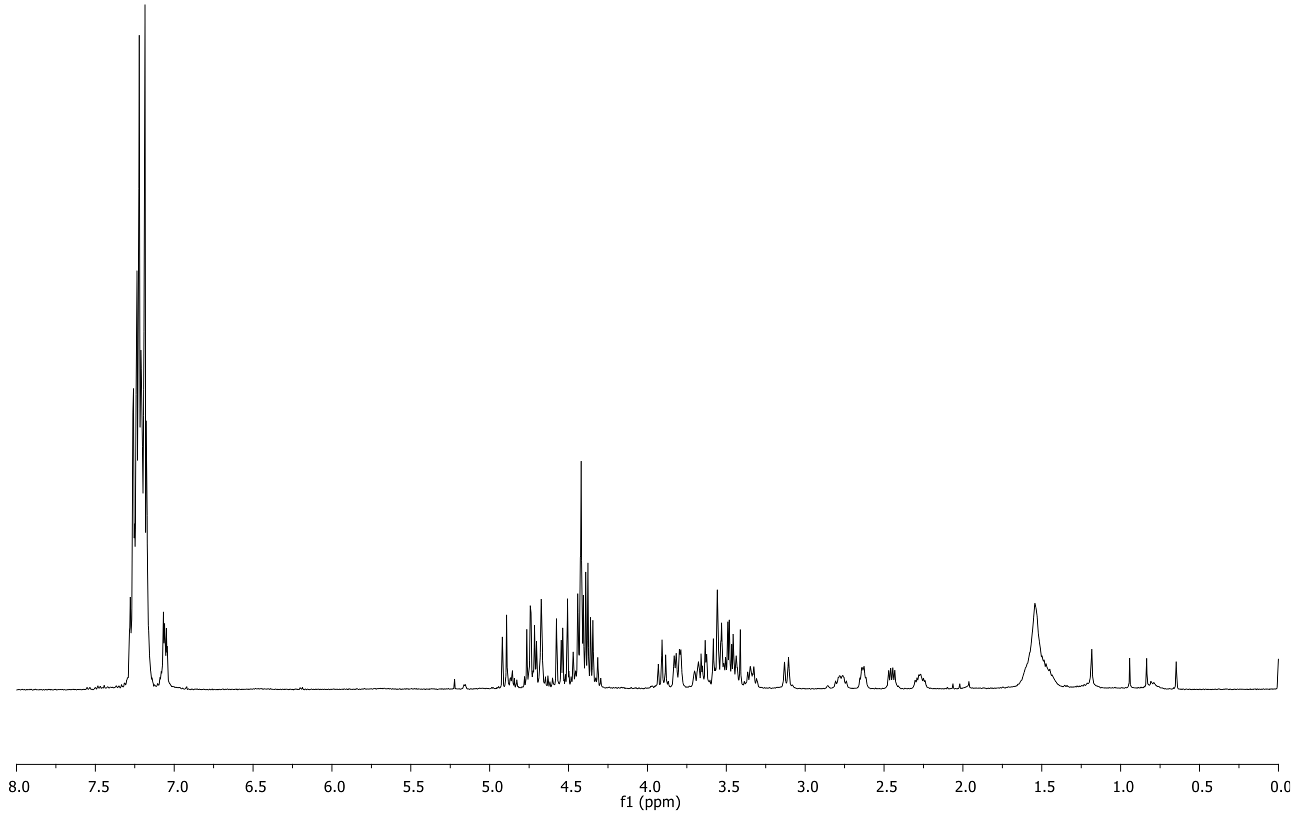

^{1}H-NMR spectrum of the purified mixture of anomers 23α and 23β (400 MHz, $CDCl_3$).

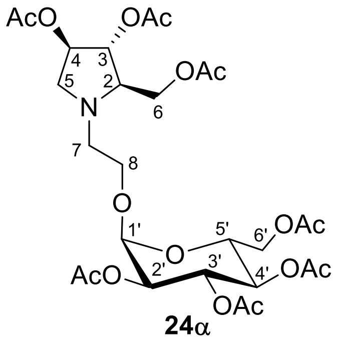

24α

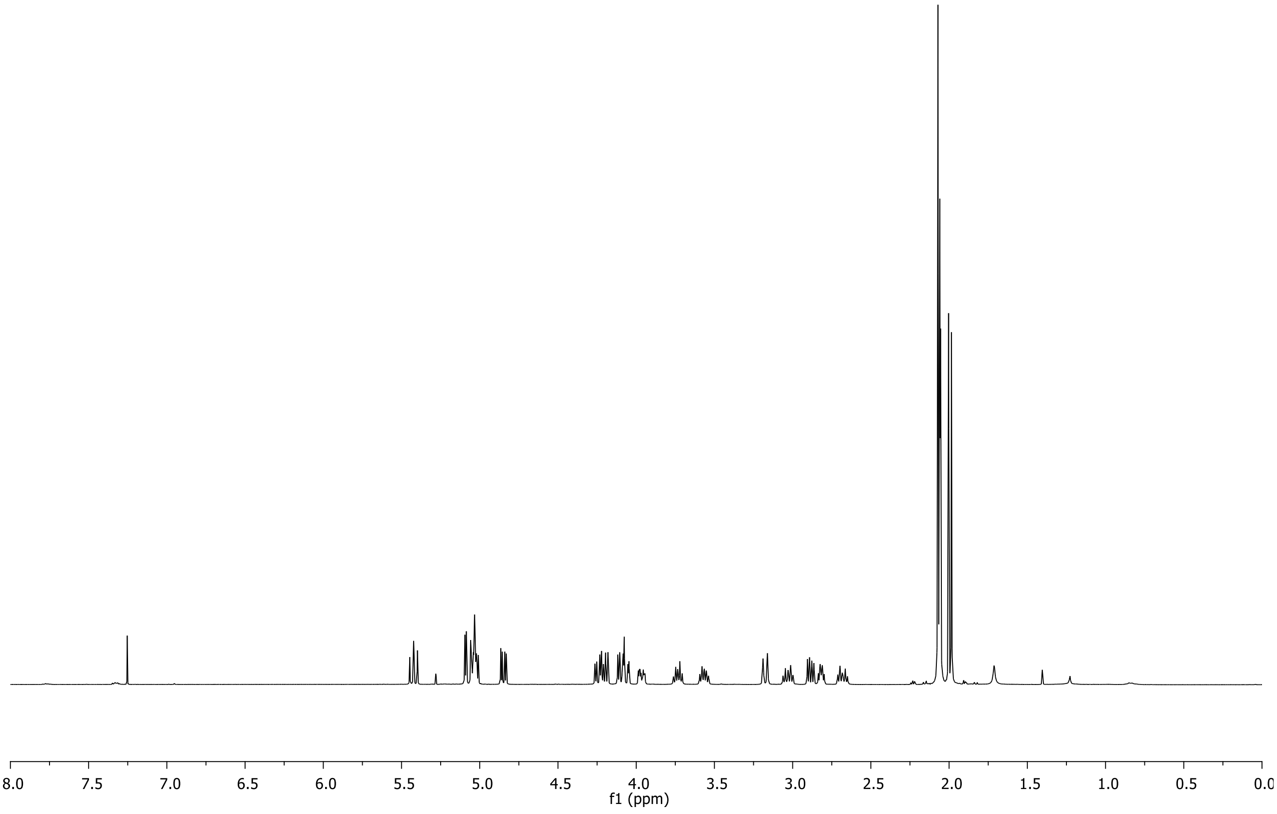

^{1}H-NMR spectrum of compound 24α (400 MHz, CDCl$_3$)

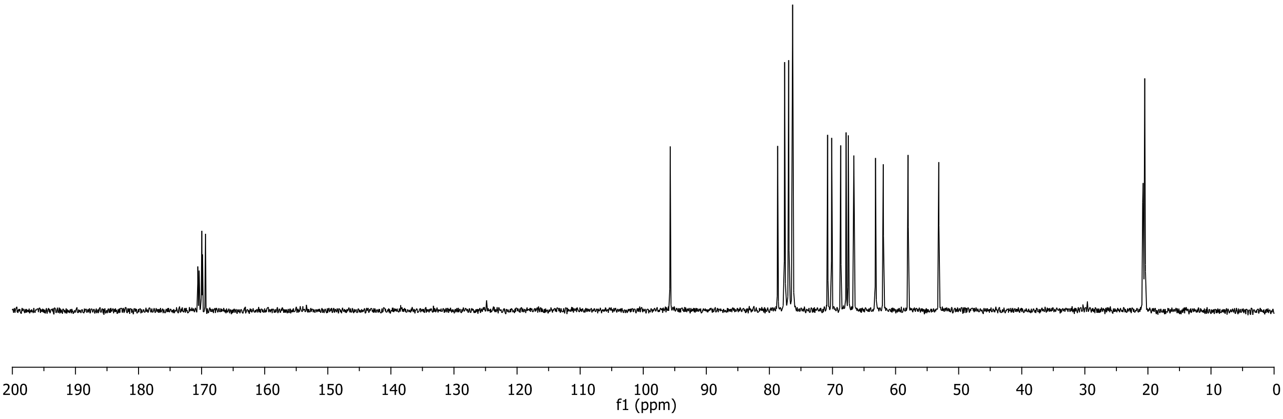

^{13}H-NMR spectrum of compound 24α (50 MHz, CDCl$_3$)

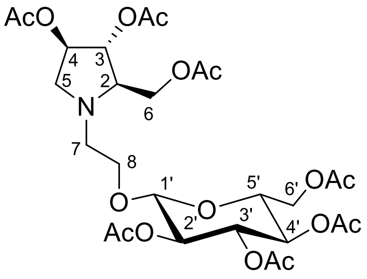

24β

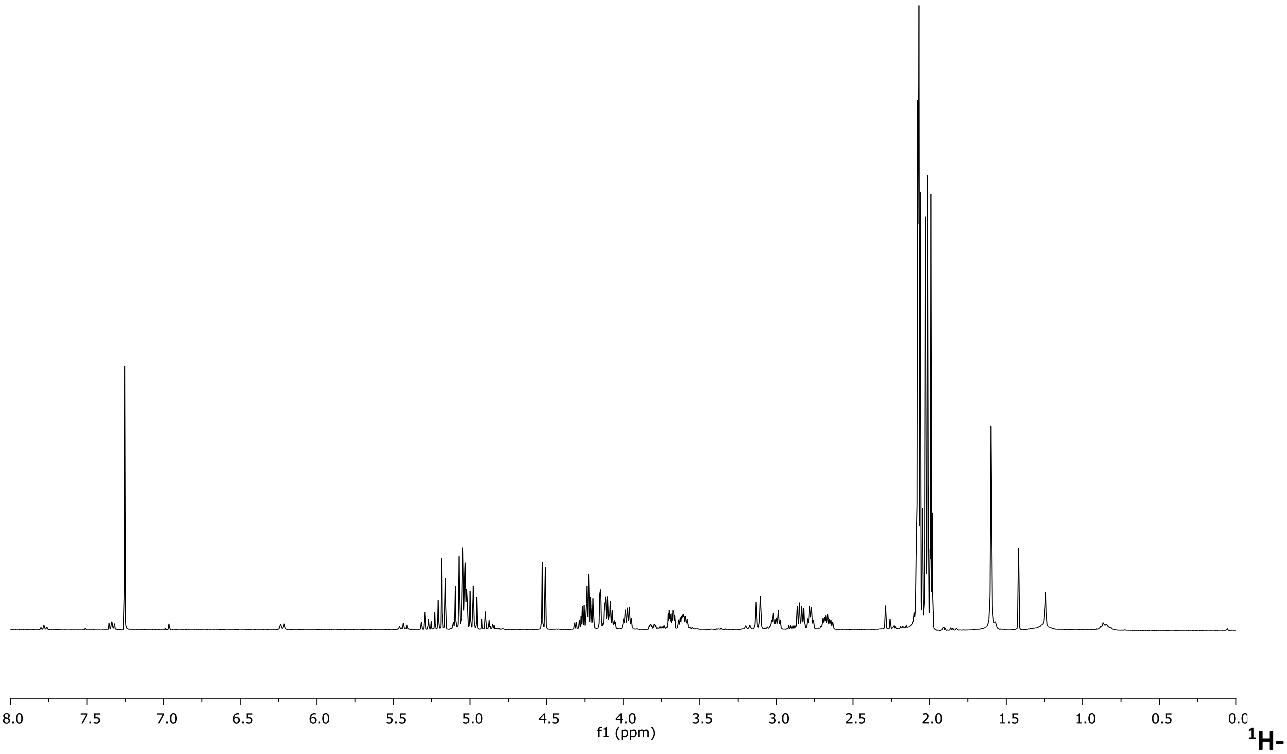

^{1}H-NMR spectrum of compound 24β (400 MHz, $CDCl_3$)

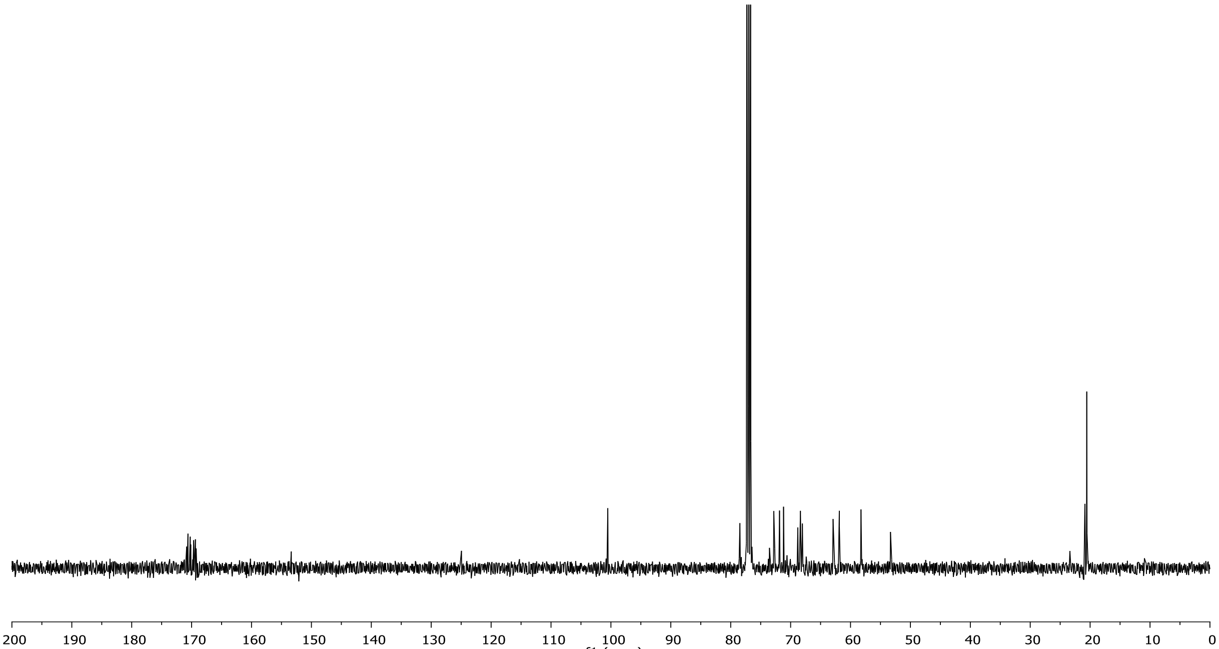

^{13}C-NMR spectrum of compound 24β (100 MHz, $CDCl_3$)

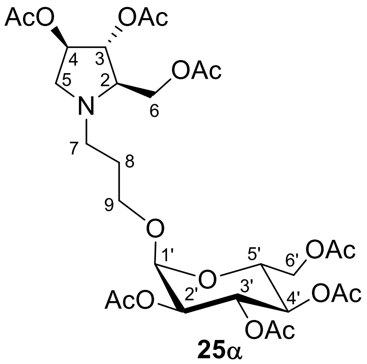

25α

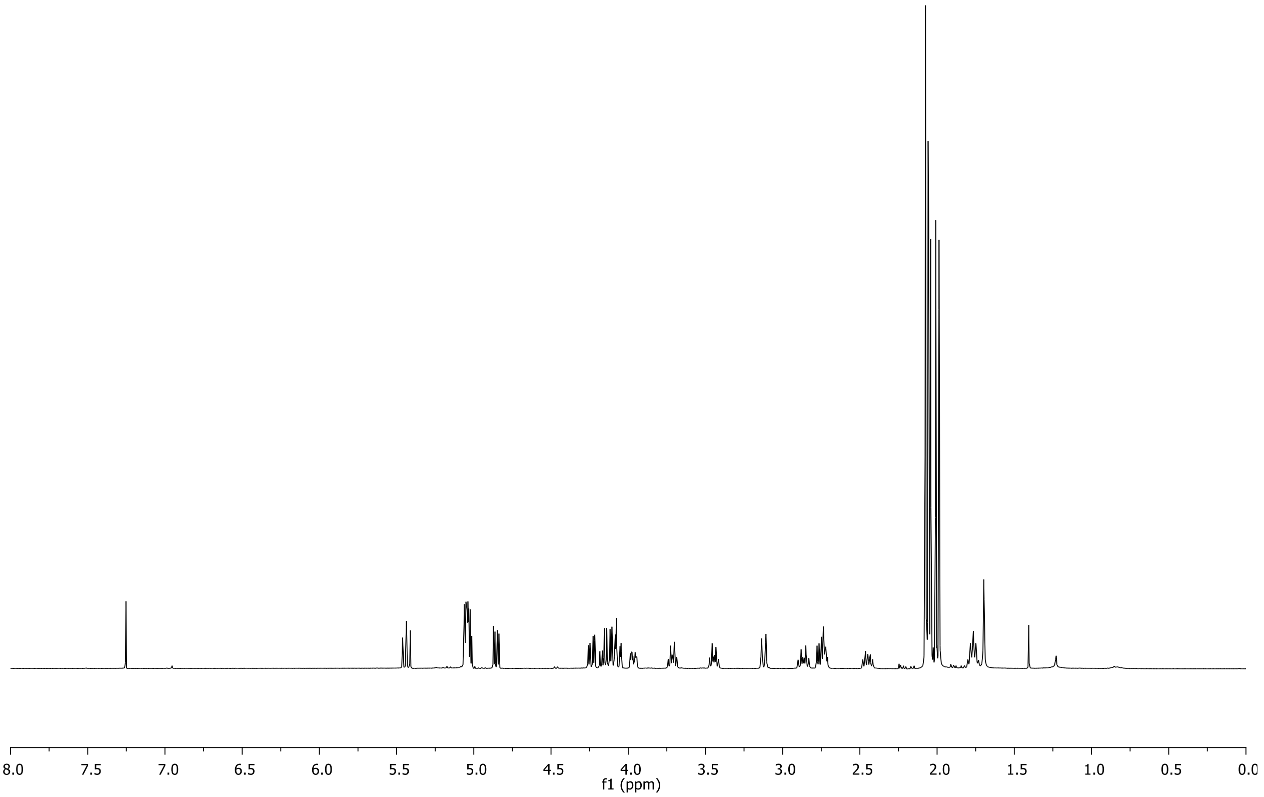

^{1}H-NMR spectrum of compound 25α (400 MHz, $CDCl_3$)

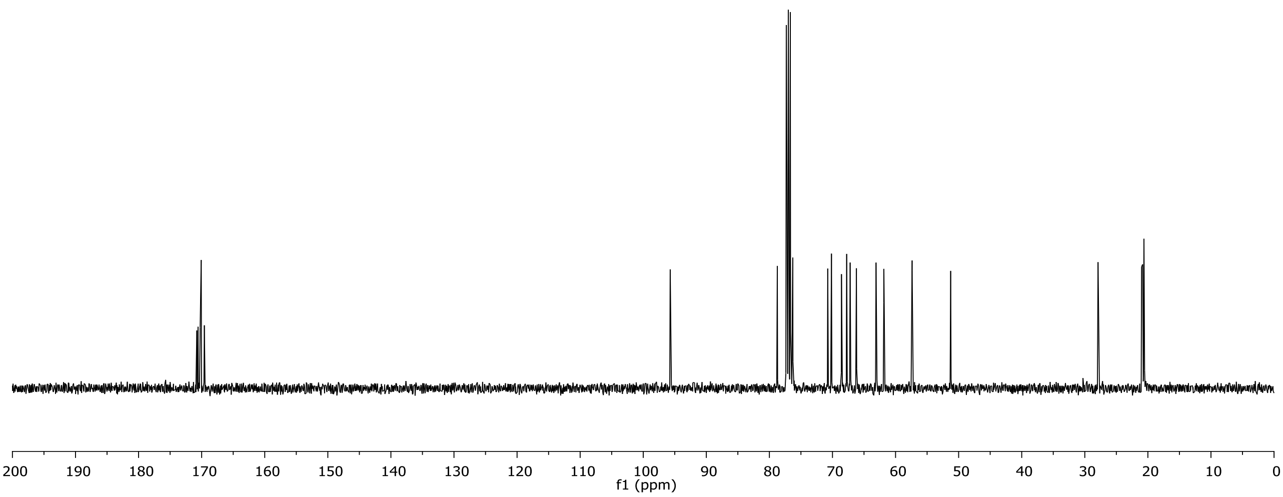

^{13}C-NMR spectrum of compound 25α (100 MHz, $CDCl_3$)

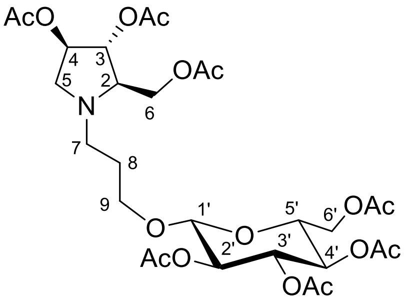

25β

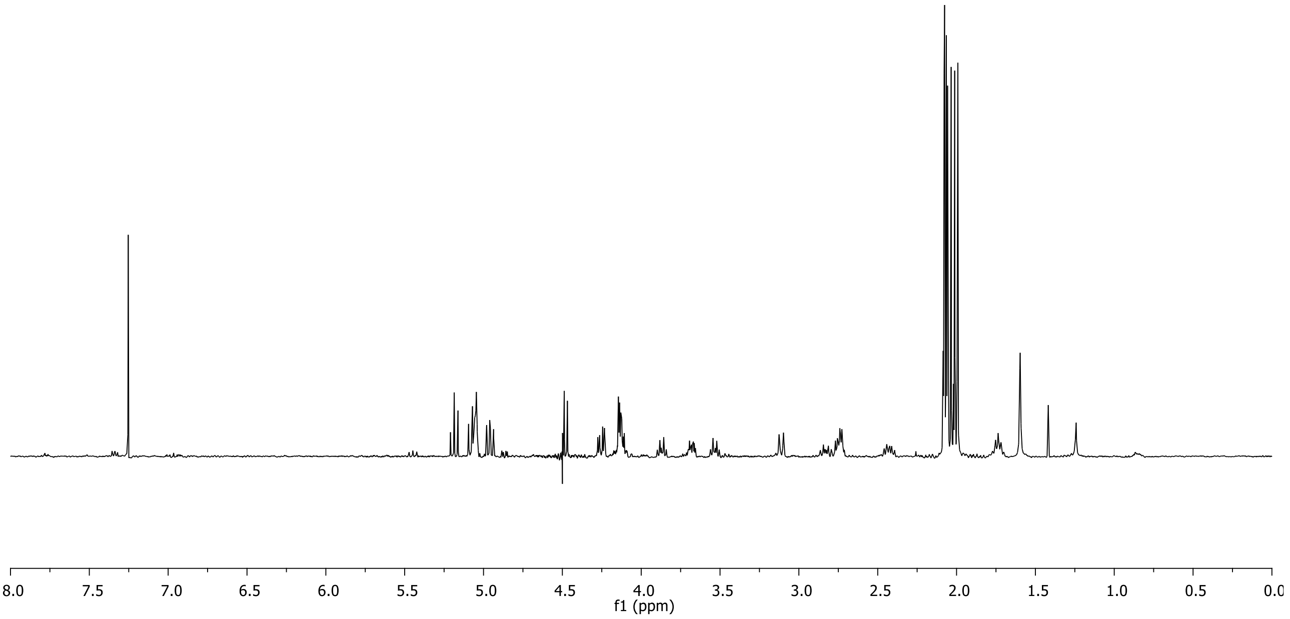

^{1}H-NMR spectrum of compound 25β (400 MHz, $CDCl_3$)

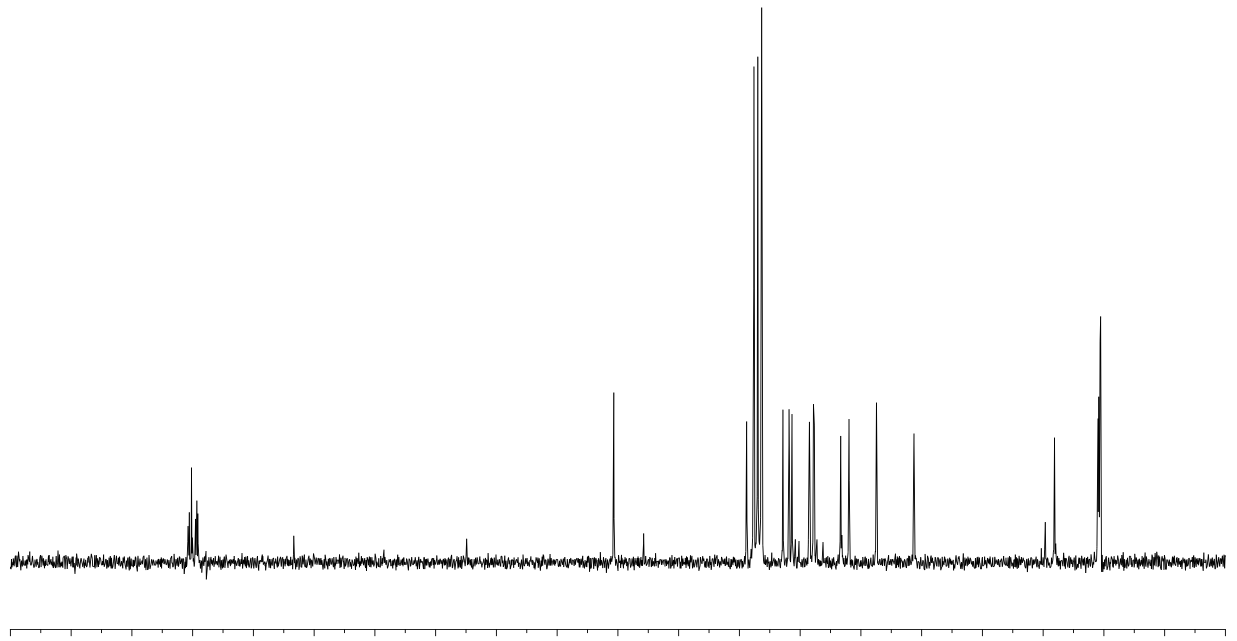

^{13}C-NMR spectrum of compound 25β (50 MHz, $CDCl_3$)

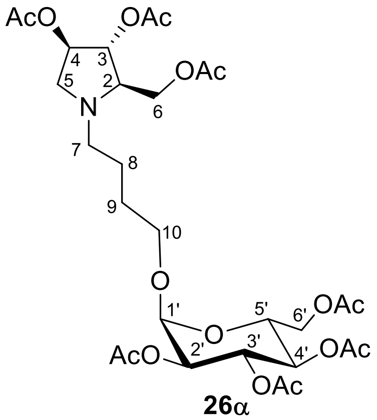

26α

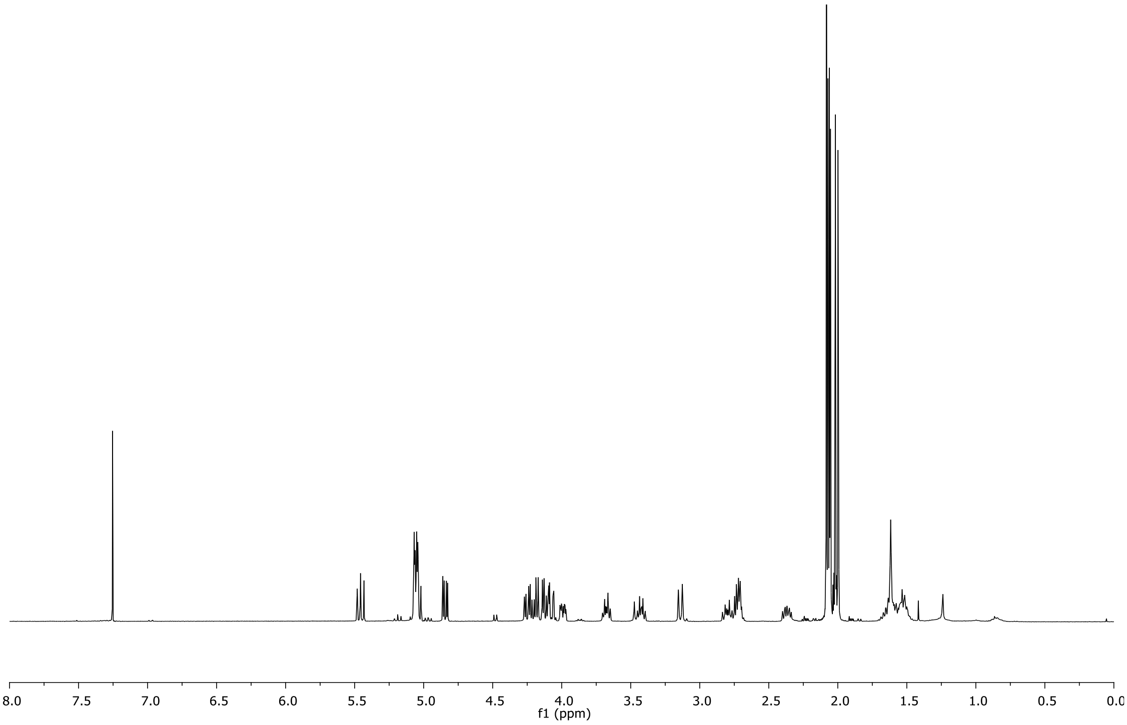

^{1}H-NMR spectrum of compound 26α (400 MHz, $CDCl_3$)

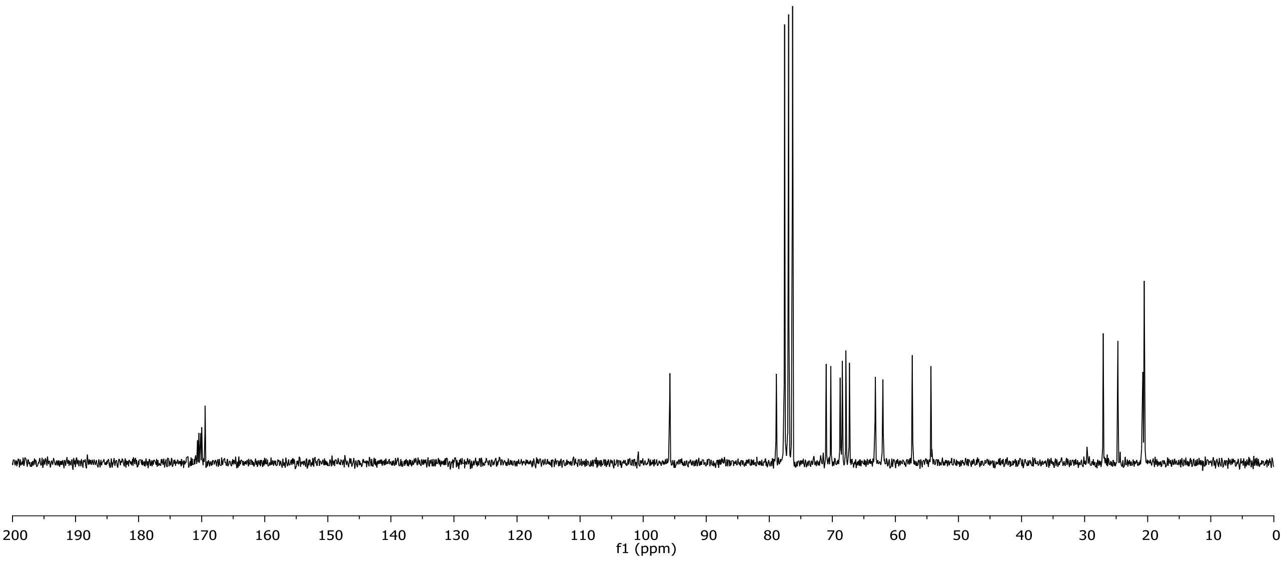

^{13}C-NMR spectrum of compound 26α (50 MHz, $CDCl_3$)

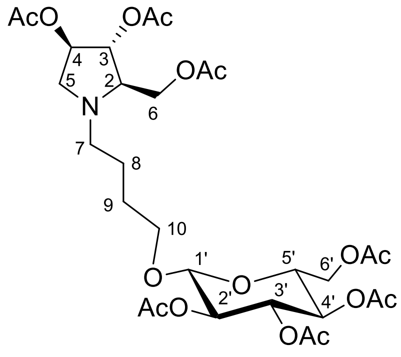

26β

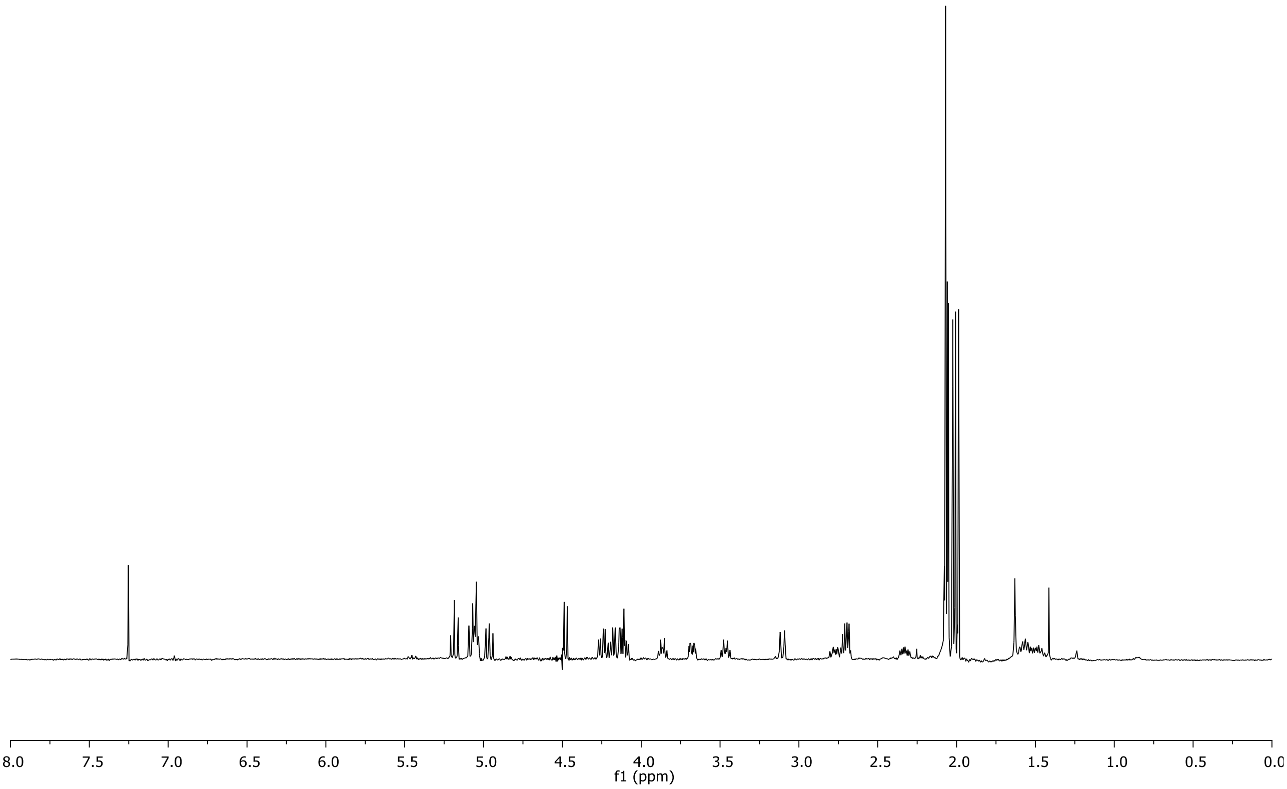

^{1}H-NMR spectrum of compound 26β (400 MHz, $CDCl_3$)

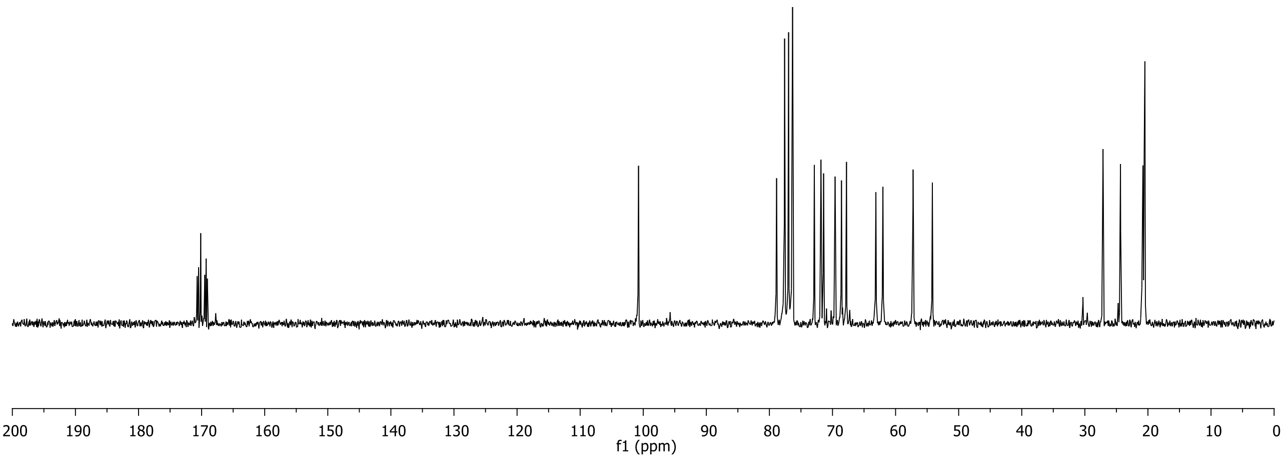

^{13}C-NMR spectrum of compound 26β (400 MHz, $CDCl_3$)

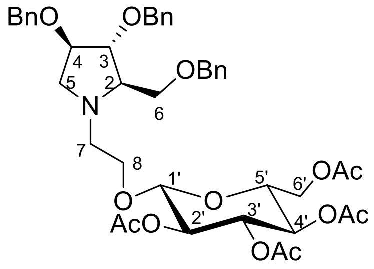

30

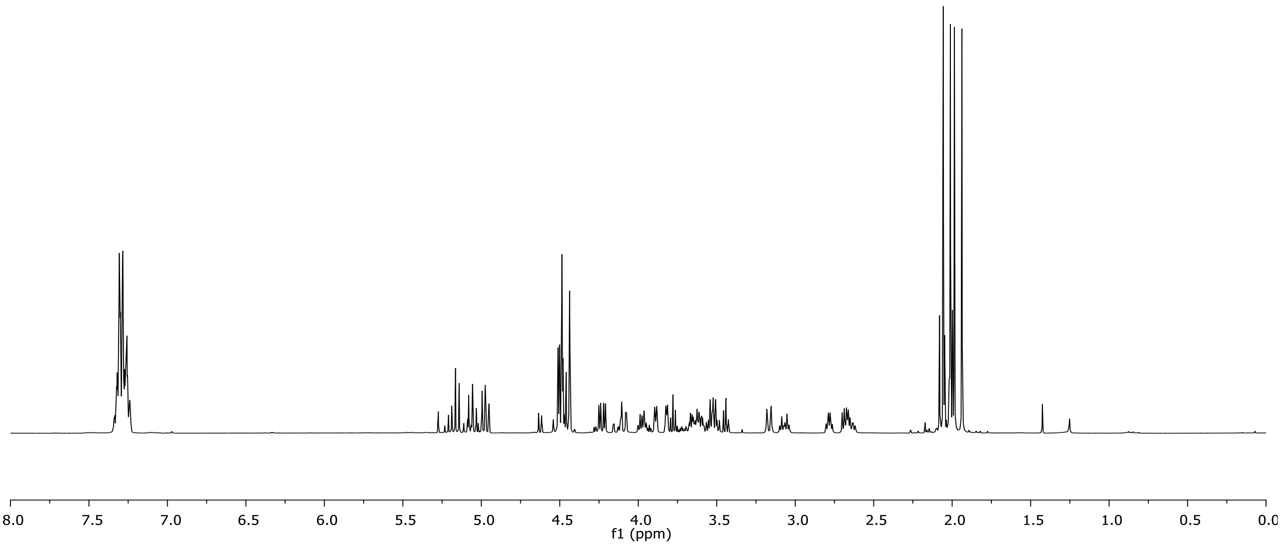

^{1}H-NMR spectrum of compound 30 (400 MHz, $CDCl_3$)

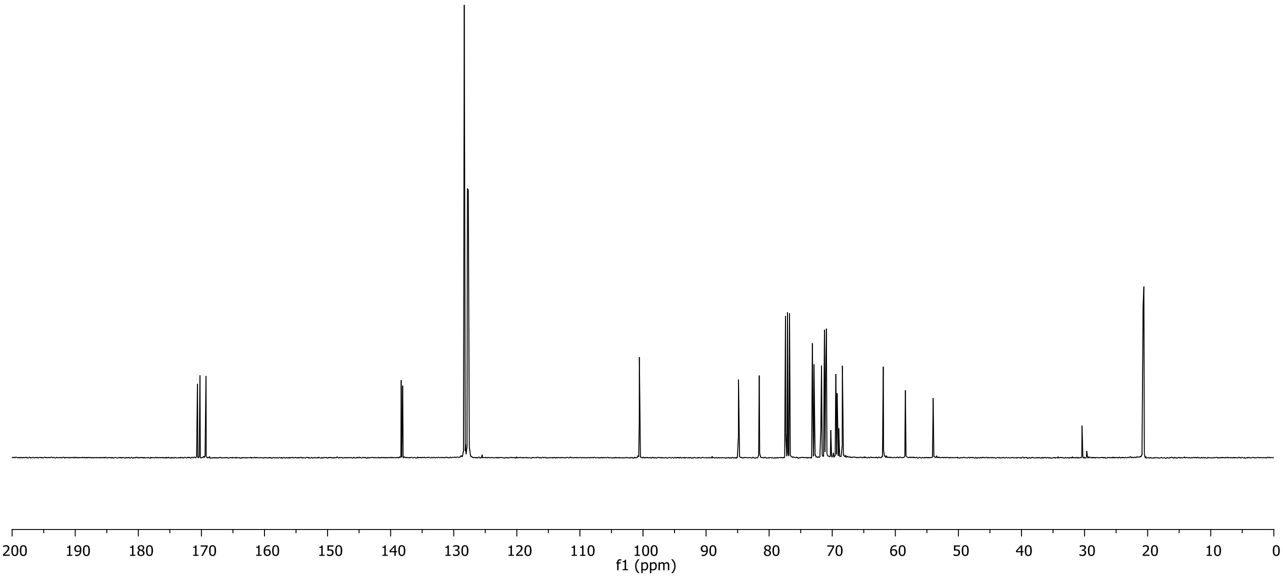

^{13}C-NMR spectrum of compound 30 (100 MHz, $CDCl_3$)

During an attempt of glucosylation of **29** (1 equiv) with **15** (1.5 equiv) performed in the presence of a high quantity of TMSOTf (2 equiv), we were able to isolate a fraction containing a glucosyl derivative deacetylated at C4'-OH (11% yield), identified by the ESI-MS signal at 736.55 $[M+H]^+$ and confirmed by ^1H and ^{13}C spectra. The signals of this deacetylated compound are present as impurities in the glycosylation compound **30** reported above.

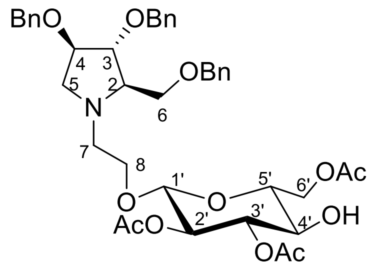

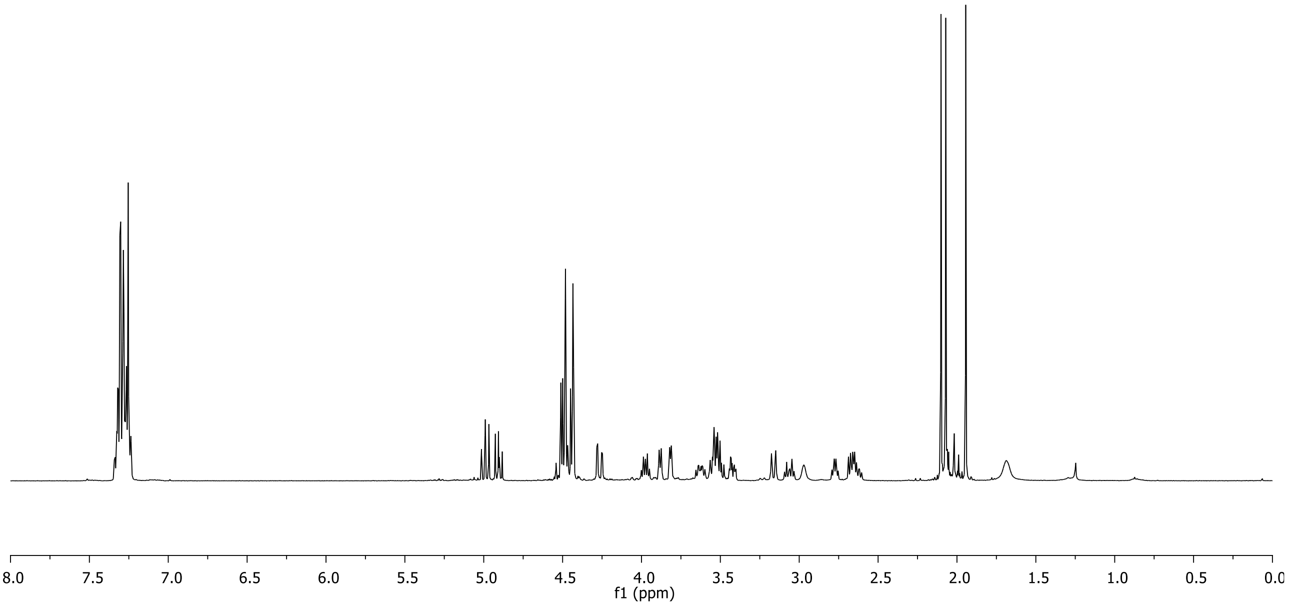

^{1}H-NMR spectrum (400 MHz, $CDCl_3$)

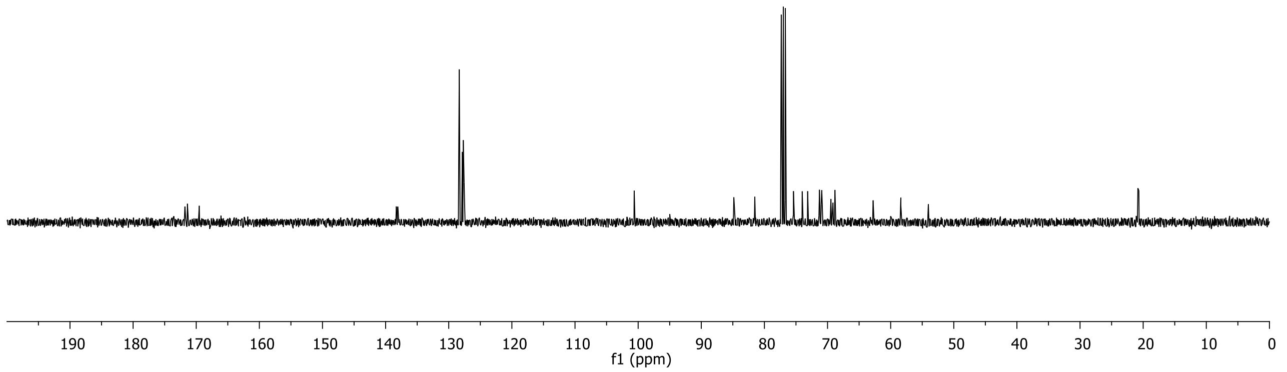

^{13}C-NMR spectrum (100 MHz, $CDCl_3$)

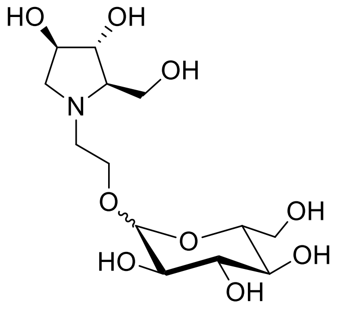

9α,β

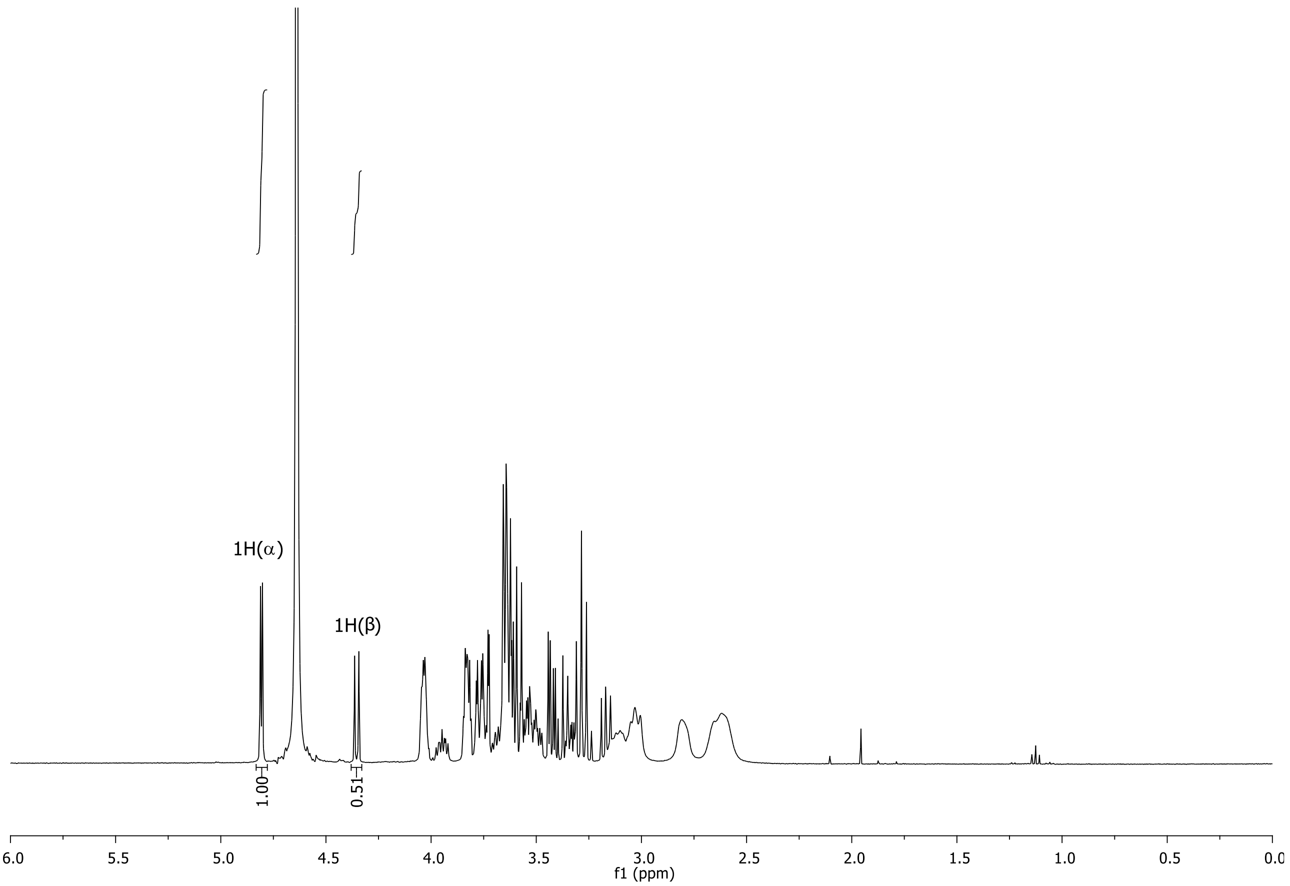

^{1}H-NMR spectrum of the mixture 9α,β (400 MHz, D_2O)

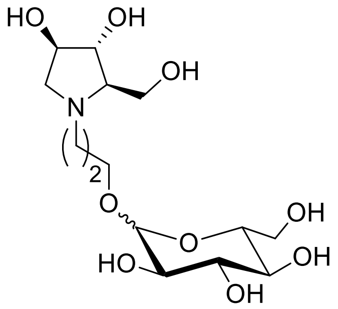

10α,β

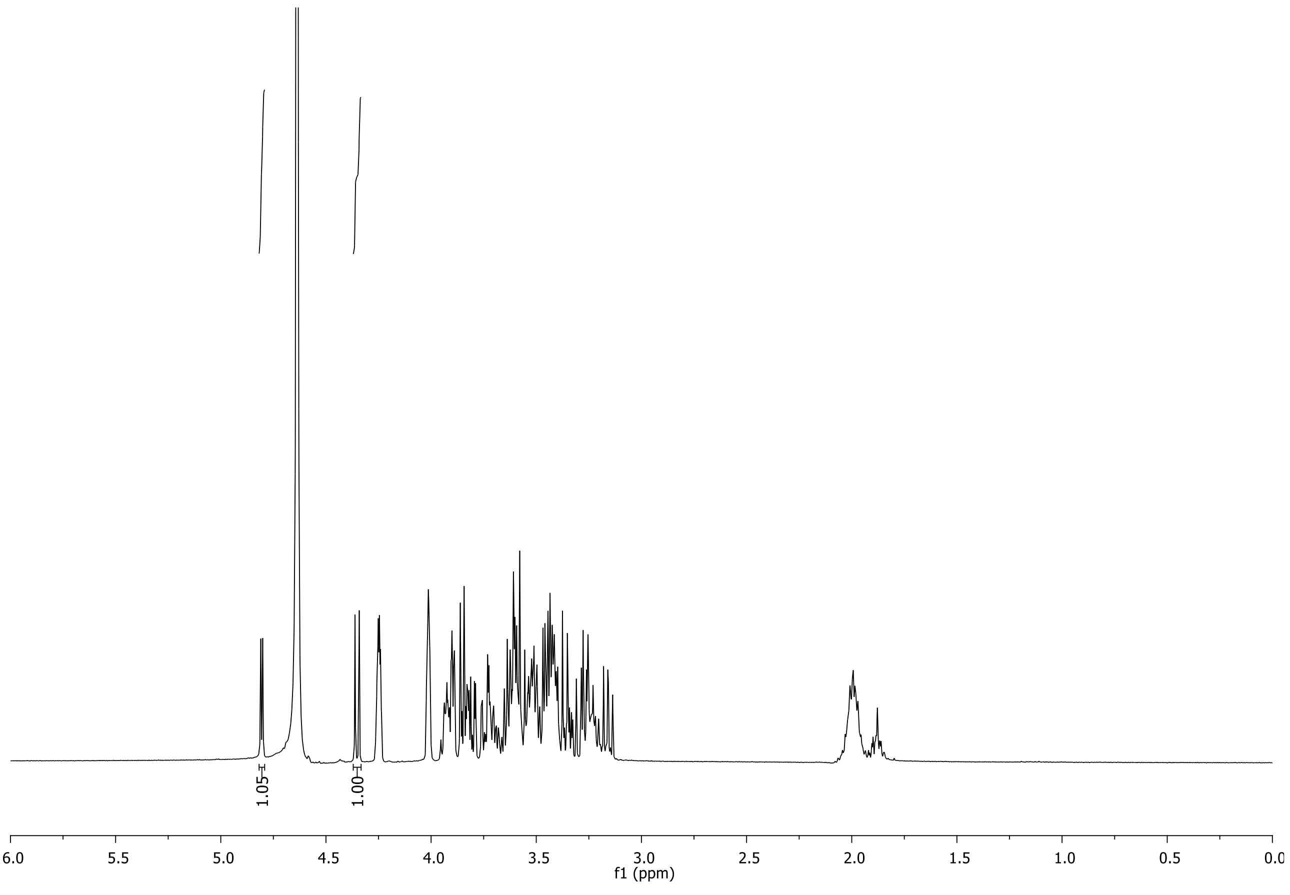

^{1}H-NMR spectrum of the mixture 10α,β (400 MHz, D_2O)

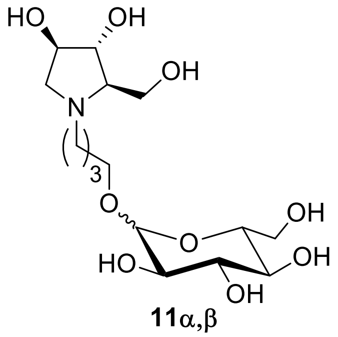

11α,β

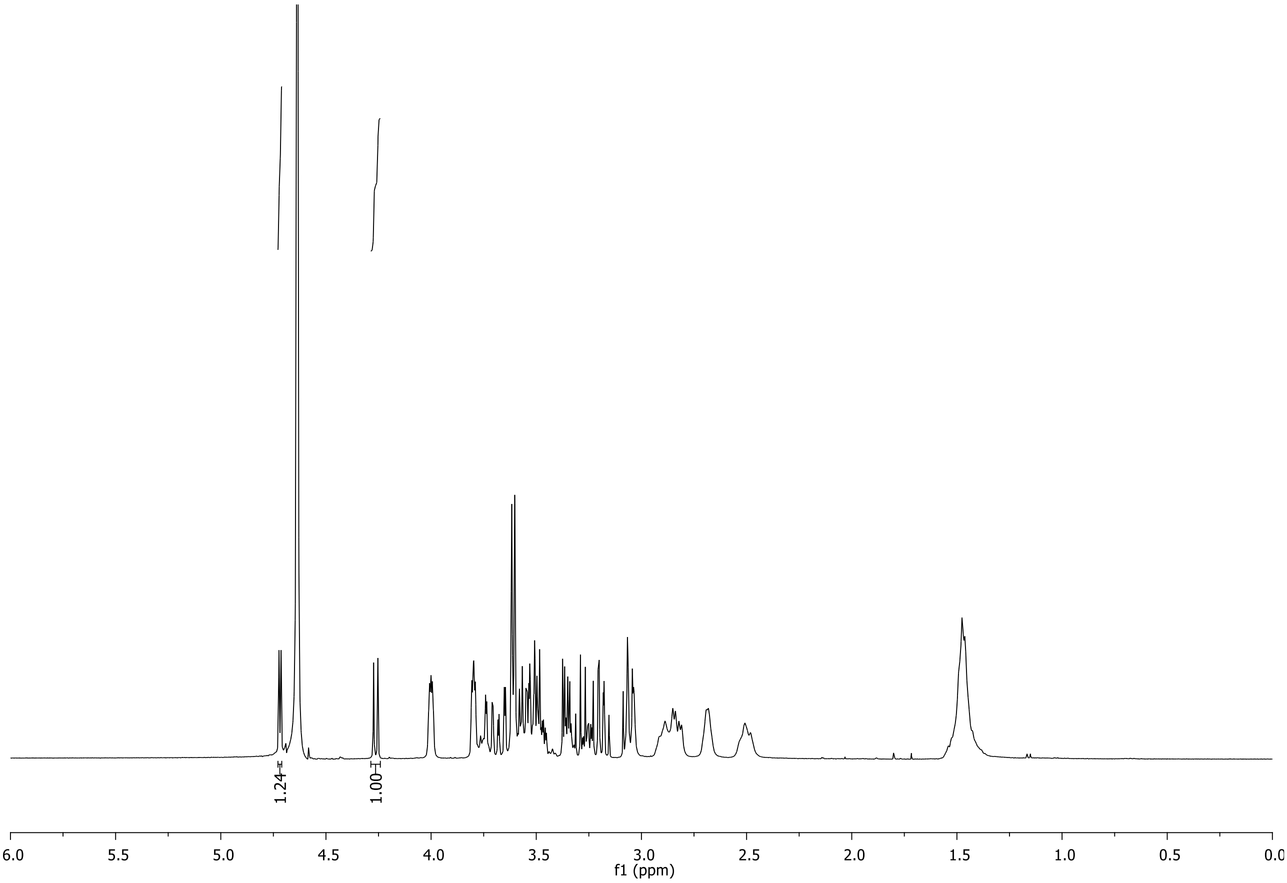

^{1}H-NMR spectrum of the mixture 11α,β (400 MHz, D_2O)

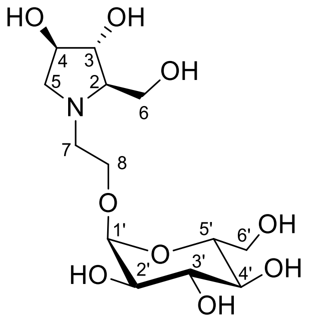

9α

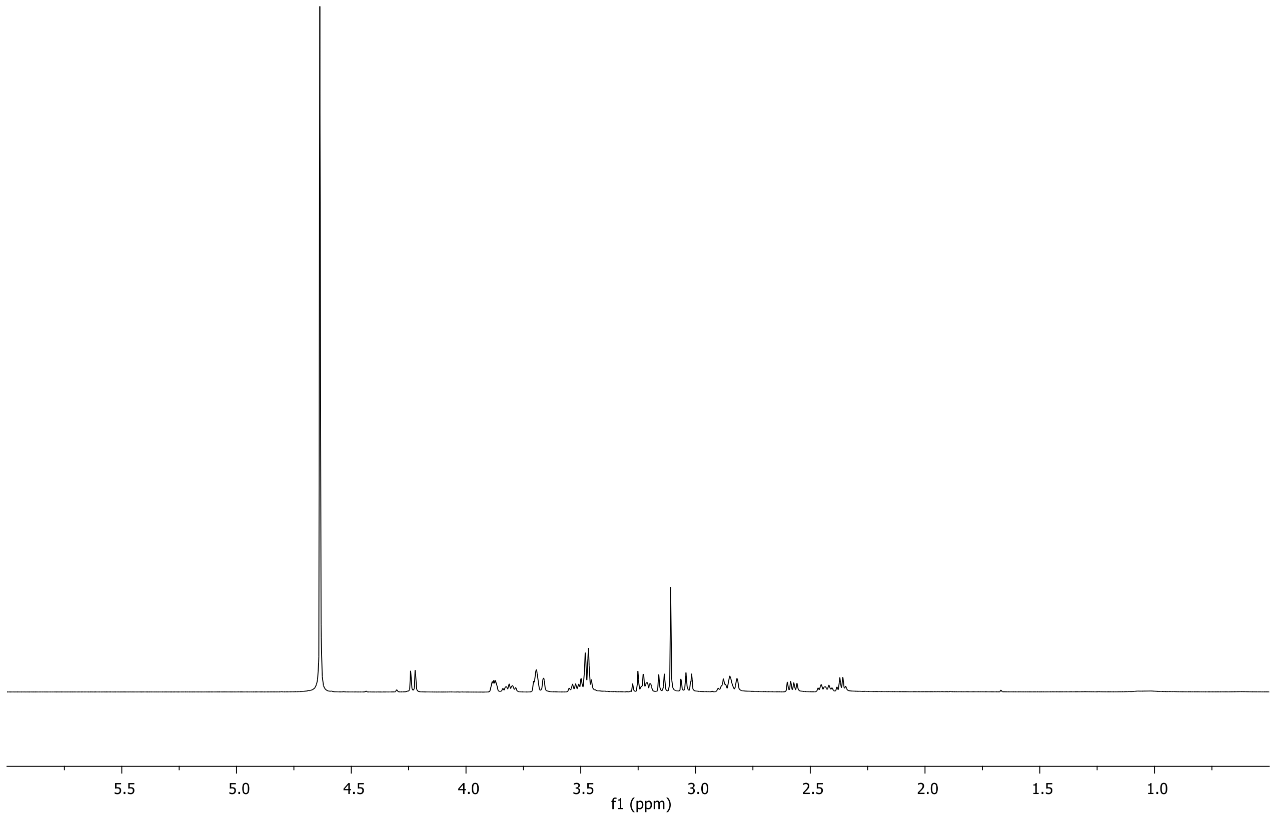

^{1}H-NMR spectrum of compounds 9α (400 MHz, D_2O)

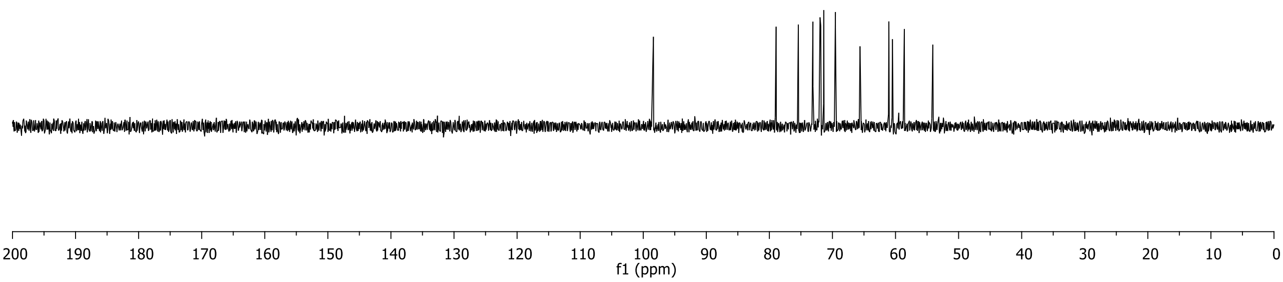

^{13}C-NMR spectrum of compounds 9α (100 MHz, D_2O)

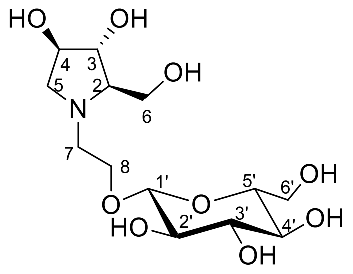

9β

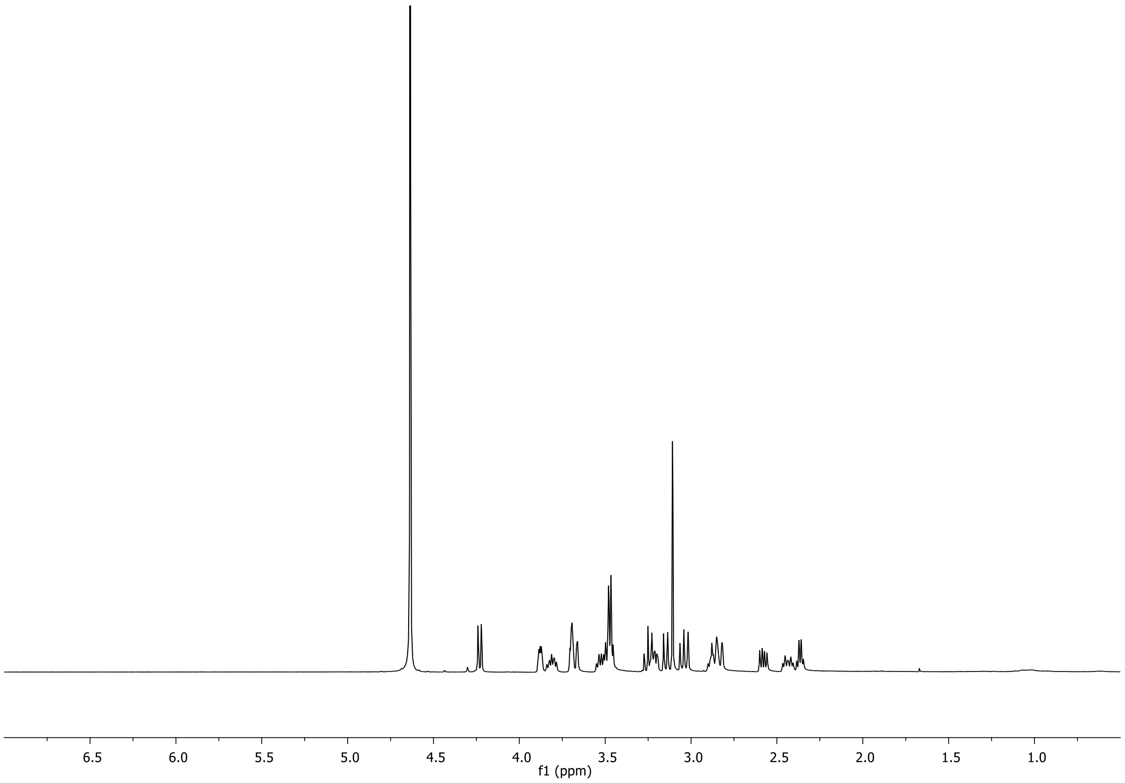

^{1}H-NMR spectrum of compounds 9β (400 MHz, D_2O)

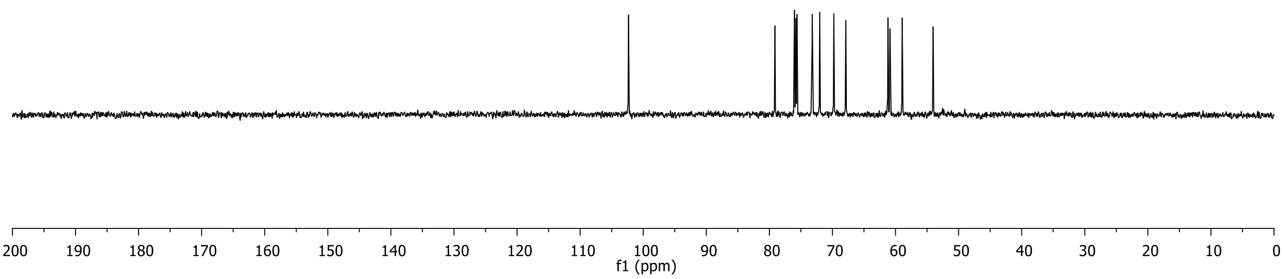

^{13}C-NMR spectrum of compounds 9β (100 MHz, D_2O)

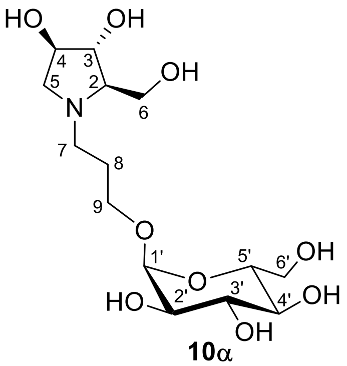

10α

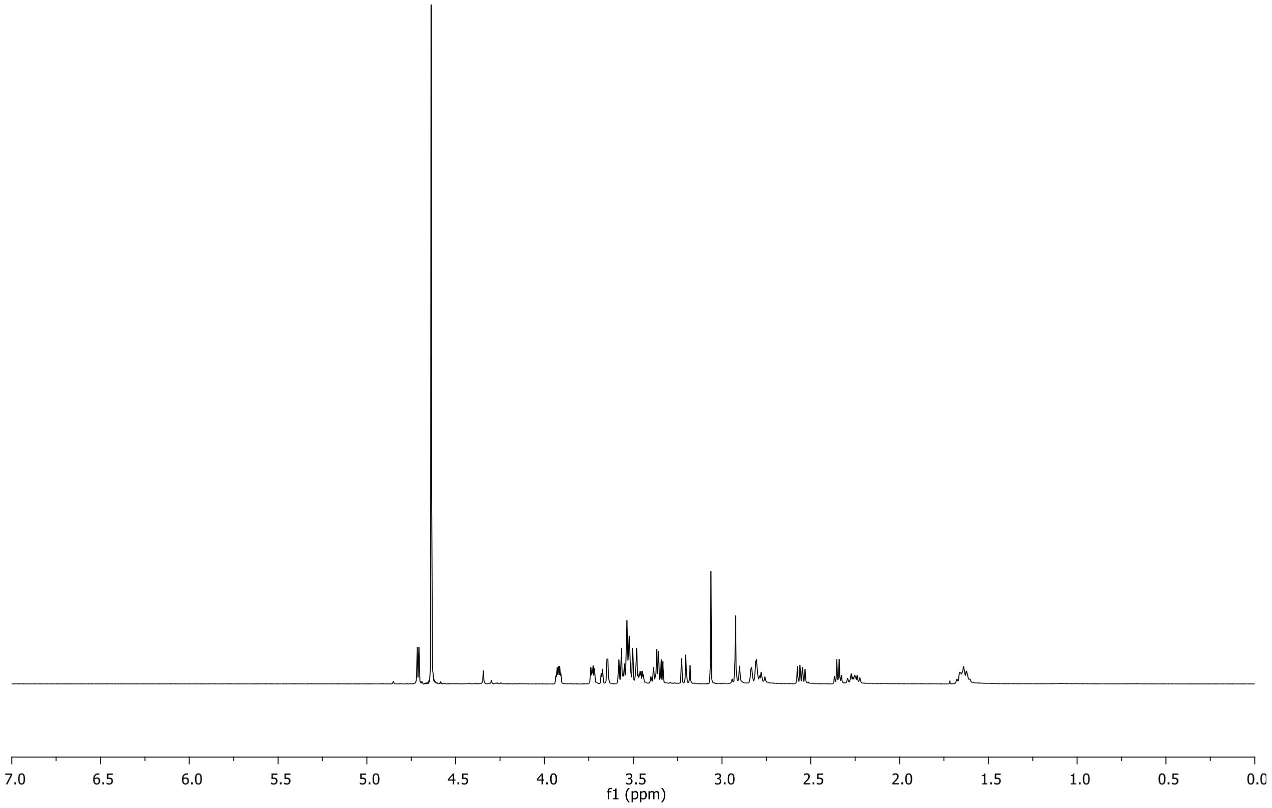

^{1}H-NMR spectrum of compounds 10α (400 MHz, D_2O)

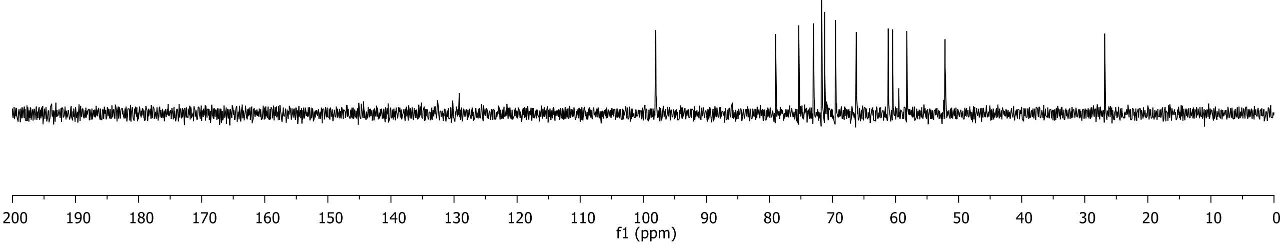

^{13}C-NMR spectrum of compounds 10α (100 MHz, D_2O)

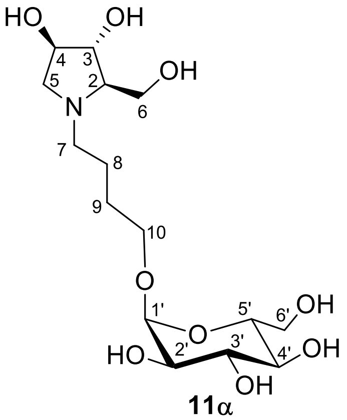

11α

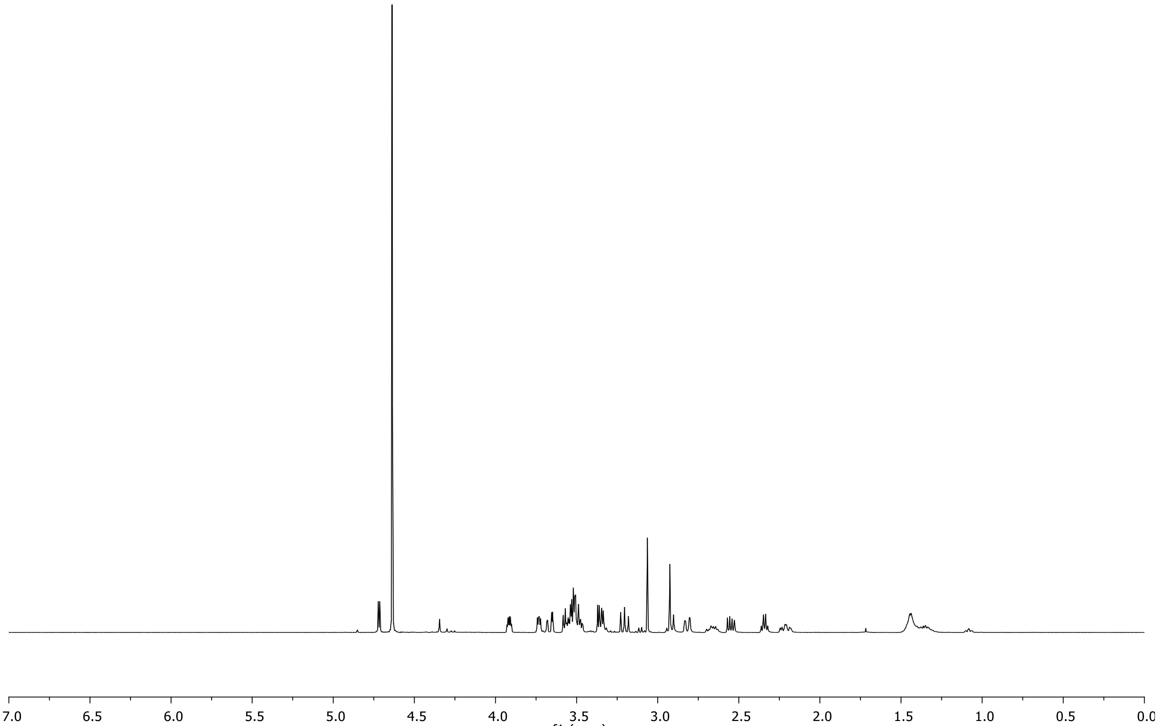

^{1}H-NMR spectrum of compounds 11α (400 MHz, D_2O)

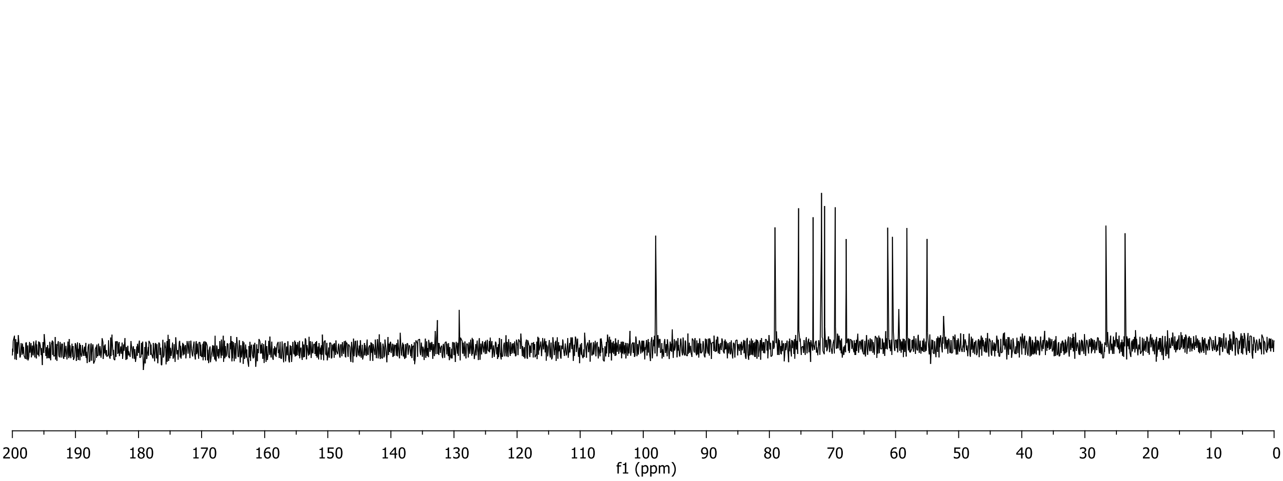

^{13}C-NMR spectrum of compounds 11α (100 MHz, D_2O)

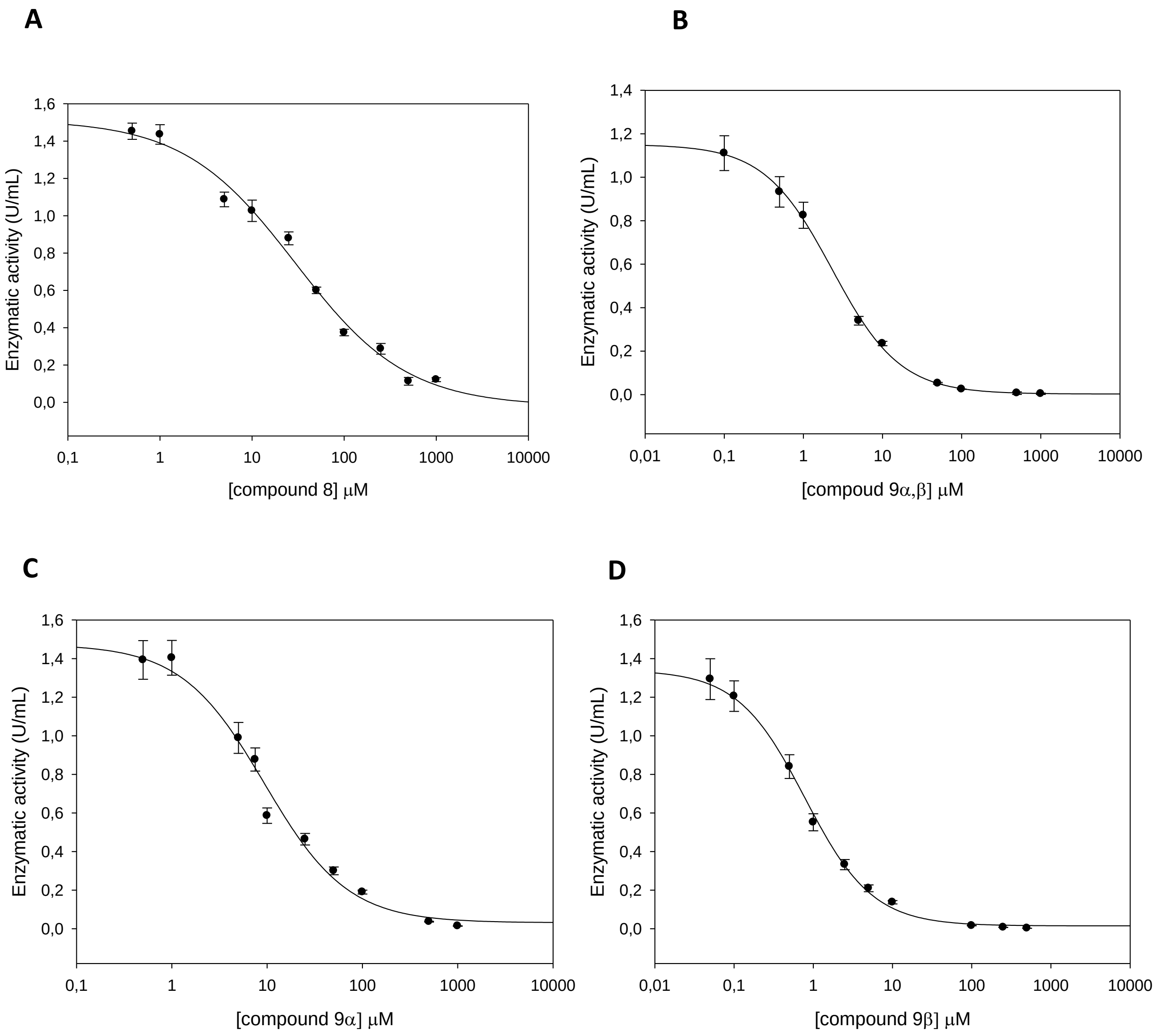

Figure S1. Dose-response curves of compounds **8** (A), **9α,β** (B)**, 9α** (C), **9β** (D) for insect trehalase

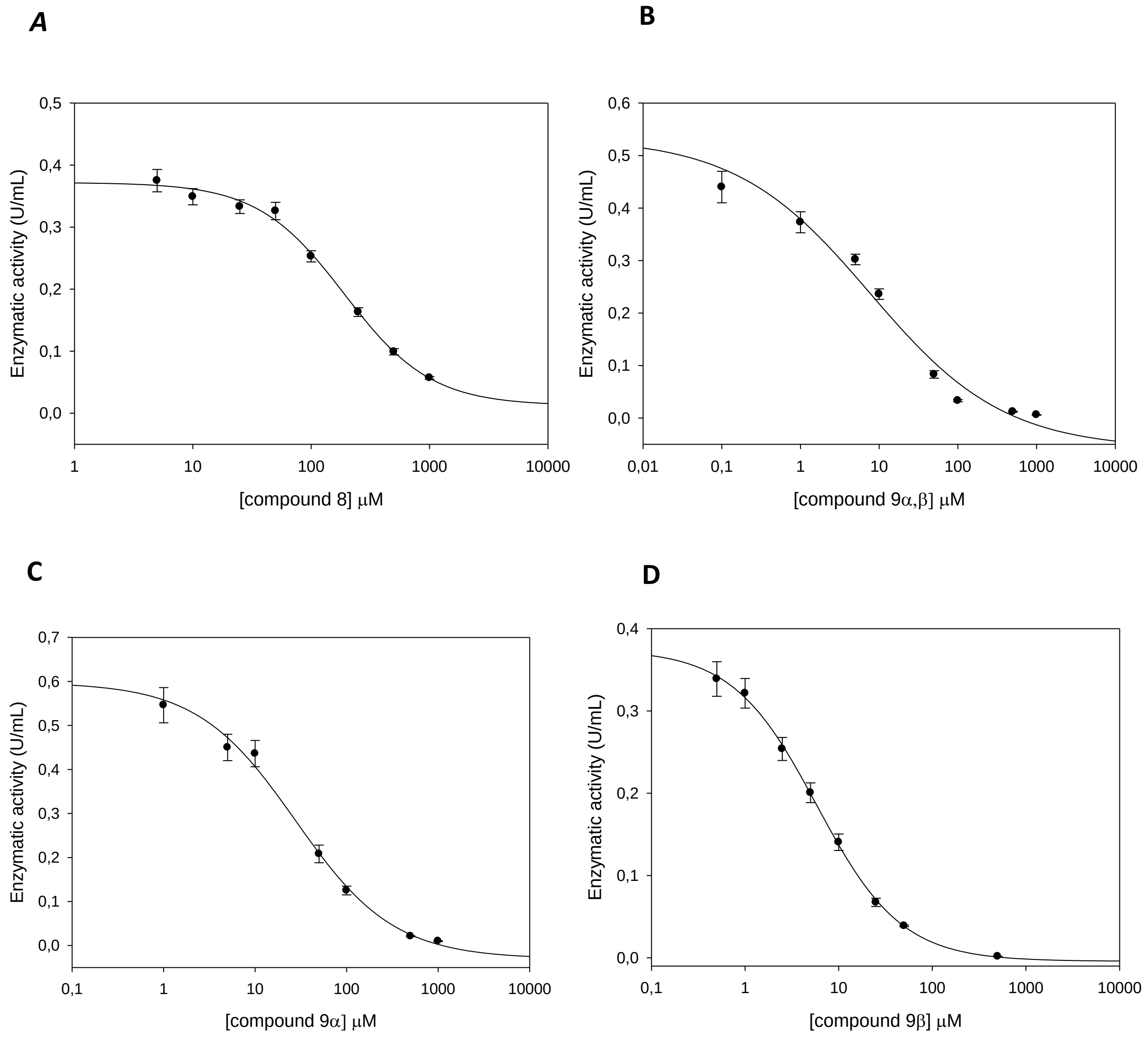

Figure S2. Dose-response curves of compounds **8** (A), **9α,β** (B)**, 9α** (C), **9β** (D) for porcine trehalase

A

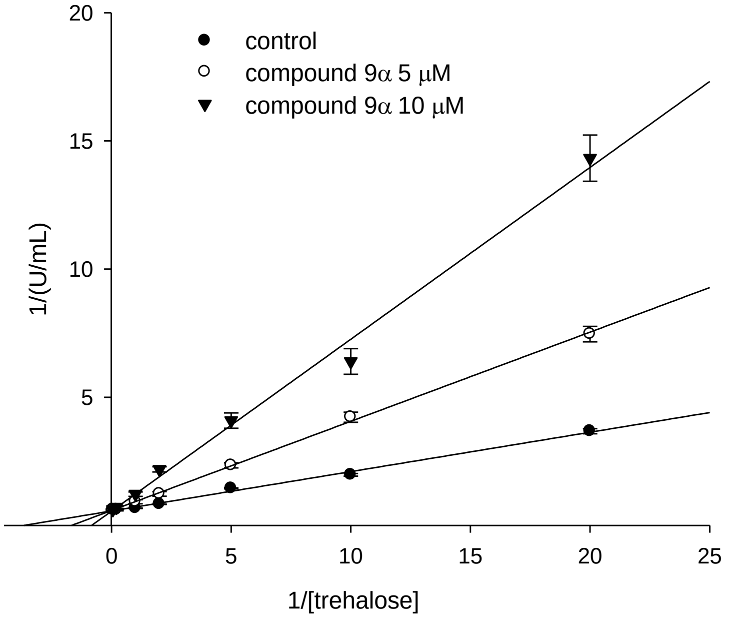

B

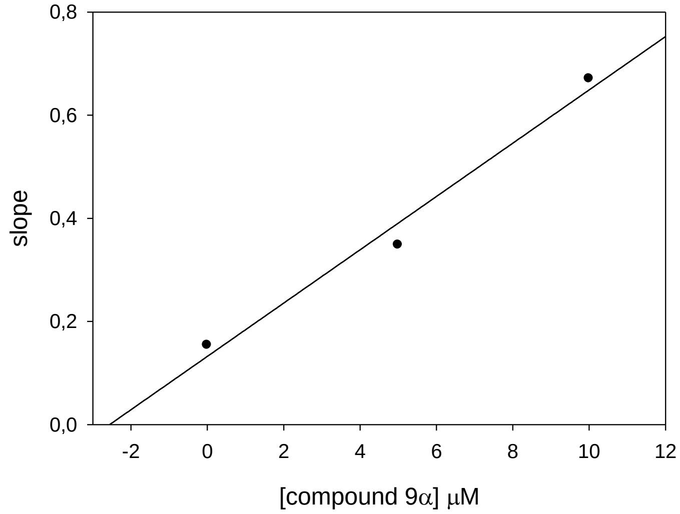

Figure S3. Inhibition kinetics of insect trehalase in the presence of compound **9α**. A) double reciprocal plot in the presence of two fixed inhibitor concentrations (5 and 10 μM); B) replot of the slopes of each reciprocal plot versus the corresponding inhibitor concentration.

A

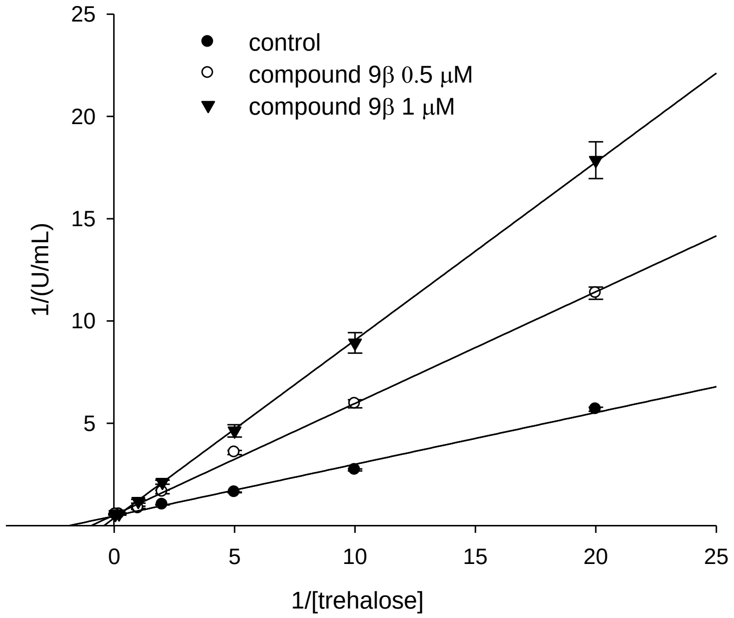

B

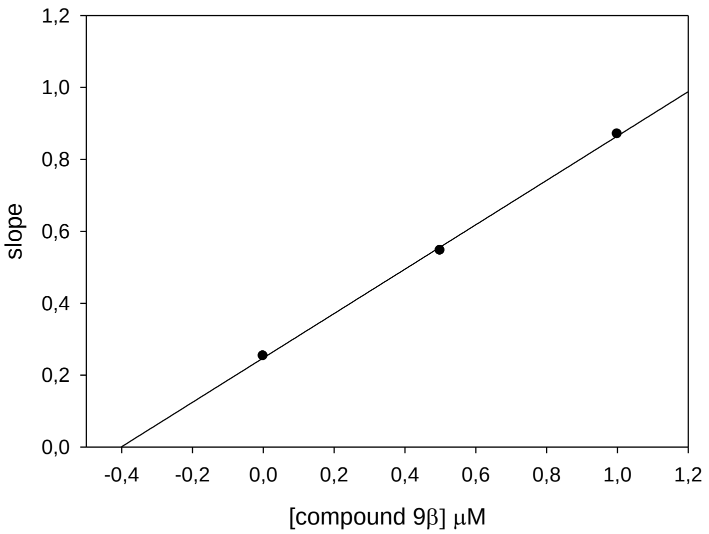

Figure S4. Inhibition kinetics of insect trehalase in the presence of compound **9β**. A) double reciprocal plot in the presence of two fixed inhibitor concentrations (0.5 and 1 μM); B) replot of the slopes of each reciprocal plot versus the corresponding inhibitor concentration.
